# Supplementary material for: Pathogenic missense protein variants affect different functional pathways and proteomic features than healthy population variants
Source: PLoS Biol. 2021 Apr 28;19(4):e3001207. doi: 10.1371/journal.pbio.3001207 (PMC8110273; doi:10.1371/journal.pbio.3001207)
Supplement: S1 Text — (PDF) [file pbio.3001207.s001.pdf]

S1 Text

Note on probabilistic methods to model variant enrichment

In this section we consider possibilities to model variant enrichment probabilistically, and explain our choice of parameters for our binomial formulation. A description of our formulation can be found in the Materials and Methods section of the manuscript. The formulation we adopted was taken from Porta-Pardo and colleagues [1], who applied this to identify protein interfaces enriched in cancer variants. We generalised this to look not only at interfaces, but also other regions of proteins.

Below we use the words "protein" and "region" to illustrate these ideas. In our work we generalised these to consider also the enrichment within individual proteins, domains and domain-types and sub-partitions defined within using e.g. solvent accessibility (surface, core) and protein disorder predictions; these concepts could substitute "protein" and "region" respectively as appropriate. For simplicity, in this explanation we consider a protein with complete structural coverage, i.e. every position could be assigned to any one of surface, core or interface.

There are at least two ways to formulate the problem of quantifying the enrichment of variants statistically:

Approach 1: Generating mutations

To model variant enrichment, here we start from a given region of a protein (let the size of this region be n), and view the *generation* of variants at different positions to be independent from one another. Assuming there is an equal probability to generate a variant at every position, we walk through each position in the region, and perform one "trial" to generate a mutation with probability p. Repeating this for all positions within the region, we finally ask what is the probability that k of those positions are mutated. (Fig A)

We can model X, the number of variants in the region, as a binomial random variable, with the probability density function:

$$P(X = k) = \binom{n}{k} p^k (1-p)^{n-k}$$

$P(X = k)$ is the probability that k variants have been found in this region. p is the expected chance of a given position to harbour a variant, which, under a frequentist approach, could be formulated as

$$p = \frac{\text{number of variants}}{\text{total number of residues}}$$

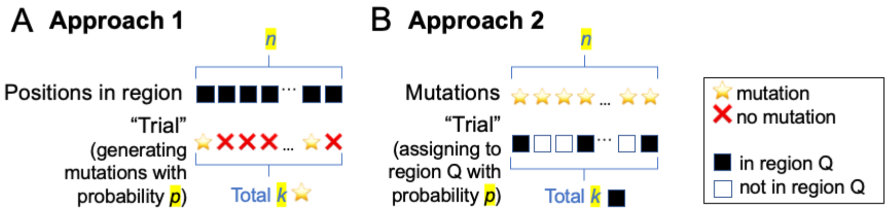

Fig A: Comparison of two binomial formulations in modelling variant distribution. Illustration of the two approaches for this hypothetical scenario of modelling variant enrichment of this protein in a defined region Q.

In this formulation each "trial" in a binomial process refers to generating a mutation at one given position in the region, and we ask whether this position harbours a variant. This formulation assumes an equal chance for every position to be mutated.

Approach 2: Distributing mutations

We took another way to approach this problem, starting with a given number (n) of mutations, and computing the probability that a subset (totalling k mutations) of them localise to a given region: each "trial" here refers to assigning a mutation to a region. The probability of assigning the mutation to our region of interest is p (Fig A).

Under a "null" scenario there is no bias towards localising to any region, therefore the expected probability p would simply be the ratio of the size of the region in question to the total size of the protein:

$$p = \frac{\text{number of residues in the region}}{\text{total number of residues}}$$

With these alternative definitions of n, k and p, this formulation explicitly models the distribution of mutations: we are constructing a probabilistic model to compare the observed *distribution* of variants across different regions to an expectation based only on the sizes of these regions. This "null" expectation is again a binomial process (therefore has the identical form as the $P(X = k)$ expression above): each "trial" refers to *assigning* a variant to any region of the protein.

Comparing these formulations of quantifying variant enrichment

The following table summarise the specification of the parameters of a binomial model (i.e. n, k and p) in both Approach 1 and Approach 2.

In the following, we demonstrate that these two formulations model the same problem, and exhibit the same numeric behaviour.

	Approach 1	Approach 2
n	Number of residues in the region	Total number of variants
k	Number of variants in the region	Number of variants in the region
p	$\frac{\text{Total number of variants}}{\text{total number of residues}}$	$\frac{\text{number of residues in the region}}{\text{total number of residues}}$

Table A: Summary of the parameters (n, k and p) of the binomial model in the two approaches explained above.

Cumulative distribution function (CDF)

The distribution of X is a discrete probability distribution modelled on a binomial process as detailed above, under either formulation of the problem. Using this one can quantify the *extent* to which variants are localised to a given region. $P(X = k)$ is a discrete probability of observing k variants. The extent of accumulating k variants would be the cumulative likelihood doing so, i.e. the sum of likelihoods to observe 0, 1, ... $k - 1$, k variants in this region:

$$P(X \leq k) = \sum_{i=1}^{k} \binom{n}{i} p^i (1-p)^{n-i}$$

This expression, $P(X \leq k)$, is equivalent to the *cumulative distribution function* (CDF) of the distribution of X. This CDF is taken in the manuscript as the Variant Enrichment Score (VES).

Notably, by definition, each $P(X = i)$ (for $i = 1, 2, ...k$) is bounded by [0, 1] and $\sum_{\forall i} P(X = i) = 1$, therefore the CDF monotonically increases as k increases and is bounded by [0, 1].

Fig B below shows a numerical and hypothetical example which demonstrates this point, with the VES (i.e. CDF) increases as the number of variants in the region increases:

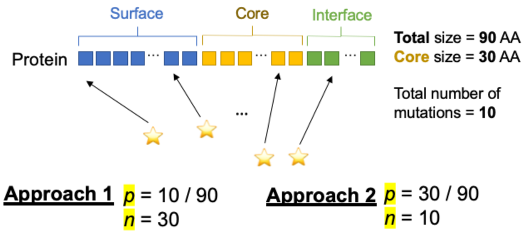

number of core mutations (k)	Approach 1	Approach 2
1	CDF = 0.1387	CDF = 0.1040
4	CDF = 0.7642	CDF = 0.7868
8	CDF = 0.9958	CDF = 0.9996

Fig B: Hypothetical example of different binomial formulations in calculating variant enrichment. (*left*) Set-up of this hypothetical scenario; we are interested in the variant enrichment of the core (size = 30 amino acids [AA]) of this 90-AA protein. 10 mutations have been mapped to this protein. The definitions of n and p of the two binomial formulations are listed. (*right*) Table showing the cumulative distributive function (CDF) calculated under the two binomial formulations for specific number of variants (k) observed in the core of this hypothetical protein.

Expected value

With the binomial process, one can write an expression for the expected value of the experiment:

$$E(X) = np$$

In our problem, this corresponds to the expected number of variants in the given region. Note that in either formulation, this expectation is identical; referring to Table A, for Approach 1,

$$E(X) = \text{Number of residues in the region} \times \frac{\text{number of variants}}{\text{total number of residues}}$$

For Approach 2 (i.e. our approach),

$$E(X) = \text{Number of variants} \times \frac{\text{number of residues in the region}}{\text{total number of residues}}$$

The two expressions are identical. Together with the behaviour of the CDF, this demonstrates the two formulations have identical numerical behaviours, and essentially models the same problem.

Differences

The way differences in protein (and therefore region) sizes are handled are slightly different, although as shown above the two approaches model variant enrichment similarly. For Approach 1, clearly n changes with protein size, as does the denominator (i.e. "total number of residues") of p (Table A). For example, for a larger protein with a larger region-of-interest, n increases and p decreases; this compensatory changes between n and p allow for consideration of variable protein/region sizes across different proteins. Approach 2 (the formulation we adopted), on the other hand, only considers the *assignment* of mutations to regions (Fig A). The changes in protein and region size are internalised within p (Table A). However, as shown above, this difference between the two formulations does not imply different numerical behaviours.

There are possible cases which result in different behaviours of variant enrichment statistics generated by the two formulations. If there are multiple different variants localised to the same position, the two formulations will treat such cases differently. In Approach 1, since each binomial "trial" is the generation of mutation, if multiple variants are mapped to the same position, they would only be counted once. This could potentially underestimate variant enrichment. This problem does not exist in Approach 2 (i.e. our formulation), as in that case the binomial experiment amounts to assigning mutations to regions. Multiple variants on the same position would be distributed to the same region. The following (Table B) illustrates the difference in CDF calculations in the two models, both for a real-life example (variant enrichment in the VHL protein in the COSMIC dataset) and a hypothetical, synthetic example based on the same protein.

As shown in Table B, Approach 2 is robust to the possibility of having multiple variants mapped on each position whereas Approach 1 underestimates the

	**VHL protein in COSMIC dataset**	**A hypothetical example**
Size (number of residues)	115 (Surface) & 36 (Core)	115 (Surface) & 36 (Core)
Number of variants	**Surface**: 117 on 60 positions<br>**Core**: 81 on 23 positions	**Surface**: 117 on 114 positions<br>**Core**: 81 on 5 positions
CDF	Approach 1<br>**Surface**: 0.305<br>**Core**: 0.894<br><br>Approach 2<br>**Surface**: 8.15×10^{-8}<br>**Core**: 1	Approach 1<br>**Surface**: 1<br>**Core**: 1.55×10^{-16}<br><br>Approach 2<br>**Surface**: 8.15×10^{-8}<br>**Core**: 1

Table B: Illustration of Approach 1 and Approach 2 applied on a real-life case of calculating variant enrichment (VHL protein in the COSMIC dataset) and a hypothetical, synthetic example designed based on the VHL protein. As shown in the table the protein in question is identical to one another (same sizes of core and surface regions), but in the hypothetical example variants in the core are concentrated in a small number of positions.

enrichment if variants are highly concentrated within a small number of positions. In this work we therefore chose Approach 2 (i.e. distributing/assigning variants as the binomial "trial"). Note this is *not* a new formulation; [1] used the same formulation to identify protein interfaces enriched in cancer variants. We generalise this to look not only at interfaces, but various partitions of the proteome.

References

[1] Porta-Pardo E, Garcia-Alonso L, Hrabe T, Dopazo J, Godzik A. A Pan-Cancer Catalogue of Cancer Driver Protein Interaction Interfaces. PLoS computational biology. 2015;11(10):e1004518. doi:10.1371/journal.pcbi.1004518.
