# Supplementary material for: Pathogenic missense protein variants affect different functional pathways and proteomic features than healthy population variants
Source: PLoS Biol. 2021 Apr 28;19(4):e3001207. doi: 10.1371/journal.pbio.3001207 (PMC8110273; doi:10.1371/journal.pbio.3001207)
Supplement: S2 Text — (PDF) [file pbio.3001207.s002.pdf]

# S2 Text

# Supplementary Methods

## 1 Data sources

### 1.1 Variant data

ClinVar (dbSNP BUILD ID 149) variant data [1], COSMIC coding mutations (v80) [2] and gnomAD exome data [3], all mapped to the GRCh37 genome build, were obtained in variant call format (VCF). The ClinVar dataset contains variants submitted through clinical channels. Only variants with CLINSIG codes 4 and 5 (i.e. those classified as "probably pathogenic" or "pathogenic") were selected for further analysis. To ensure the quality of our dataset, we selected only variants with "variant suspect reason code" of 0 (unspecified). Additionally, all variants labelled as being somatic were filtered from this dataset. All variant datasets were mapped to Ensembl protein sequences [4] using the Variant Effect Predictor (VEP) [5], and further mapped to canonical UniProt sequences and the respective structures/homologues.

### 1.2 Protein-protein interaction network

A large non-redundant protein-protein interaction network (UniPPIN) [6] was used. This incorporates non-redundant interactions amalgamated from IntAct [7], BioGRID [8], STRING [9], DIP [10] and HPRD [11], as well as recent large-scale experimental studies [12, 13, 14].

### 1.3 Protein sequences and structures

The biounit database of the Protein Data Bank (PDB) was downloaded on 28/04/2017. For mapping purposes, in this study, both the canonical UniProt human protein sequences [15] (for mapping to structures and protein-protein interaction networks) and Ensembl protein sequences [4] (for mapping variant datasets) were used.

### 1.4 Gene and protein annotations

Gene sets for KEGG pathways were obtained from the MSigDB database [16]. Oncogene and tumour suppressor gene annotations were taken from Supplementary Table S2A from Vogelstein and colleagues [17]. Cancer genes were taken from the Cancer Gene Census (CGC) (COSMIC v84). Genes from both tiers 1 and 2 were included. Conversions between gene symbols, Entrez gene

identifiers and UniProt accession numbers were performed using the biomartR package [18, 19]. A list of DNA-binding domains was obtained from the review by Vaquerizas and colleagues [20]. These domains were mapped from InterPro [21] IDs to Pfam IDs using conversion tables in Pfam (v31).

### 1.5 Protein-drug interaction mapping

A mapping of protein-drug interactions was obtained from DrugBank (v5.0.11) [22] (under "Target Drug-UniProt Links") and filtered for human proteins. Drugs were mapped to a Pfam domain-type if at least one domain of that type occurs in a protein a drug is known to interact with. It is, of course, possible that a drug may only interact directly with another domain-type within the protein. However, this approach was chosen due to the fact that if only domain-drug interactions with supporting structural information are accepted, the data becomes both sparse and biased towards structurally resolved domains.

### 1.6 Protemics and transcriptomics data

Protein thermal stability, abundance and half-life data were obtained from separate large-scale studies [23, 24, 25] as detailed in the main text. Gene expression quantification (Reads Per Kilobase of transcript per Million mapped reads [RPKM]) counts per sample (v6p) was downloaded from the GTEx portal [26] and grouped by tissue, according to the sample metadata provided. For each tissue type, we quantified the gene-wise proportion of samples with an RPKM equal to zero. Only those genes with zero counts in $< 10$ % of samples were retained for our analysis.

## 2 ZoomVar Database

### 2.1 Identification of resolved structures/homologues

Canonical UniProt human protein sequences were assigned resolved structures/homologues from the PDB biounit database [27] using BLAST [28]. BLAST searches were carried out using both full-length protein sequences and domain sequences, which were defined by scanning UniProt sequences against the PFAM seed library [29] using HMMER [30]. Hits were only accepted with sequence identity $> 30$ % and E-value $< 0.001$. T-COFFEE [31] was used to obtain a per-residue mapping of queries to structure hits. The quotient solvent accessible surface area [Q(SASA)] of each structure residue was computed using POPS [32].

### 2.2 Mapping of Ensembl proteins

Ensembl protein sequences were mapped to UniProt protein sequences [15], using UniProt ID mapping. Additionally, if UniProt and Ensembl sequences were not of the same length, the sequences were aligned using T-COFFEE [31] to obtain a per-residue mapping. Stretcher [33] was used to align those sequences which were too long to align using T-COFFEE.

### 2.3 Determination of per-residue binding partners

A protein may interact with multiple other proteins. For each of these interactions, a maximum of 10 corresponding best hits (ordered by HomPPI defined score [34]), located in the best populated zone, were considered. If a residue was located at the interaction interface, in at least half of these structures, it was annotated as interacting with that specific protein, otherwise it was annotated as non-interacting.

## 3 Calculation of protein topological network features

Graph representations for protein structures, with mapped missense variants, were constructed. Here protein C$\alpha$ atoms are represented by nodes in the graph, and nodes are connected if C$\alpha$'s are $< 10$ Å apart in 3D space. Similar graph representations are routinely used to create elastic network models of proteins. One class of such models is the Gaussian network model (GNM); the default cut-off distance used by GNMs is commonly 10 Å [35]. Therefore we use this cut-off in the creation networks here. Topological network features were calculated using the python package NetworkX [36]. Specifically, we calculate the degree, degree centrality, betweenness centrality and closeness centrality of residues (nodes) to which variants localise. The degree of a node is defined as the number of nodes it is connected to, and the fraction of connected nodes constitutes its degree centrality. Betweenness centrality $BC$ is defined as the number of pairwise shortest paths which pass through a node:

$$BC(u) = \sum_{s,t \in V} \frac{\sigma(s,t|u)}{\sigma(s,t)} \tag{1}$$

Here V is the set of nodes, $\sigma(s,t)$ is the total number of shortest paths, and $\sigma(s,t|v)$ is the number of shortest paths which pass through the node $u$.

Closeness centrality $CC$ is the reciprocal distance of the sum of the shortest paths from all other $n-1$ nodes to node $v$:

$$CC(u) = \frac{n-1}{\sum_{v=1}^{n-1} d(v,u)} \tag{2}$$

Where $d(v,u)$ is the distance of the shortest path between nodes $v$ and $u$.

## 4 Calculation of protein core density

The density of residue packing in protein cores may impact on their ability to accommodate missense variants. To investigate this, a non-redundant set of representative structures for the UniProt canonical proteome was collated. Here, structures from the ZoomVar database were mapped in order of identity. Additional structures were only added to represent a protein if at least 50 % of the residues they covered were not mapped to a structure with higher identity. Protein core density was only calculated for those proteins with a single mapped structure. This was to avoid problems associated with determining the core

density for multi-domain proteins. The number of C$\alpha$ contacts ($<$ 8 Å) for each core residue [Q(SASA) $<$ 0.15] was counted. The mean number of contacts for all core residues within a protein was used as a proxy for protein density, the assumption being that protein cores with greater density will have a higher number of C$\alpha$ contacts. Only protein cores which comprise of $>$ 4 residues were analysed.

# 5 Enrichment analysis of gene sets

## 5.1 Gene set enrichment

Gene enrichment analyses were performed using Gene Set Enrichment Analysis (GSEA) [16], as implemented in the R fgsea package [37]. At the full-length protein level only sets with n $\geq$ 25 were considered. Due to incomplete structural coverage of the proteome, enrichment calculation for protein regions was possible for a smaller number of proteins. For consistency, all pathways analysed at the whole protein level were also analysed at the protein regions.

For GSEA on expression, abundance, stability and half-life data, they were performed as detailed above and in the main text Materials and Methods section, but using these statistics as input to the algorithm:

| | *Metrics used for GSEA* |
|---|---|
| **thermal stability** | Tm - mean(Tm) |
| **abundance** | log(ppm + 1) - mean(log(ppm + 1)) |
| **expression** | log(median(RPKM) + 1) - mean(log(median(RPKM) + 1)) |
| **half life** | log(hours) - mean(log(hours)) |

Table A: Proteomics and transcriptomics-based metrics used as enrichment statistics for GSEA analysis.

## 5.2 CATH architecture enrichment analysis

Pfam domains [29] were assigned CATH domains [38] using the per-residue mapping of structures to domains, from both data sets, available from the SIFTs resource [39]. A minimum of 50 residues, which mapped to both a particular CATH domain and a particular Pfam domain were required to assign a Pfam domain to a CATH domain. This threshold was used in order to prevent spurious assignments. If a Pfam domain appeared to map to more than one CATH domain, the majority vote, from the residue level mapping, was used. Using these assignments Pfam domains were mapped to CATH architectures, guided by the CATH hierarchy, to create "domain sets" for each architecture.

Architecture enrichment analysis was performed as for gene set enrichment analysis, as described in the main text (Section E.1), however here the VESs calculated from full-length domain-types, as well as the constituent structural regions (for each Pfam domain type) were used as the enrichment statistics. Additionally, as we were interested in the enrichment of individual architectures only "domain" sets of size n $\geq$ 25 were considered at all levels.

# 6 Database implementation

All data, including per-residue mappings, were stored in the ZoomVar MySQL database [40]. A web interface and REST (Representational State Transfer) architecture was implemented, using the Django framework [41], to allow users to query the ZoomVar database. It is available at `fraternalilab.kcl.ac.uk/ZoomVar`. The database is designed for programmatic access to structurally annotate user-specified variants of interest.

# References


[1] Landrum MJ, Lee JM, Benson M, Brown G, Chao C, Chitipiralla S, et al. ClinVar: public archive of interpretations of clinically relevant variants. Nucleic acids research. 2016;44(D1):D862–8. doi:10.1093/nar/gkv1222.

[2] Forbes SA, Beare D, Gunasekaran P, Leung K, Bindal N, Boutselakis H, et al. COSMIC: exploring the world's knowledge of somatic mutations in human cancer. Nucleic acids research. 2015;43(Database issue):D805–11. doi:10.1093/nar/gku1075.

[3] Lek M, Karczewski KJ, Minikel EV, Samocha KE, Banks E, Fennell T, et al. Analysis of protein-coding genetic variation in 60,706 humans. Nature. 2016;536(7616):285–91. doi:10.1038/nature19057.

[4] Aken BL, Ayling S, Barrell D, Clarke L, Curwen V, Fairley S, et al. The Ensembl gene annotation system. Database : the journal of biological databases and curation. 2016;2016. doi:10.1093/database/baw093.

[5] McLaren W, Gil L, Hunt SE, Riat HS, Ritchie GRS, Thormann A, et al. The Ensembl Variant Effect Predictor. Genome biology. 2016;17(1):122. doi:10.1186/s13059-016-0974-4.

[6] Chung SS, Laddach A, Thomas NSB, Fraternali F. Short loop motif profiling of protein interaction networks in acute myeloid leukaemia. bioRxiv. 2018;doi:10.1101/306886.

[7] Orchard S, Ammari M, Aranda B, Breuza L, Briganti L, Broackes-Carter F, et al. The MIntAct project–IntAct as a common curation platform for 11 molecular interaction databases. Nucleic acids research. 2014;42(Database issue):D358–63. doi:10.1093/nar/gkt1115.

[8] Chatr-Aryamontri A, Oughtred R, Boucher L, Rust J, Chang C, Kolas NK, et al. The BioGRID interaction database: 2017 update. Nucleic acids research. 2017;45(D1):D369–D379. doi:10.1093/nar/gkw1102.

[9] Szklarczyk D, Franceschini A, Wyder S, Forslund K, Heller D, Huerta-Cepas J, et al. STRING v10: protein-protein interaction networks, integrated over the tree of life. Nucleic acids research. 2015;43(Database issue):D447–52. doi:10.1093/nar/gku1003.

[10] Xenarios I, Salwínski L, Duan XJ, Higney P, Kim SM, Eisenberg D. DIP, the Database of Interacting Proteins: a research tool for studying cellular networks of protein interactions. Nucleic acids research. 2002;30(1):303–5.

[11] Peri S, Navarro JD, Kristiansen TZ, Amanchy R, Surendranath V, Muthusamy B, et al. Human protein reference database as a discovery resource for proteomics. Nucleic acids research. 2004;32(Database issue):D497–501. doi:10.1093/nar/gkh070.

[12] Havugimana PC, Hart GT, Nepusz T, Yang H, Turinsky AL, Li Z, et al. A census of human soluble protein complexes. Cell. 2012;150(5):1068–81. doi:10.1016/j.cell.2012.08.011.

[13] Rolland T, Taşan M, Charloteaux B, Pevzner SJ, Zhong Q, Sahni N, et al. A proteome-scale map of the human interactome network. Cell. 2014;159(5):1212–1226. doi:10.1016/j.cell.2014.10.050.

[14] Huttlin EL, Ting L, Bruckner RJ, Gebreab F, Gygi MP, Szpyt J, et al. The BioPlex Network: A Systematic Exploration of the Human Interactome. Cell. 2015;162(2):425–440. doi:10.1016/j.cell.2015.06.043.

[15] Poux S, Arighi CN, Magrane M, Bateman A, Wei CH, Lu Z, et al. On expert curation and scalability: UniProtKB/Swiss-Prot as a case study. Bioinformatics. 2017;33(21):3454–3460. doi:10.1093/bioinformatics/btx439.

[16] Subramanian A, Tamayo P, Mootha VK, Mukherjee S, Ebert BL, Gillette MA, et al. Gene set enrichment analysis: a knowledge-based approach for interpreting genome-wide expression profiles. Proceedings of the National Academy of Sciences of the United States of America. 2005;102(43):15545–50. doi:10.1073/pnas.0506580102.

[17] Vogelstein B, Papadopoulos N, Velculescu VE, Zhou S, Diaz LA, Kinzler KW. Cancer genome landscapes. Science. 2013;339(6127):1546–58. doi:10.1126/science.1235122.

[18] Durinck S, Moreau Y, Kasprzyk A, Davis S, De Moor B, Brazma A, et al. BioMart and Bioconductor: a powerful link between biological databases and microarray data analysis. Bioinformatics. 2005;21:3439–3440.

[19] Durinck S, Spellman PT, Birney E, Huber W. Mapping identifiers for the integration of genomic datasets with the R/Bioconductor package biomaRt. Nature Protocols. 2009;4:1184–1191.

[20] Vaquerizas JM, Kummerfeld SK, Teichmann SA, Luscombe NM. A census of human transcription factors: function, expression and evolution. Nature reviews Genetics. 2009;10(4):252–63. doi:10.1038/nrg2538.

[21] Finn RD, Attwood TK, Babbitt PC, Bateman A, Bork P, Bridge AJ, et al. InterPro in 2017-beyond protein family and domain annotations. Nucleic acids research. 2017;45(D1):D190–D199. doi:10.1093/nar/gkw1107.

[22] Wishart DS, Feunang YD, Guo AC, Lo EJ, Marcu A, Grant JR, et al. DrugBank 5.0: a major update to the DrugBank database for 2018. Nucleic acids research. 2018;46(D1):D1074–D1082. doi:10.1093/nar/gkx1037.

[23] Wang M, Herrmann CJ, Simonovic M, Szklarczyk D, von Mering C. Version 4.0 of PaxDb: Protein abundance data, integrated across model organisms, tissues, and cell-lines. Proteomics. 2015;15(18):3163–8. doi:10.1002/pmic.201400441.

[24] Franken H, Mathieson T, Childs D, Sweetman GMA, Werner T, Tögel I, et al. Thermal proteome profiling for unbiased identification of direct and indirect drug targets using multiplexed quantitative mass spectrometry. Nature protocols. 2015;10(10):1567–93. doi:10.1038/nprot.2015.101.

[25] Mathieson T, Franken H, Kosinski J, Kurzawa N, Zinn N, Sweetman G, et al. Systematic analysis of protein turnover in primary cells. Nature communications. 2018;9(1):689. doi:10.1038/s41467-018-03106-1.

[26] GTEx Consortium. The Genotype-Tissue Expression (GTEx) project. Nature genetics. 2013;45(6):580–5. doi:10.1038/ng.2653.

[27] Berman H, Henrick K, Nakamura H. Announcing the worldwide Protein Data Bank. Nature structural biology. 2003;10(12):980. doi:10.1038/nsb1203-980.

[28] Altschul SF, Madden TL, Schäffer AA, Zhang J, Zhang Z, Miller W, et al. Gapped BLAST and PSI-BLAST: a new generation of protein database search programs. Nucleic acids research. 1997;25(17):3389–402.

[29] Finn RD, Coggill P, Eberhardt RY, Eddy SR, Mistry J, Mitchell AL, et al. The Pfam protein families database: towards a more sustainable future. Nucleic acids research. 2016;44(D1):D279–85. doi:10.1093/nar/gkv1344.

[30] Finn RD, Clements J, Eddy SR. HMMER web server: interactive sequence similarity searching. Nucleic acids research. 2011;39(Web Server issue):W29–37. doi:10.1093/nar/gkr367.

[31] Notredame C, Higgins DG, Heringa J. T-Coffee: A novel method for fast and accurate multiple sequence alignment. Journal of molecular biology. 2000;302(1):205–17. doi:10.1006/jmbi.2000.4042.

[32] Cavallo L, Kleinjung J, Fraternali F. POPS: A fast algorithm for solvent accessible surface areas at atomic and residue level. Nucleic acids research. 2003;31(13):3364–6.

[33] Myers EW, Miller W. Optimal alignments in linear space. Computer applications in the biosciences : CABIOS. 1988;4(1):11–7.

[34] Xue LC, Dobbs D, Honavar V. HomPPI: a class of sequence homology based protein-protein interface prediction methods. BMC bioinformatics. 2011;12:244. doi:10.1186/1471-2105-12-244.

[35] Bakan A, Meireles LM, Bahar I. ProDy: protein dynamics inferred from theory and experiments. Bioinformatics. 2011;27(11):1575–7. doi:10.1093/bioinformatics/btr168.

[36] Hagberg A, Swart P, S Chult D. Exploring network structure, dynamics, and function using NetworkX. Los Alamos National Lab.(LANL), Los Alamos, NM (United States); 2008.

[37] Sergushichev A. An algorithm for fast preranked gene set enrichment analysis using cumulative statistic calculation. bioRxiv. 2016;doi:10.1101/060012.

[38] Sillitoe I, Lewis TE, Cuff A, Das S, Ashford P, Dawson NL, et al. CATH: comprehensive structural and functional annotations for genome sequences. Nucleic acids research. 2015;43(Database issue):D376–81. doi:10.1093/nar/gku947.

[39] Velankar S, Dana JM, Jacobsen J, van Ginkel G, Gane PJ, Luo J, et al. SIFTS: Structure Integration with Function, Taxonomy and Sequences resource. Nucleic acids research. 2013;41(Database issue):D483–9. doi:10.1093/nar/gks1258.

[40] MySQL A. Mysql 5.1 reference manual; 2008. Available from: http://download.nust.na/pub6/mysql/doc/refman/5.1/en/index.html.

[41] Django;. http://djangoproject.com.
