# Supplementary material for: Pathogenic missense protein variants affect different functional pathways and proteomic features than healthy population variants
Source: PLoS Biol. 2021 Apr 28;19(4):e3001207. doi: 10.1371/journal.pbio.3001207 (PMC8110273; doi:10.1371/journal.pbio.3001207)
Supplement: S3 Text — (PDF) [file pbio.3001207.s003.pdf]

S3 Text

Supplementary Results

1 Population and disease-associated variants have different topological structural network properties

Here we ask whether variants from different datasets have distinct topological properties according to their structural localisation. We define these topological properties by representing protein structures at networks, in which nodes consist of Cα's, and those Cα's within 10 Å of one another are connected by edges (see Heading "Calculation of protein topological network features" in S2 Text for more details). Due to this representation, we are able to calculate topological network properties of the residues to which variants localise. Such properties give insight into the connectivity and neighbourhood of the affected residues.

The results of the analysis are depicted in Fig A. Given that we find protein cores to be enriched in ClinVar variants, it is perhaps unsurprising that we find these variants to localise to residues with a significantly higher degree (are more highly connected), than do variants from the other datasets (pairwise Mann-Whitney test, see S5 Data for values). We also find that disease-associated variants show significantly higher values for several other centrality measures. Interestingly, the most significant difference between datasets (Kruskal-Wallis test, see S5 Data), after degree, is betweenness centrality. This metric can be seen to highlight bottlenecks within the network, as it is a measure of the fraction of shortest paths which pass through a node. This suggests that disease-associated variants may target residues which play an important role in communication through the structural network. Conversely, ClinVar variants localise to residues with only slightly higher median degree centrality (the fraction of connected nodes) than population variants, and slightly lower median degree centrality than COSMIC cancer gene variants. This suggests that disease-associated variants localise to residues with a higher degree as they occur in proteins in which all residues are more highly connected. Although we see significant differences between the datasets, it is clear there is a large overlap in their distributions of topological network features (see Fig A).

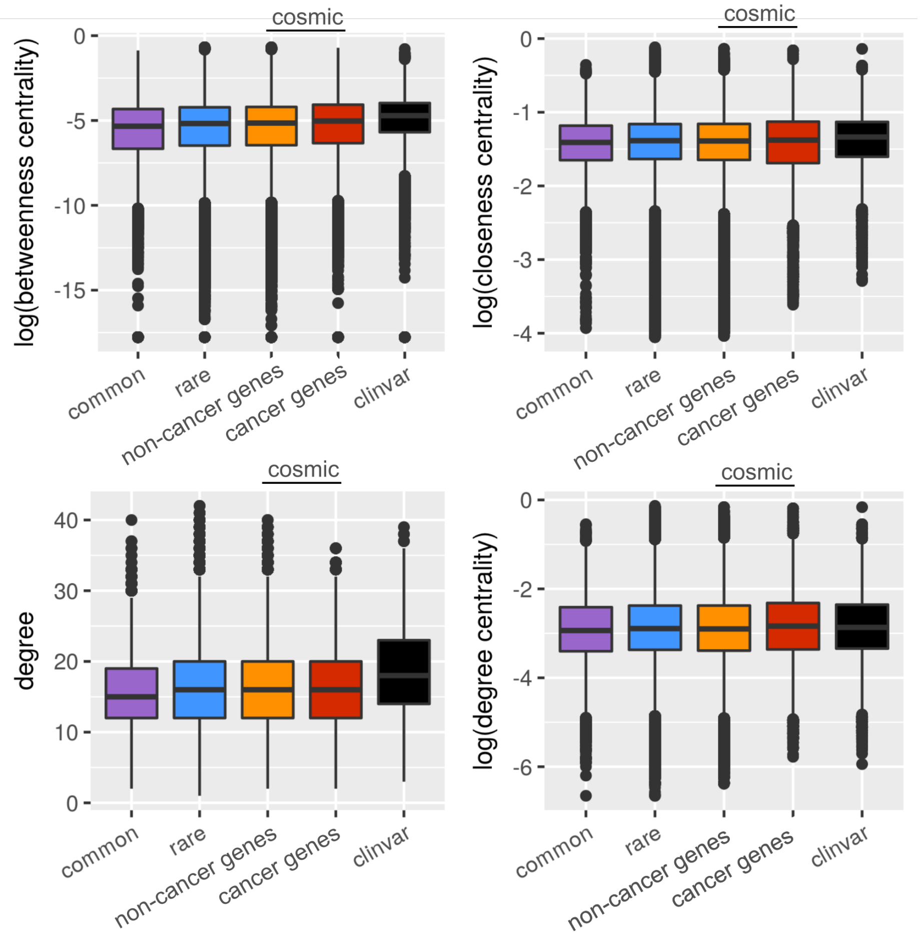

Fig A: Cα structural network topological features of variants. Proteins are transformed into networks based on positions of Cα carbons. Network topological features for the gnomAD common and rare, Cosmic and ClinVar datasets are compared. See S5 Data for underlying statistics.

2 Functional distinction of variant enrichment specific to protein structural regions

Across all pathways, the trends of functional distinction can be further visualised in the Circos [1] plot (Fig B), whereby the extent of shared enrichment of pathways is visualised by arcs across the different coloured segments. Identical to Fig 4 in the main text, pathways in the “response” cluster are enriched in missense variants across all four datasets; enrichment in the “proliferation” cluster is shown only in the disease-associated variants and the “nucleotide processing” cluster is unique to ClinVar variants. This analysis clearly distinguishes missense variants in health from those which are found in diseased individuals; moreover their different tendencies to perturb specific functional pathways are also highlighted.

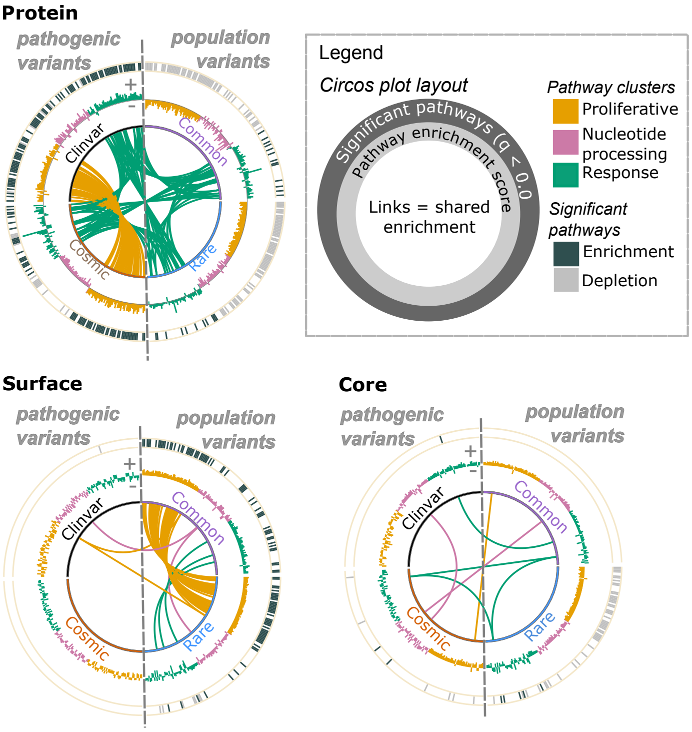

Fig B: Functional differences of proteins enriched in pathogenic and population missense variants. (*top*) Enrichment at the whole-protein level for each dataset visualised on a Circos plot (see legend). Pathogenic variants (here referring to ClinVar and COSMIC variants) are depicted on the left, and population variants (gnomAD rare and common) on the right, as indicated on the Circos plot. From the outer to inner layer of the plots, the following are depicted: (i) in the outermost layer of the plot, significant enrichment (dark grey) or depletion (light grey) of a pathway (q-value < 0.05) is depicted; (ii) in the middle layer of the plot, the normalised pathway enrichment score for each pathway is plotted as a bar graph (the further from the centre, the more positive; see '+' and '-' symbols on the plot); (iii) ; (iii) in the centre of the plot, links indicate enrichment (p-value < 0.05) shared between datasets. (*bottom*) Functional analysis of proteins according to variant enrichment at the protein surface and core, visualised on a Circos plot. Data are visualised in the same format as in the top panel. See S2 Data for underlying statistics.

We went on to extend this analysis to across protein structural regions (Fig B and Fig C). Here we find that proteins enriched in gnomAD variants at the surface (Fig B) are significantly enriched in pathways belonging to the "proliferation" cluster. Moreover, this enrichment is shared between common and rare variants (albeit not significant for common variants in individual pathways after false discovery rate [FDR] correction). Proteins with surfaces enriched in disease-associated variants (from COSMIC and ClinVar) are, contrastingly, not enriched in "proliferation" cluster pathways. Pathways in the "proliferation" cluster show either depletion (rare variants) or no patterns at all (common variants) for population variants, when the protein core (Fig B) and interface (Fig C) are concerned. This could indicate that population variants avoid the core of proliferation-related proteins. Interestingly, the "nucleotide processing" cluster does not show such a marked enrichment of variants which localise to the surface in the gnomAD dataset, a possible indication that proteins in these pathways are more robust to disruption of their structural fold, compared to those in the "proliferation" cluster. These data show that there is clearly an interplay between variant localisation at macroscopic (functional pathways) and microscopic (structural regions) protein features.

3 The localisation of variants to CATH domain architectures

We studied whether disease-associated missense variants would localise preferentially to domains with specific architectures. To accomplish this we made use of the CATH protein domain classification system [2] and focussed on the architectural level, which groups domains with similar secondary structural orientations, thereby capturing tertiary structural features. We mapped Pfam domain definitions to those used by CATH, and created domain sets (analogous to gene sets) for each CATH architecture. Enrichment was calculated at both the domain-type level (i.e. localisation of missense variants to a domain-type, for example fibronectin type-III (Fn3), in comparison to localisation of missense variants to all other domain types, see main text Fig 1), and at the domain-type region level (i.e. localisation of missense variants to core residues within a domain-type, for example all Fn3 core residues, in comparison to the localisation of missense variants to all other residues within a domain-type).

As depicted in Fig D, the results show that, at the whole domain level, the data sets show similar trends in variant localisation. A number of architectures, such as the Alpha Horseshoe architecture, show depletion of variants in all data sets, in contrast to other architectures, for example the Beta Sandwich and Irregular architectures, which show enrichment in all data sets. Few architectures, such as the Alpha-Beta Barrel, which is enriched in the gnomAD rare data but depleted for the other data sets, show markedly different patterns of enrichment at the domain architecture level. At the protein region level, the picture diversifies with the ClinVar data generally showing enrichment in architecture cores, although not significantly, in contrast to the other data sets which are more frequently depleted of variants in this region. Interestingly, this trend is particularly marked for the Alpha-Beta Barrel architecture. Thus, although gnomAD rare variants are enriched in this architecture, it is clear that very few

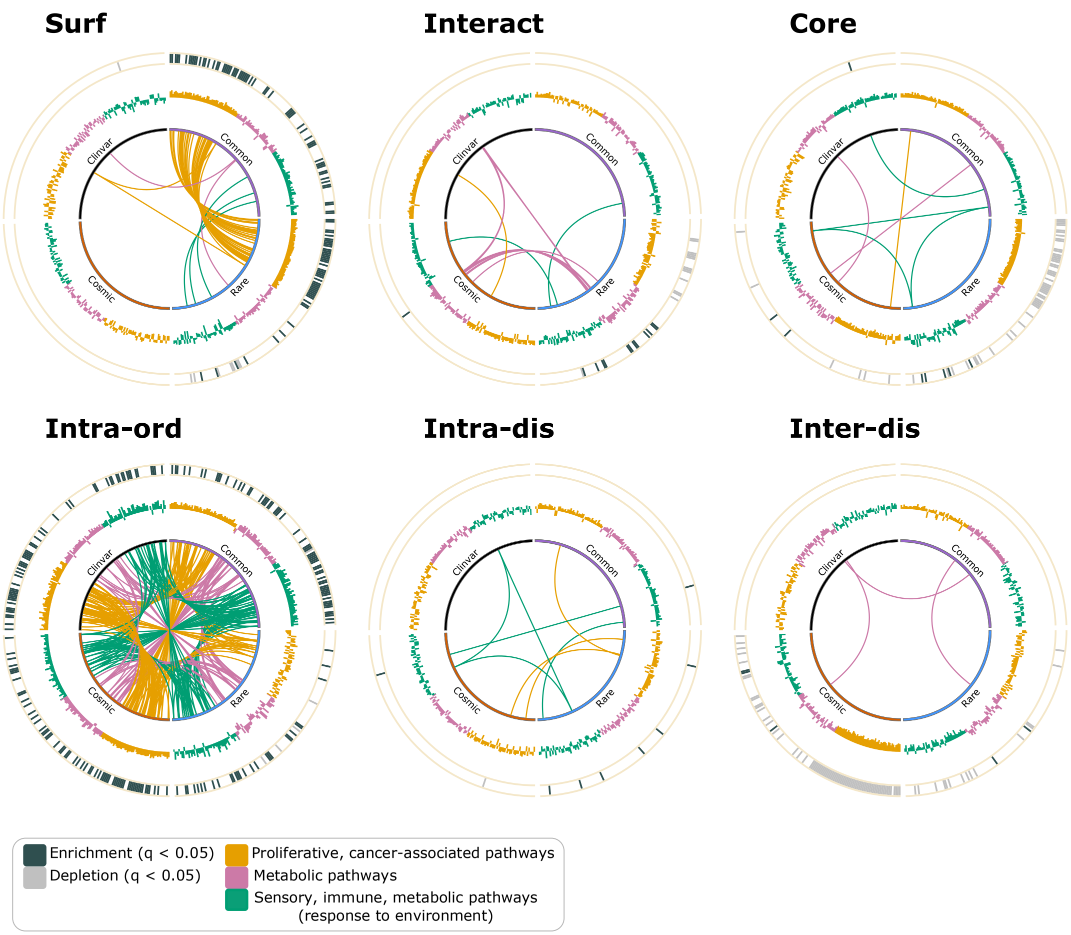

Fig C: Functional enrichment for each variant dataset, at different region levels, visualised on separate Circos plots. In each plot, the normalised enrichment score for each pathway is plotted as a bar graph (the further from the centre, the more positive) in the middle layer of the plot. In the outermost layer of the plot, significant enrichment (dark grey) or depletion (light grey) of a pathway ($q < 0.05$) is depicted. See S2 Data for underlying statistics.

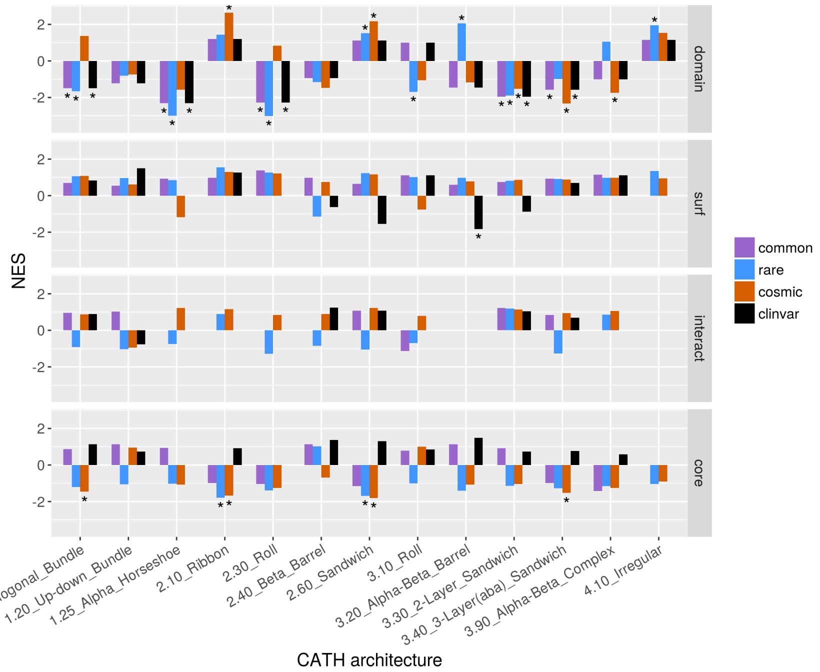

Fig D: The enrichment of CATH architectures in Pfam domains according to missense variant enrichment. Results are depicted at the domain level and the domain region level. * indicates q-value < 0.05. See S2 Data for underlying statistics.

of these localise to this architecture's core.

4 Case study - DNA-binding proteins

This case study focusses on patterns of variant enrichment observed for DNA-binding proteins: a wealth of analyses have established the fundamental role of structural properties in the interaction of these proteins with DNA [3, 4, 5]. We considered a list of DNA-binding domains (DBD) curated in the literature [6], and compared their whole-domain and region VESs. Fig E shows that DBDs vary considerably in their targeting by disease-associated and population variants. Domain-specific patterns can be seen, e.g. zf-H2C2_2 domains, which are numerous in zinc finger (ZNF) proteins, appear to be enriched in variants on the exposed surfaces, for both gnomAD and COSMIC variant sets. While a considerable number of domains do not have strongly depleted/enriched VES (small bubbles in Fig E), those which do tend to exhibit variant enrichment on the surface for gnomAD rare and COSMIC, and enrichment in core (e.g. P53, Homeobox) or interacting interfaces (e.g. Forkhead).

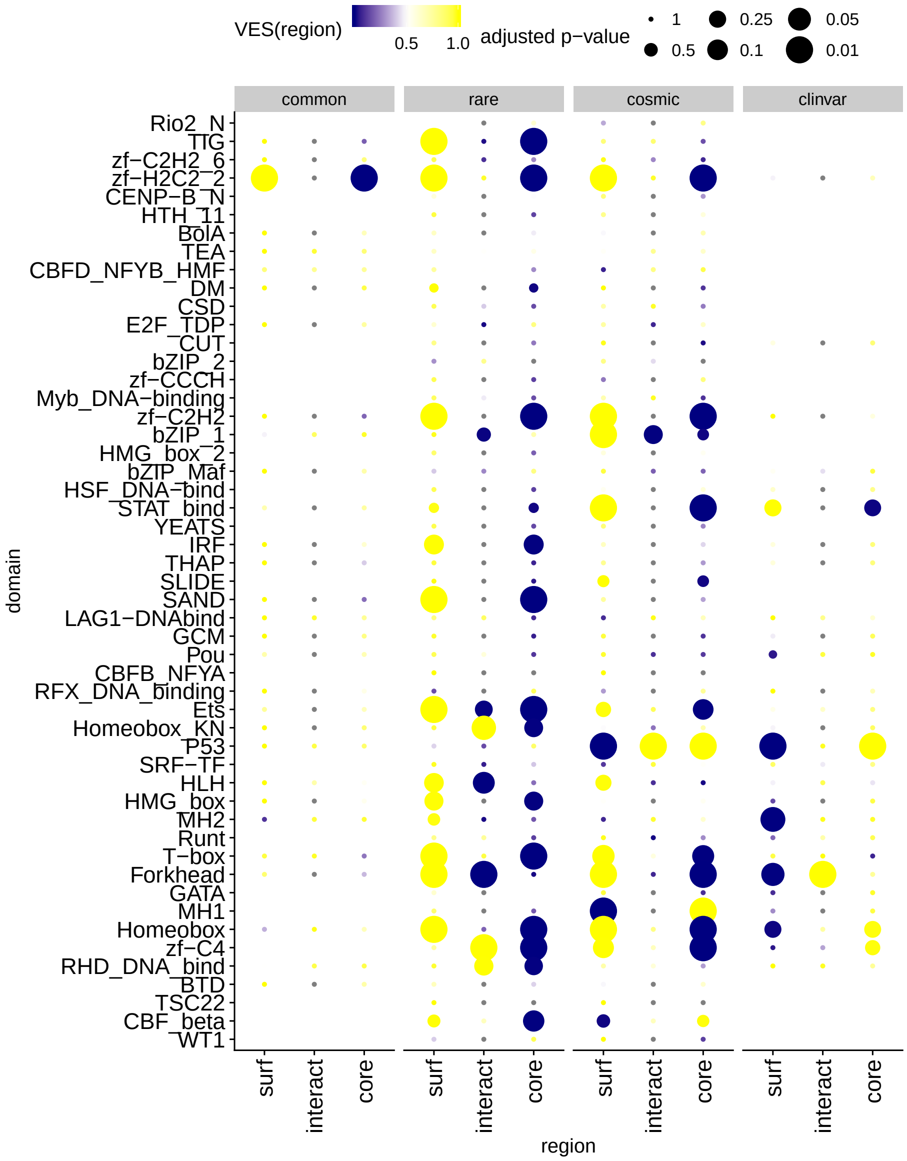

Fig E: Landscape of variant enrichment in DNA-binding domains (DBD). Here all DBDs, as curated in [6], with structural coverage are considered, and their domain-region level enrichment is depicted. Each row corresponds to a Pfam domain type. Region (surface, interface and core) enrichments are shown in the heat maps for variants from the gnomAD common, gnomAD rare, COSMIC and ClinVar datasets. See S2 Data for the underlying data.

5 Protein core density, stability and variant enrichment

One might expect that mutations would be less easily accommodated in cores of densely packed proteins, i.e. those likely to have higher thermal stability. To assess this we calculate the mean number of Cα contacts within 8 Å of core residues, as a proxy for protein density. We observe weak but significant correlation between this metric and protein thermal stability (Tm measurements from two replicates reported by Franken and colleagues [7]: replicate 1 [ρ = 0.168, q = 1.464e-12] and replicate 2 [ρ = 0.185, q = 1.529e-13]). This metric of core density (see Heading "Calculation of protein core density", S2 Text) is negatively correlated with the core Variant Enrichment Score for the gnomAD common dataset. No other datasets show significant correlations with core density, however a clear trend emerges in which correlations with the core density become progressively more positive in the order of gnomAD common, gnomAD rare, COSMIC cancer genes, COSMIC non-cancer genes and ClinVar (see S10 Fig). This suggests variants may be more disruptive if they localise to a densely packed protein core.

References

[1] Krzywinski M, Schein J, Birol I, Connors J, Gascoyne R, Horsman D, et al. Circos: an information aesthetic for comparative genomics. Genome research. 2009;19(9):1639–45. doi:10.1101/gr.092759.109.

[2] Sillitoe I, Lewis TE, Cuff A, Das S, Ashford P, Dawson NL, et al. CATH: comprehensive structural and functional annotations for genome sequences. Nucleic acids research. 2015;43(Database issue):D376–81. doi:10.1093/nar/gku947.

[3] Luscombe NM, Thornton JM. Protein-DNA interactions: amino acid conservation and the effects of mutations on binding specificity. Journal of molecular biology. 2002;320(5):991–1009.

[4] Rohs R, Jin X, West SM, Joshi R, Honig B, Mann RS. Origins of specificity in protein-DNA recognition. Annual review of biochemistry. 2010;79:233–69. doi:10.1146/annurev-biochem-060408-091030.

[5] Schneider B, Cernỳ J, Svozil D, Cech P, Gelly JC, de Brevern AG. Bioinformatic analysis of the protein/DNA interface. Nucleic acids research. 2014;42(5):3381–94. doi:10.1093/nar/gkt1273.

[6] Vaquerizas JM, Kummerfeld SK, Teichmann SA, Luscombe NM. A census of human transcription factors: function, expression and evolution. Nature reviews Genetics. 2009;10(4):252–63. doi:10.1038/nrg2538.

[7] Franken H, Mathieson T, Childs D, Sweetman GMA, Werner T, Tögel I, et al. Thermal proteome profiling for unbiased identification of direct and indirect drug targets using multiplexed quantitative mass spectrometry. Nature protocols. 2015;10(10):1567–93. doi:10.1038/nprot.2015.101.
