# Supplementary material for: Pathogenic missense protein variants affect different functional pathways and proteomic features than healthy population variants
Source: PLoS Biol. 2021 Apr 28;19(4):e3001207. doi: 10.1371/journal.pbio.3001207 (PMC8110273; doi:10.1371/journal.pbio.3001207)
Supplement: S1 Fig — (PDF) [file pbio.3001207.s004.pdf]

S1 Fig

Distribution of VES and associated p-values

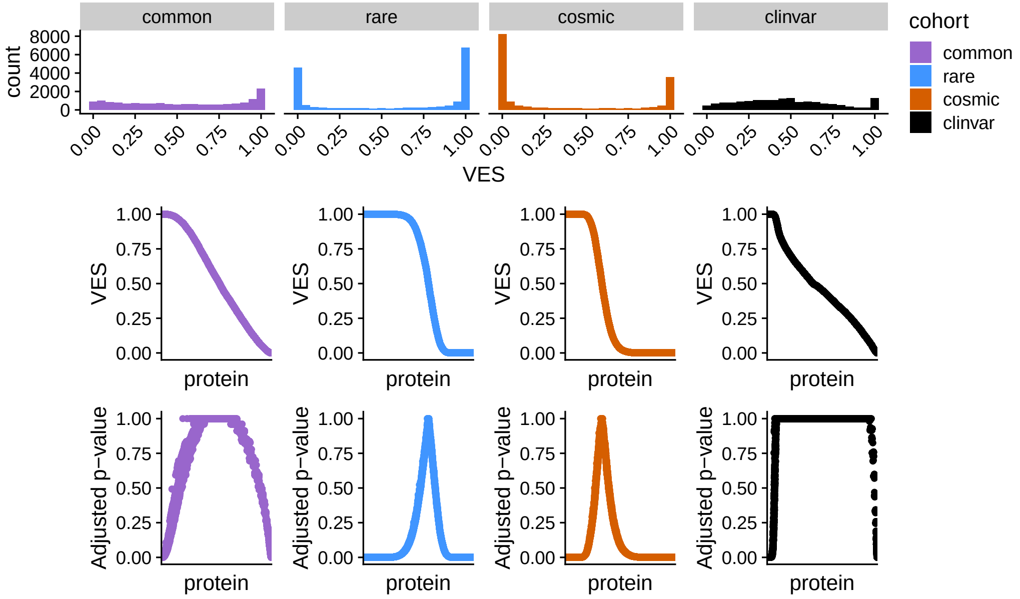

Distributions of VES and associated p-values calculated on individual proteins for gnomAD common, rare, COSMIC and ClinVar. (*top*) Distribution of whole-protein VES in the four datasets. (*bottom*) VES and associated p-values for each protein the four datasets. For each dataset, proteins are ranked by their VES, such that the horizontal axes for the VES and p-value scatterplots are identical to one another. See S2 Data for the underlying data.
