# Supplementary material for: Pathogenic missense protein variants affect different functional pathways and proteomic features than healthy population variants
Source: PLoS Biol. 2021 Apr 28;19(4):e3001207. doi: 10.1371/journal.pbio.3001207 (PMC8110273; doi:10.1371/journal.pbio.3001207)
Supplement: S2 Fig — (PDF) [file pbio.3001207.s005.pdf]

S2 Fig

Comparison of VES and density metrics

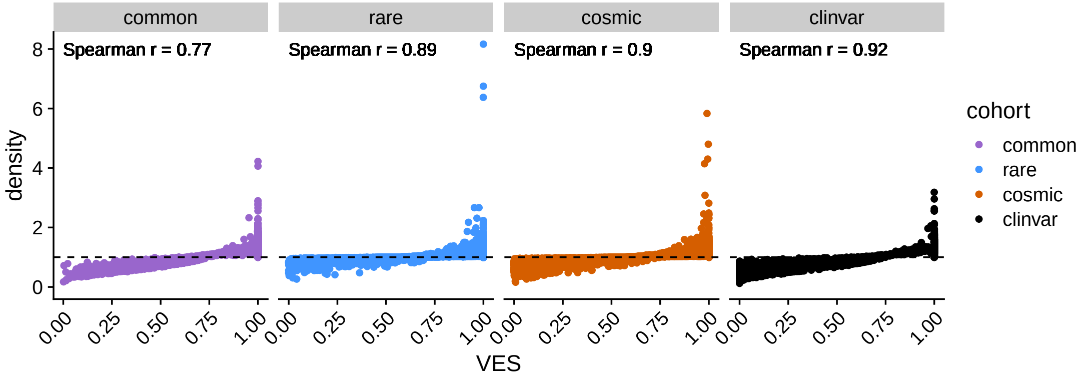

Comparison of VES and density metrics. Here we show, as an example, the variant enrichment statistics for surface regions of individual proteins in the four variant sets, where both VES and density metrics were calculated on the surface region of each protein. The Spearman correlation of the two statistics in each case is displayed. The dashed horizontal lines indicate variant density = 1 (i.e. the null case, with as much observed variants as expected). See S2 Data for the underlying data.
