# Supplementary material for: Pathogenic missense protein variants affect different functional pathways and proteomic features than healthy population variants
Source: PLoS Biol. 2021 Apr 28;19(4):e3001207. doi: 10.1371/journal.pbio.3001207 (PMC8110273; doi:10.1371/journal.pbio.3001207)
Supplement: S3 Fig — (PDF) [file pbio.3001207.s006.pdf]

S3 Fig

The density of mutations in different protein regions.

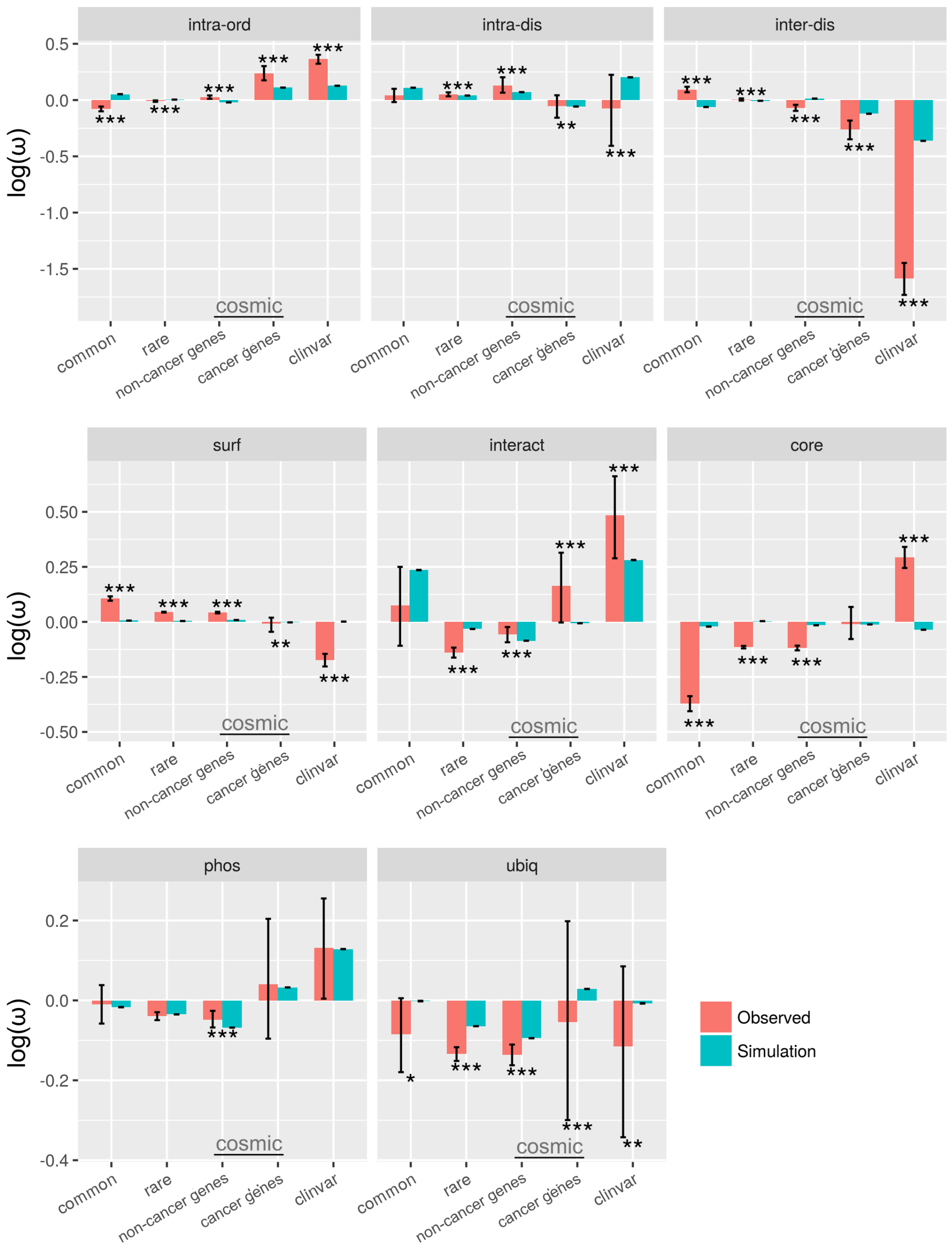

Density (ω) values were taken logarithm such that negative values indicate depletion while positive values indicate enrichment. Observed densities (pink), and densities derived from simulated null distributions (turquoise) are shown. Error bars depict 95% confidence intervals, for observed densities these were obtained by bootstrapping. Significance was calculated by comparison of observed values to simulated null missense variant distributions (significance level indicated by: * q-value < 0.05, ** q-value < 0.001, *** q-value < 0.0001). See S2 Data for the underlying data for the observed statistics. S3 Data contains statistics calculated for individual realisations of the simulated null distributions.
