# Supplementary material for: Pathogenic missense protein variants affect different functional pathways and proteomic features than healthy population variants
Source: PLoS Biol. 2021 Apr 28;19(4):e3001207. doi: 10.1371/journal.pbio.3001207 (PMC8110273; doi:10.1371/journal.pbio.3001207)
Supplement: S4 Fig — (PDF) [file pbio.3001207.s007.pdf]

S4 Fig

The landscape of variant enrichment over a list of domain-types most enriched in pathogenic and population variants.

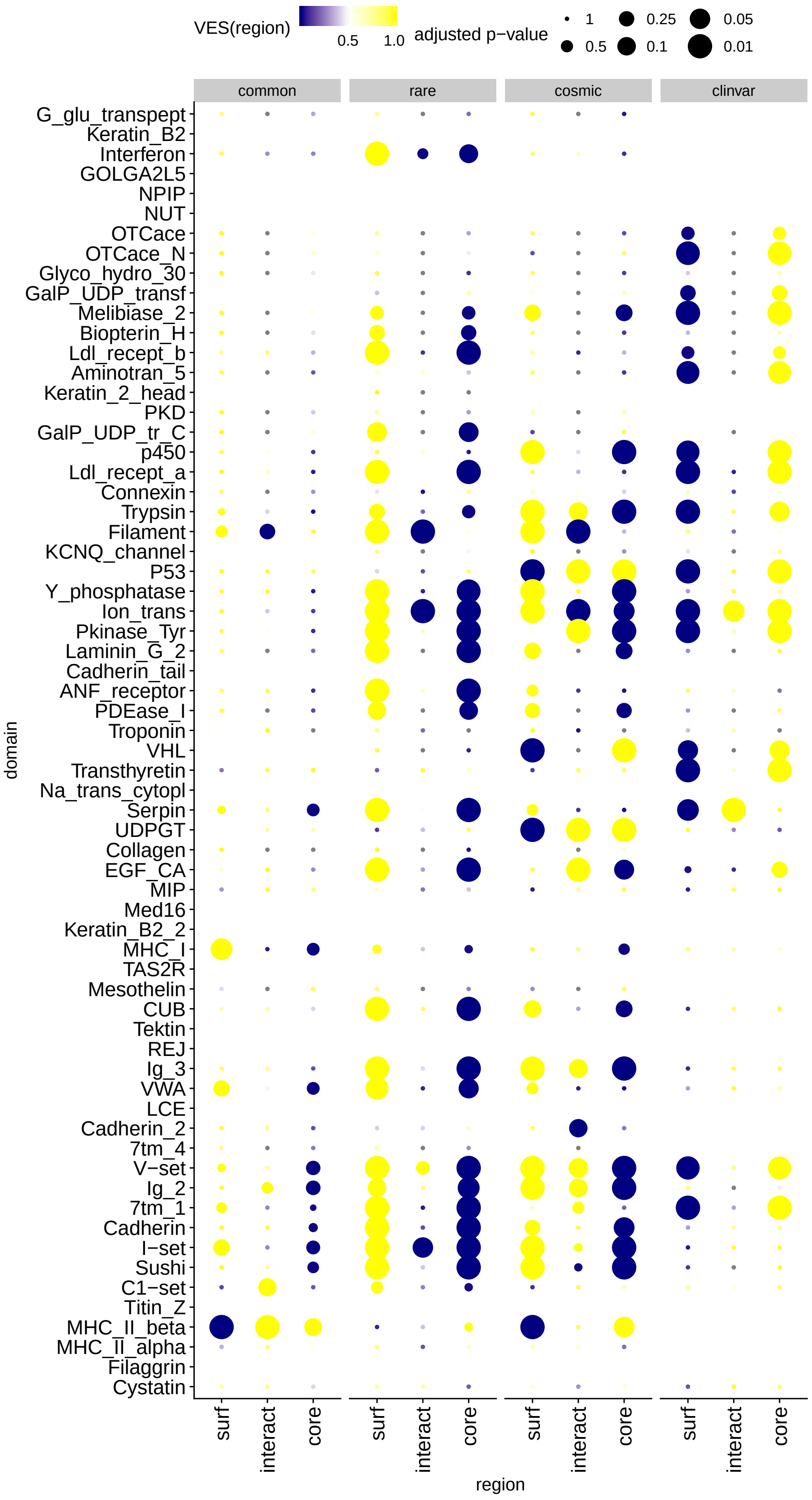

Here the union of the top 20 most enriched domain-types for each dataset is depicted: each role corresponds to a Pfam domain-type. Structural region (surface, interface and core) enrichments are shown in the heat maps for variants from the gnomAD common, gnomAD rare, COSMIC and ClinVar datasets. See S2 Data for the underlying data.
