# Supplementary material for: Pathogenic missense protein variants affect different functional pathways and proteomic features than healthy population variants
Source: PLoS Biol. 2021 Apr 28;19(4):e3001207. doi: 10.1371/journal.pbio.3001207 (PMC8110273; doi:10.1371/journal.pbio.3001207)
Supplement: S5 Fig — (PDF) [file pbio.3001207.s008.pdf]

S5 Fig

Principal Component Analysis (PCA) of pathway enrichment of protein-wise VES.

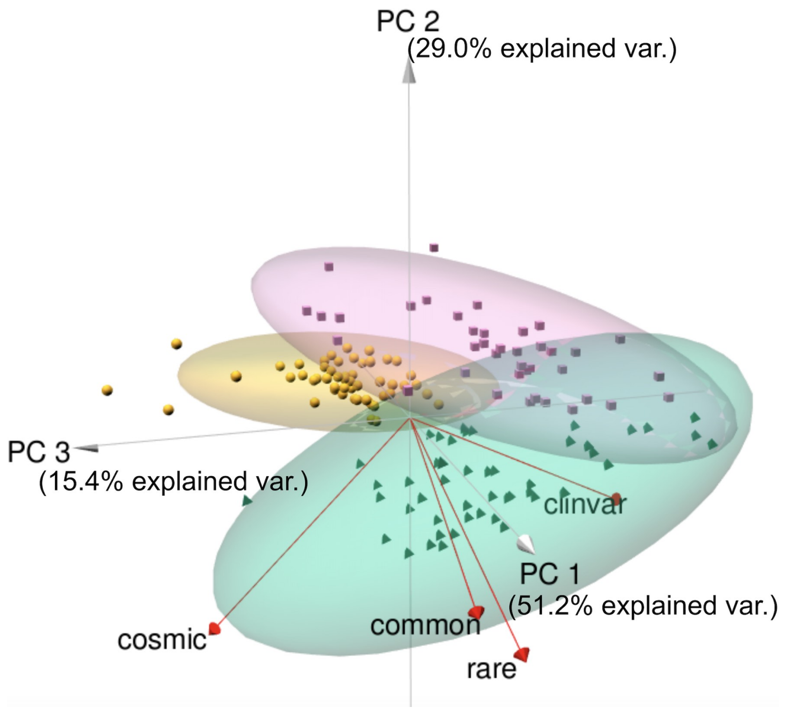

Data shown are identical to that of Fig 4B in the main text, but projected onto three dimensions. See S6 Data for the underlying data.
