# Supplementary material for: Pathogenic missense protein variants affect different functional pathways and proteomic features than healthy population variants
Source: PLoS Biol. 2021 Apr 28;19(4):e3001207. doi: 10.1371/journal.pbio.3001207 (PMC8110273; doi:10.1371/journal.pbio.3001207)
Supplement: S6 Fig — (PDF) [file pbio.3001207.s009.pdf]

S6 Fig

Stability and abundance of proteins enriched in variants in each dataset

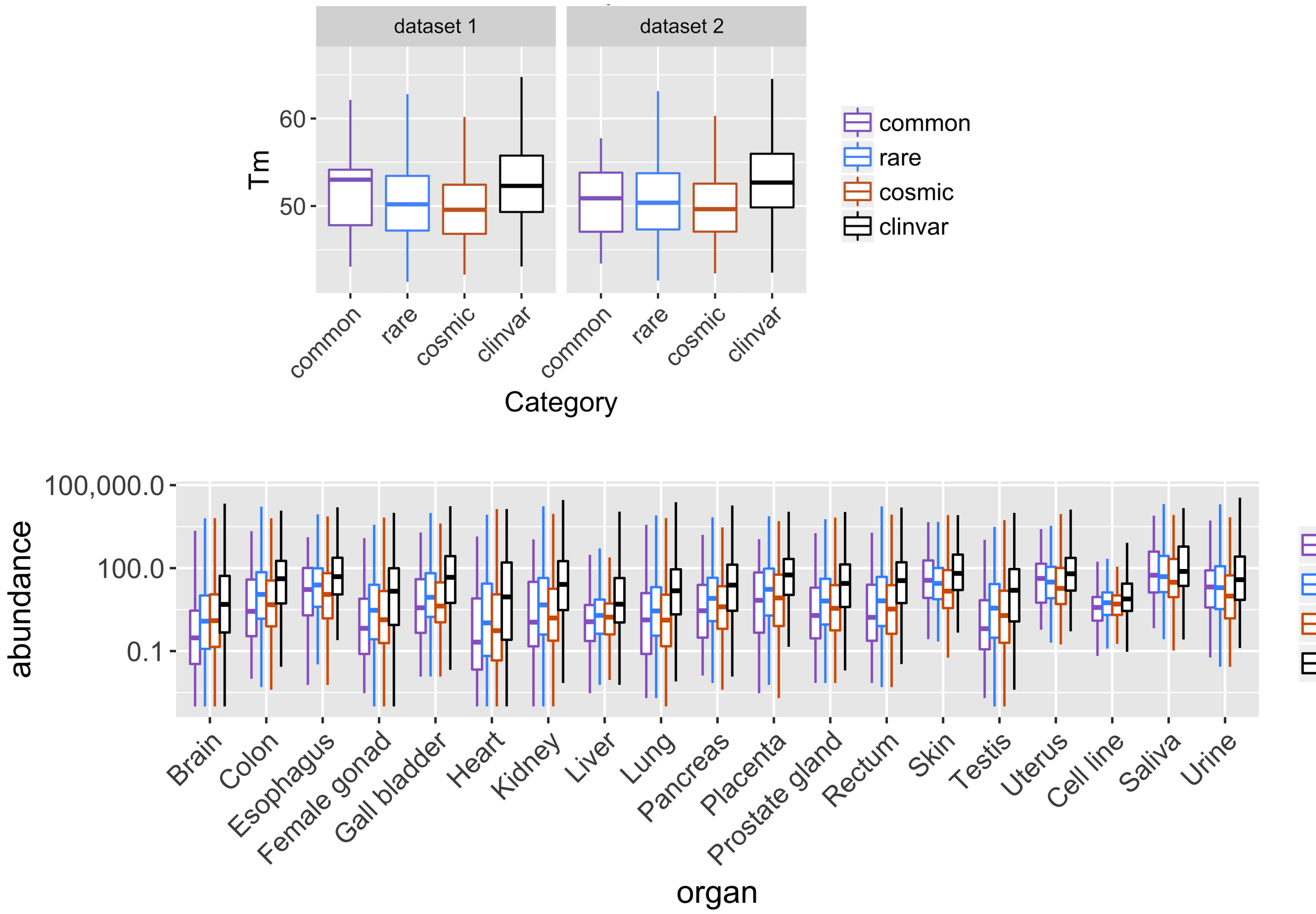

Stability and abundance of proteins enriched in variants in each dataset. For each dataset, here we considered the whole-protein level statistics and extracted proteins which were enriched (q-value < 0.05) in variants, and plotted distributions of their stability (melting temperature, or Tm [^{o}C], top panel) and abundance (ppm, bottom panel). Note these values correspond to the stability/abundance measurements of the wild-type protein, i.e. values at the 'base line' without any missense changes. Note again for the Tm data, measurements were done on two replicates ("dataset 1" and "dataset 2"). Both datasets were considered here and were shown in two separate panels (top). See S9 Data for the underlying data.
