# Supplementary material for: Pathogenic missense protein variants affect different functional pathways and proteomic features than healthy population variants
Source: PLoS Biol. 2021 Apr 28;19(4):e3001207. doi: 10.1371/journal.pbio.3001207 (PMC8110273; doi:10.1371/journal.pbio.3001207)
Supplement: S7 Fig — (PDF) [file pbio.3001207.s010.pdf]

S7 Fig

Spearman correlations for missense variant enrichment, protein stability and abundance

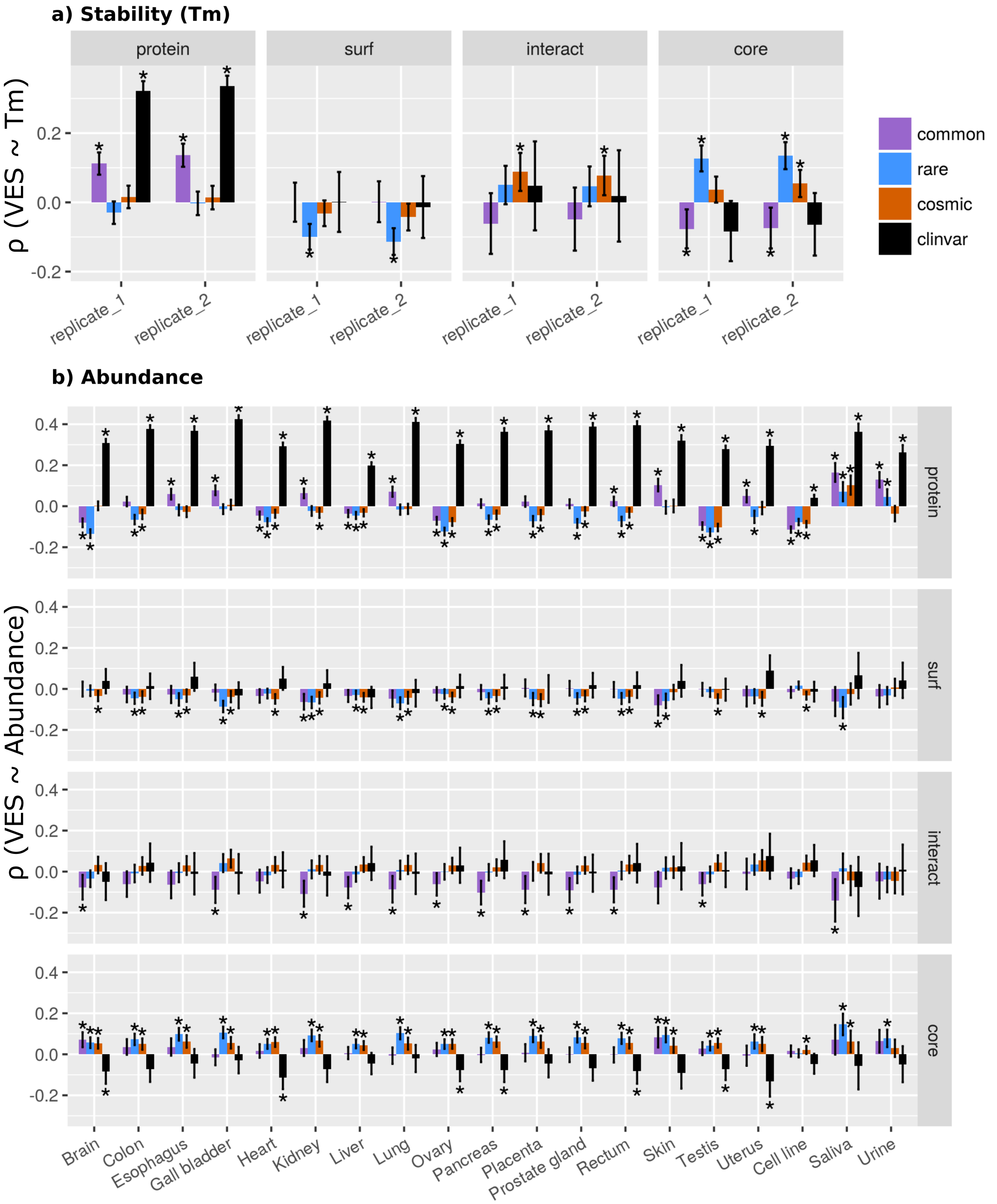

Spearman correlations for missense variant enrichment (quantified as VESs) with (a) protein thermal stability (Tm, in °C) and (b) protein abundance (ppm). Correlations with VESs calculated at the full-length "protein" level, and the surface (`surf`), core and interacting interface (`interact`). Error bars indicate 95% confidence intervals. * indicates q-value < 0.05. Selected organs/cell types are shown in the main text. See S9 Data for the underlying data.
