# Supplementary material for: Pathogenic missense protein variants affect different functional pathways and proteomic features than healthy population variants
Source: PLoS Biol. 2021 Apr 28;19(4):e3001207. doi: 10.1371/journal.pbio.3001207 (PMC8110273; doi:10.1371/journal.pbio.3001207)
Supplement: S8 Fig — (PDF) [file pbio.3001207.s011.pdf]

S8 Fig

The enrichment of missense variants in comparison to protein abundance and expression

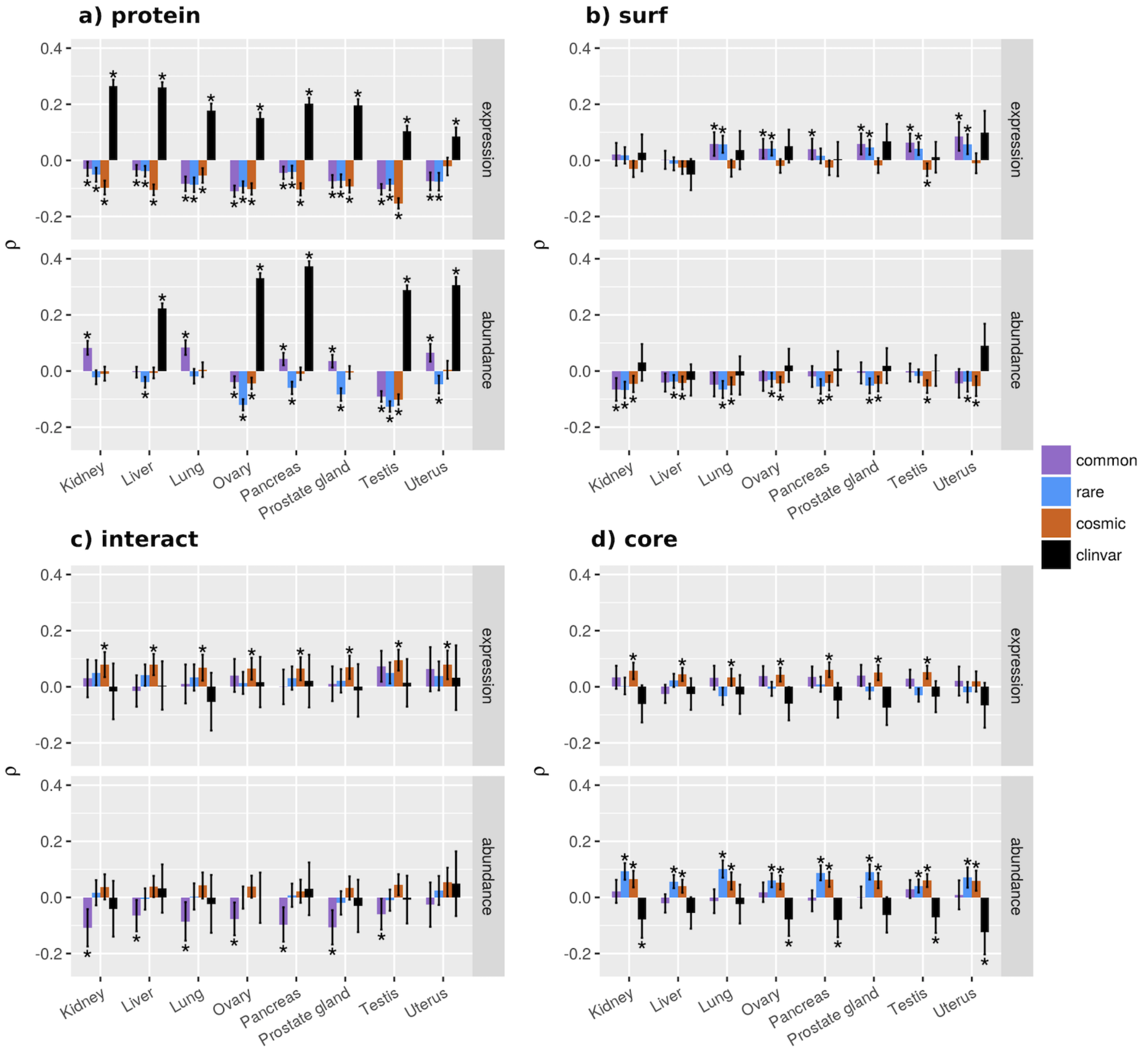

The enrichment of missense variants (VES) in comparison to protein abundance (ppm) and expression (median count) at the a) full-length protein level, b-d) surface (`surf`), core and interacting interface (`interact`). Spearman correlations calculated using only those proteins present in both abundance and expression data. Error bars indicate 95 % confidence intervals. * indicates q-value < 0.05. See S9 Data for the underlying data.
