# Supplementary material for: Pathogenic missense protein variants affect different functional pathways and proteomic features than healthy population variants
Source: PLoS Biol. 2021 Apr 28;19(4):e3001207. doi: 10.1371/journal.pbio.3001207 (PMC8110273; doi:10.1371/journal.pbio.3001207)
Supplement: S9 Fig — (PDF) [file pbio.3001207.s012.pdf]

S9 Fig

The Spearman correlation of the enrichment of missense variants with protein half lives

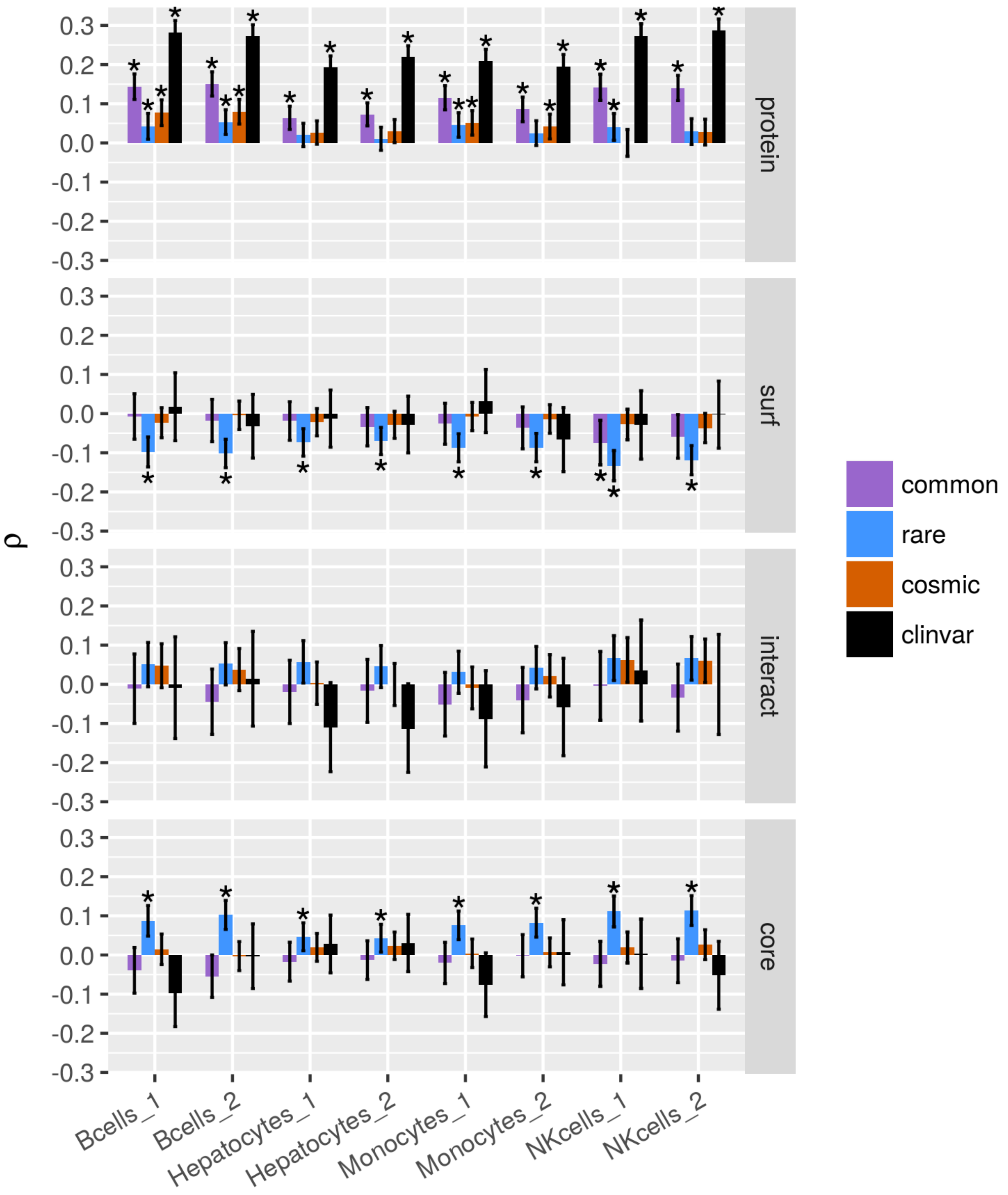

The Spearman correlation of the enrichment of missense variants (VES) with protein half life data (hours). Error bars indicate 95 % confidence intervals. * indicates q-value < 0.05. See S9 Data for the underlying data.
