# Supplementary material for: Pathogenic missense protein variants affect different functional pathways and proteomic features than healthy population variants
Source: PLoS Biol. 2021 Apr 28;19(4):e3001207. doi: 10.1371/journal.pbio.3001207 (PMC8110273; doi:10.1371/journal.pbio.3001207)
Supplement: S10 Fig — (PDF) [file pbio.3001207.s013.pdf]

S10 Fig

The Spearman correlation of the enrichment of missense variants in protein cores with protein core density

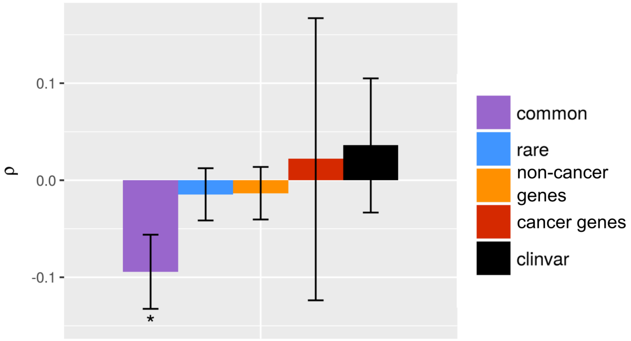

The Spearman correlation of the enrichment of missense variants in protein cores (VES) with a proxy for protein core density (see Methods). Error bars indicate 95 % confidence intervals. * indicates q-value < 0.05. See S2 Data for the underlying data.
