# Supplementary material for: Pathogenic missense protein variants affect different functional pathways and proteomic features than healthy population variants
Source: PLoS Biol. 2021 Apr 28;19(4):e3001207. doi: 10.1371/journal.pbio.3001207 (PMC8110273; doi:10.1371/journal.pbio.3001207)
Supplement: S11 Fig — (PDF) [file pbio.3001207.s014.pdf]

S11 Fig

Functional enrichment of proteins in KEGG pathways according to thermal stability.

The functional enrichment of proteins in KEGG pathways according to thermal stability. Pathways have been mapped to the 3 clusters defined in the main text. See S10 Data for the underlying data.

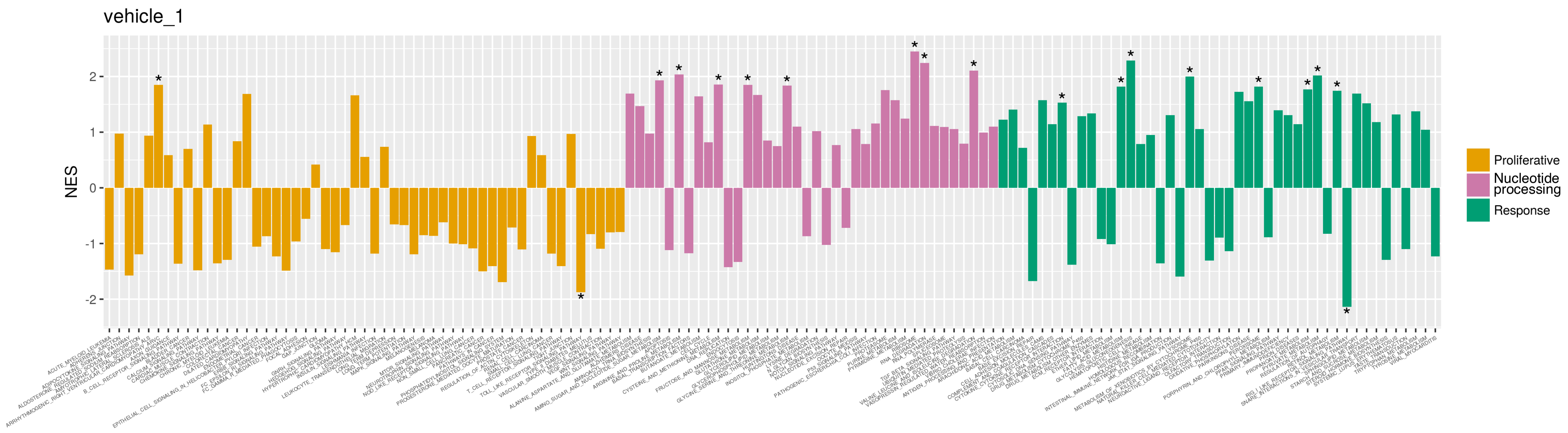
vehicle_1
NES
Proliferative
Nucleotide processing
Response

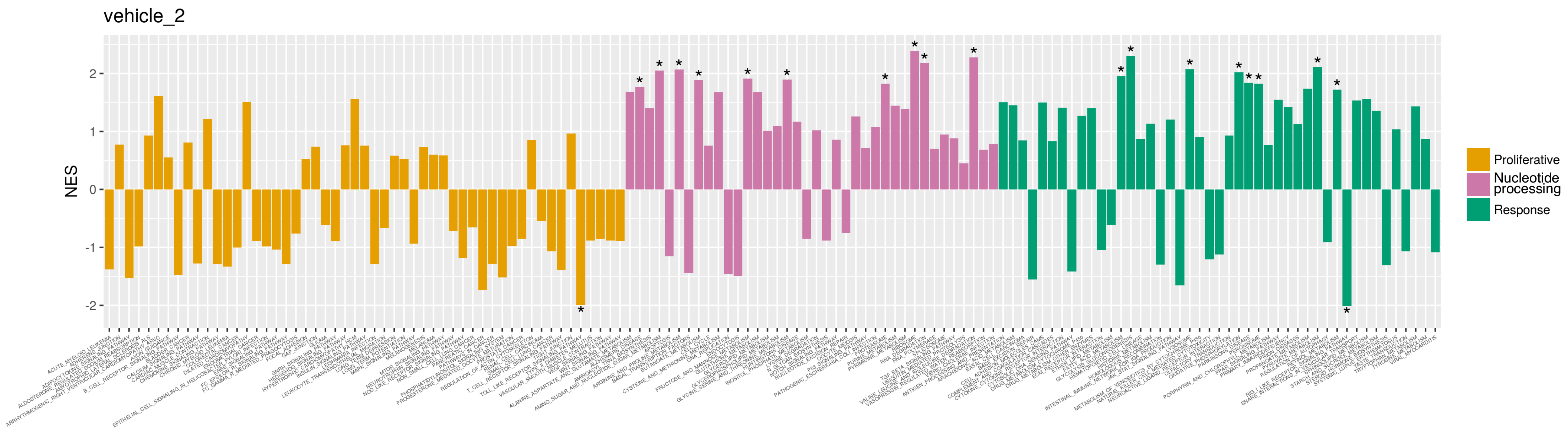
vehicle_2
NES
Proliferative
Nucleotide processing
Response
