# Supplementary material for: Pathogenic missense protein variants affect different functional pathways and proteomic features than healthy population variants
Source: PLoS Biol. 2021 Apr 28;19(4):e3001207. doi: 10.1371/journal.pbio.3001207 (PMC8110273; doi:10.1371/journal.pbio.3001207)
Supplement: S12 Fig — (PDF) [file pbio.3001207.s015.pdf]

S12 Fig

Functional enrichment of proteins in KEGG pathways according to protein abundance

The functional enrichment of proteins in KEGG pathways according to protein abundance. Pathways have been mapped to the 3 clusters defined in the main text. See S10 Data for the underlying data.

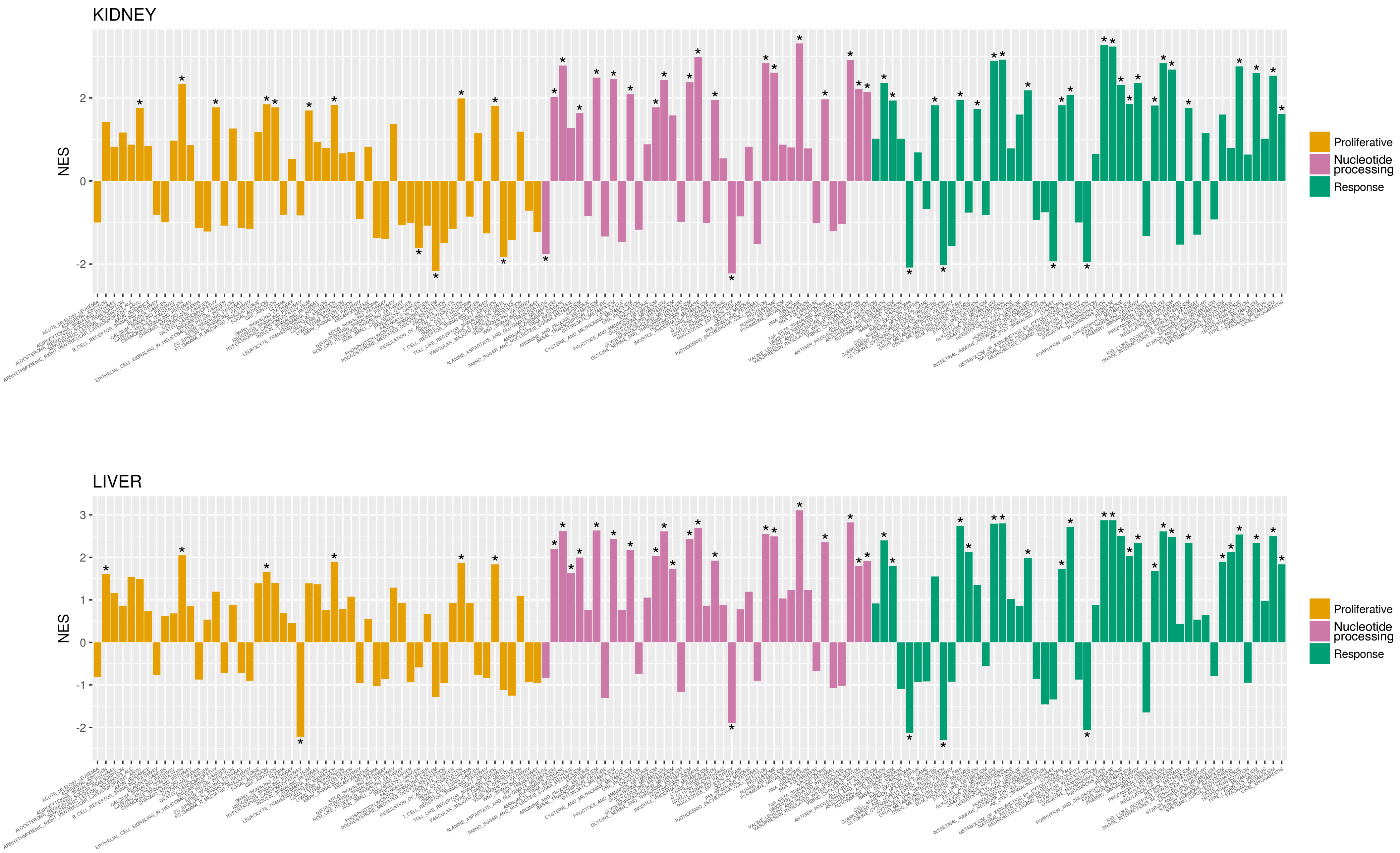
KIDNEY
NES
2
0
-2
LIVER
NES
3
2
1
0
-1
-2
Proliferative
Nucleotide processing
Response

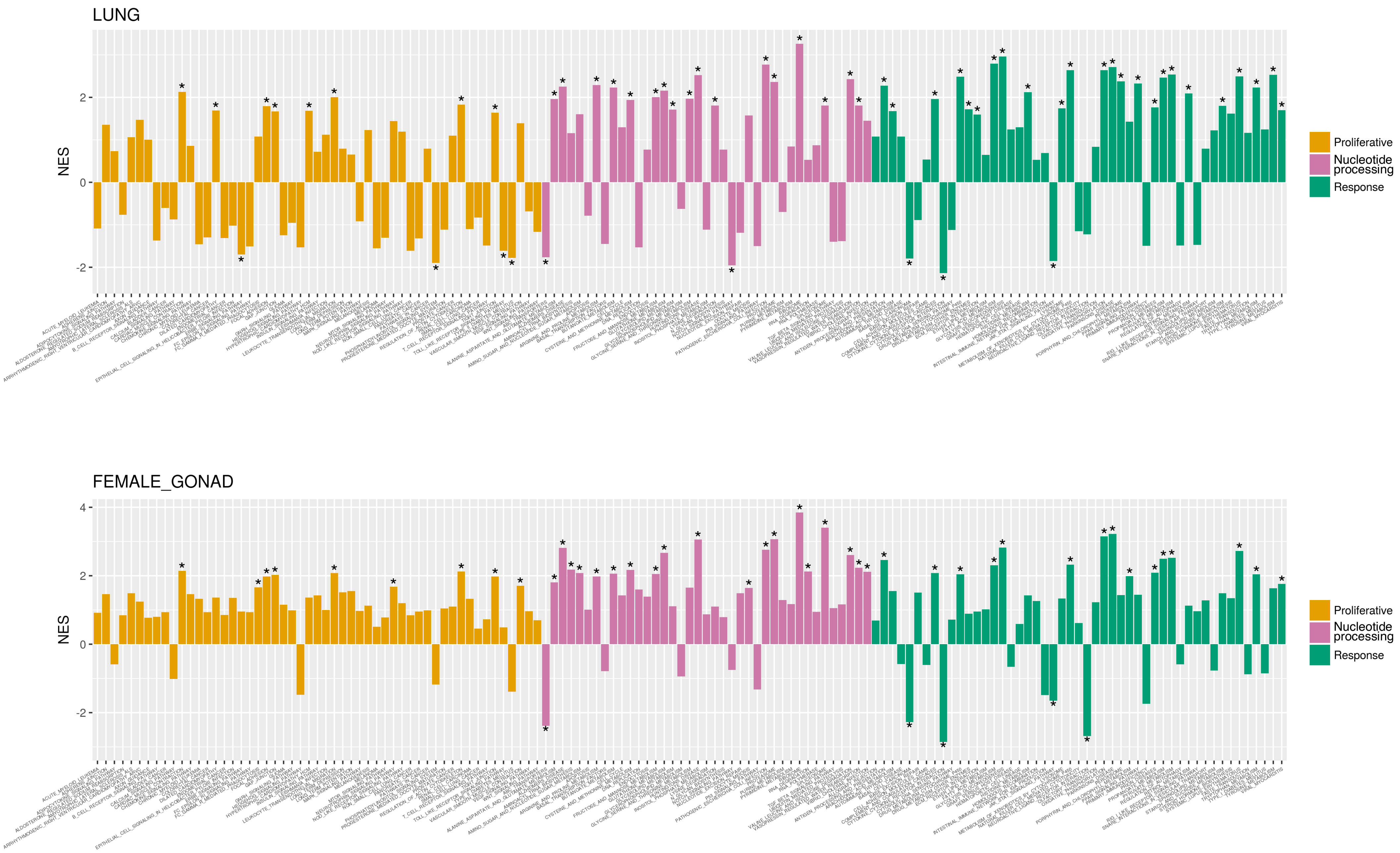
LUNG
NES
Proliferative
Nucleotide processing
Response
FEMALE_GONAD
NES
Proliferative
Nucleotide processing
Response

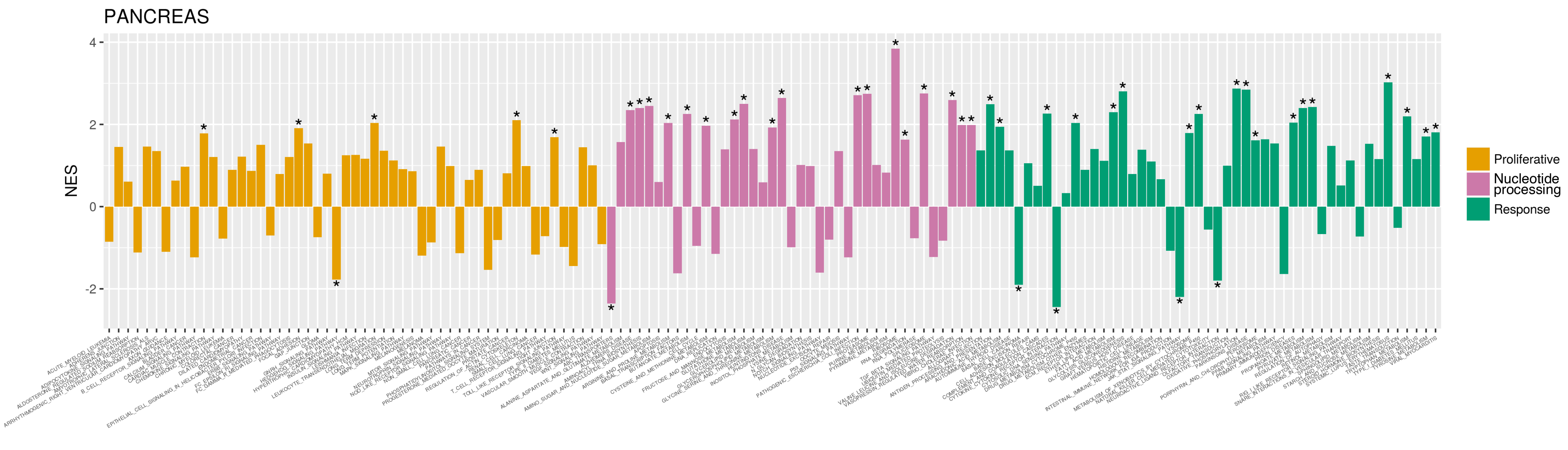
PANCREAS
NES
Proliferative
Nucleotide processing
Response

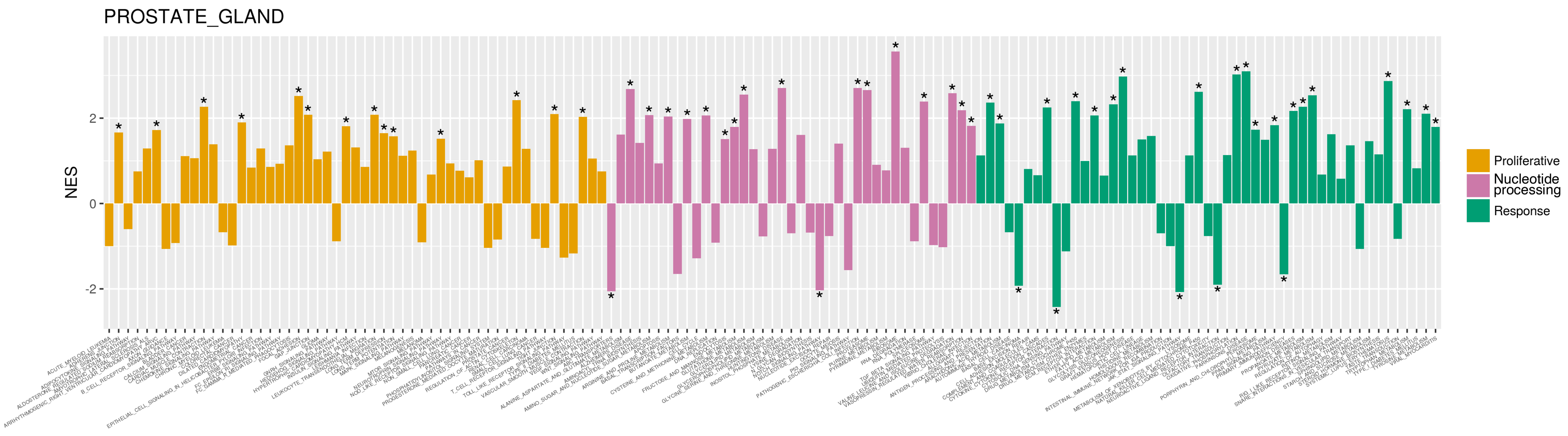
PROSTATE_GLAND
NES
Proliferative
Nucleotide processing
Response

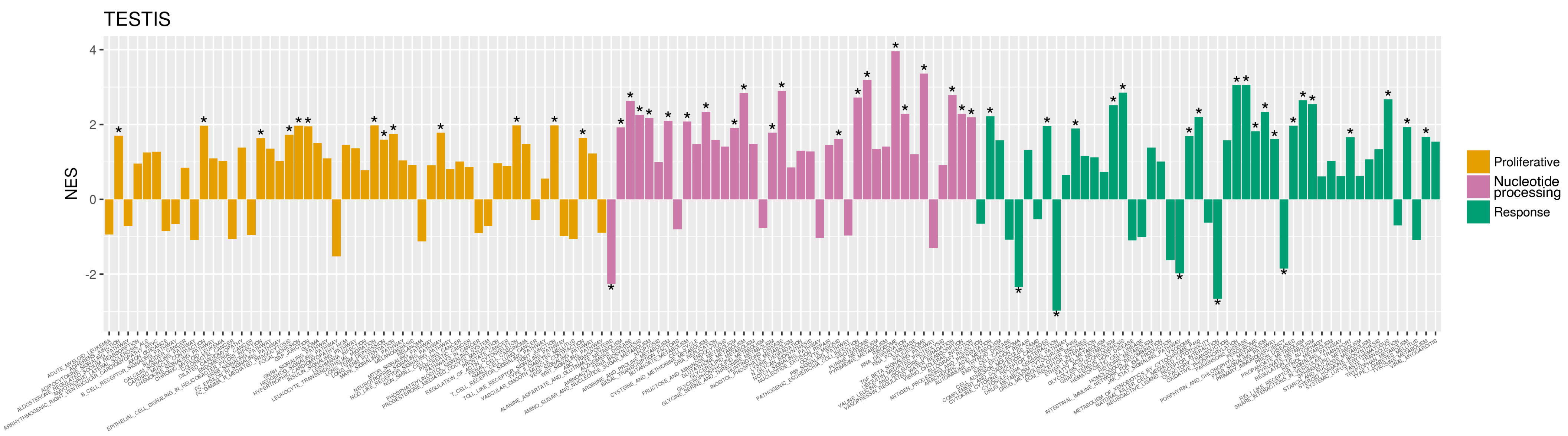
TESTIS
NES
4
2
0
-2
Proliferative
Nucleotide processing
Response

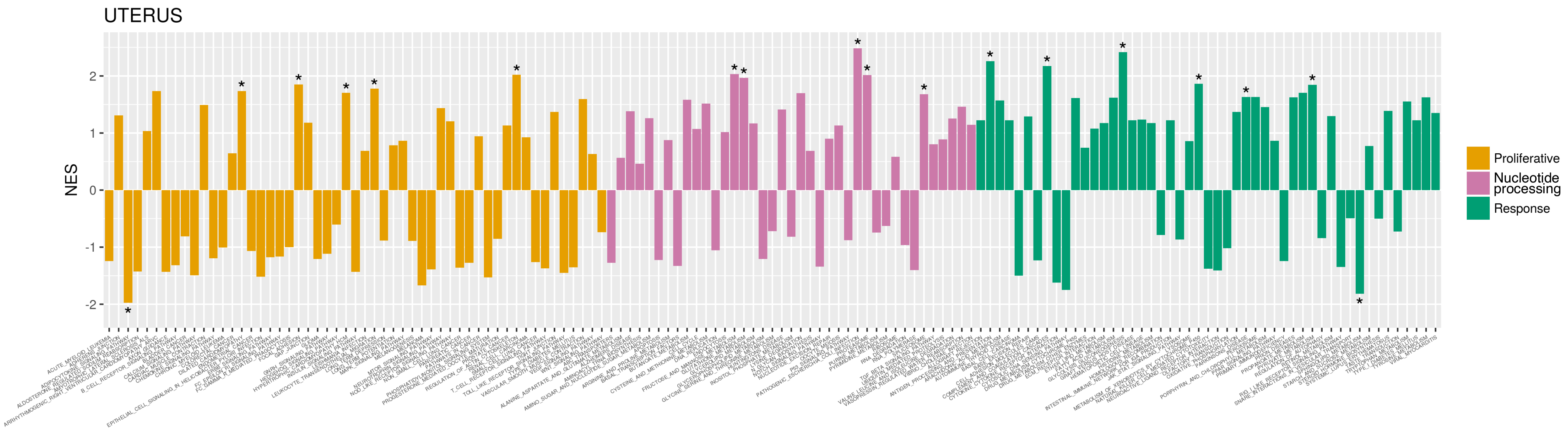
UTERUS
NES
2
1
0
-1
-2
Proliferative
Nucleotide processing
Response
