# Supplementary material for: Pathogenic missense protein variants affect different functional pathways and proteomic features than healthy population variants
Source: PLoS Biol. 2021 Apr 28;19(4):e3001207. doi: 10.1371/journal.pbio.3001207 (PMC8110273; doi:10.1371/journal.pbio.3001207)
Supplement: S13 Fig — (PDF) [file pbio.3001207.s016.pdf]

S13 Fig

Functional enrichment of proteins in KEGG pathways according to transcript expression

The functional enrichment of proteins in KEGG pathways according to transcript expression. Pathways have been mapped to the 3 clusters defined in the main text. See S10 Data for the underlying data.

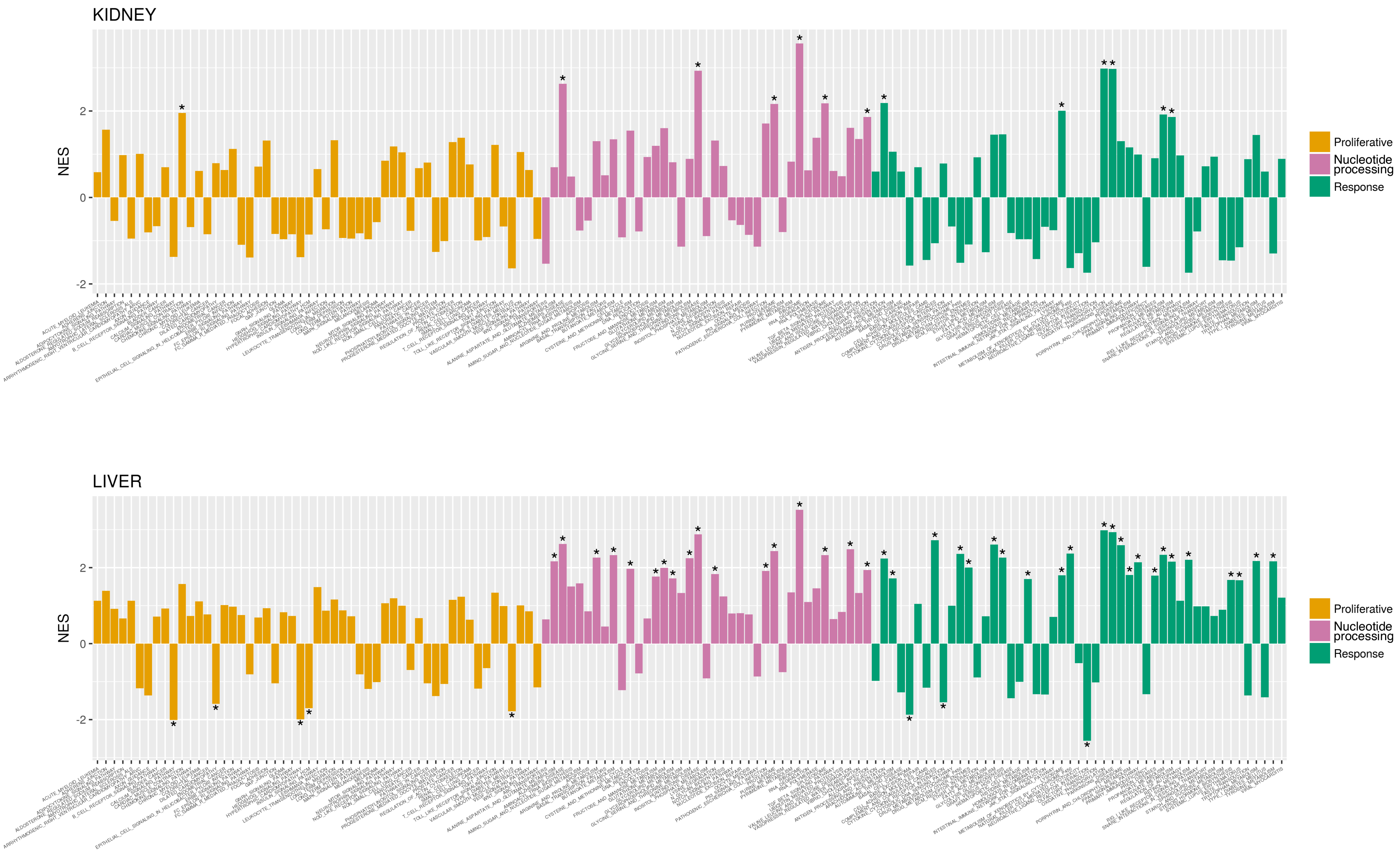
KIDNEY
NES
2
0
-2
Proliferative
Nucleotide processing
Response
LIVER
NES
2
0
-2
Proliferative
Nucleotide processing
Response

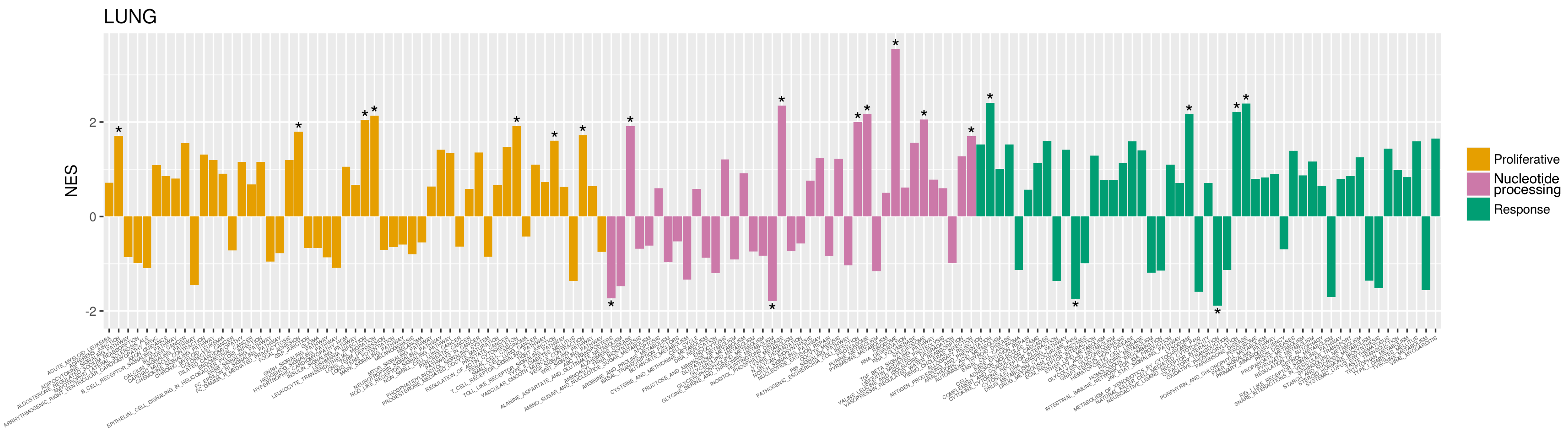
LUNG
NES
2
0
-2
Proliferative
Nucleotide processing
Response

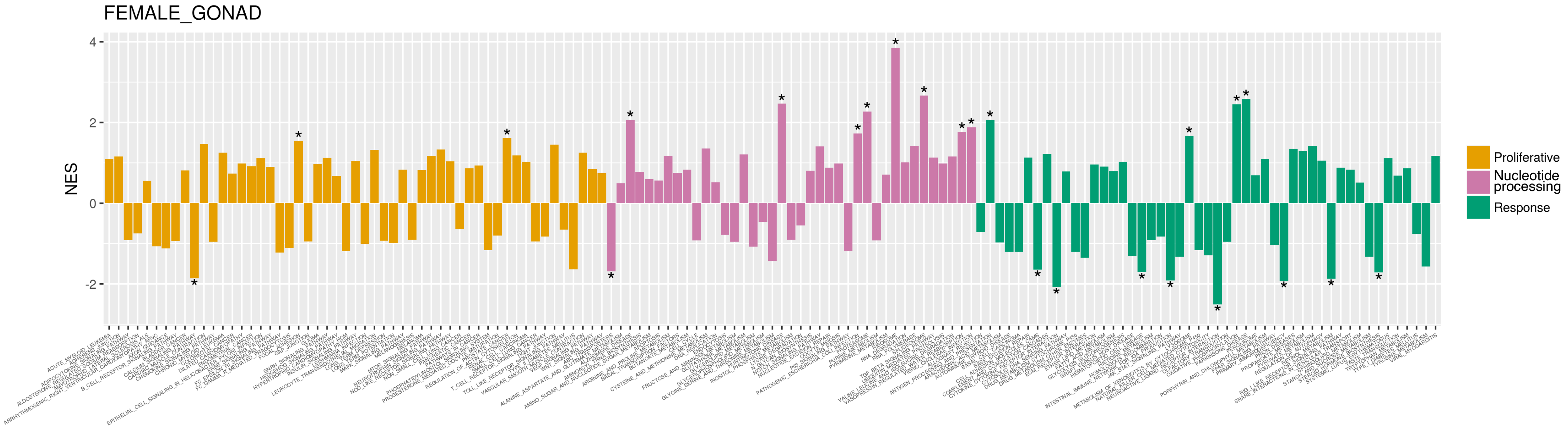
FEMALE_GONAD
NES
4
2
0
-2
Proliferative
Nucleotide processing
Response

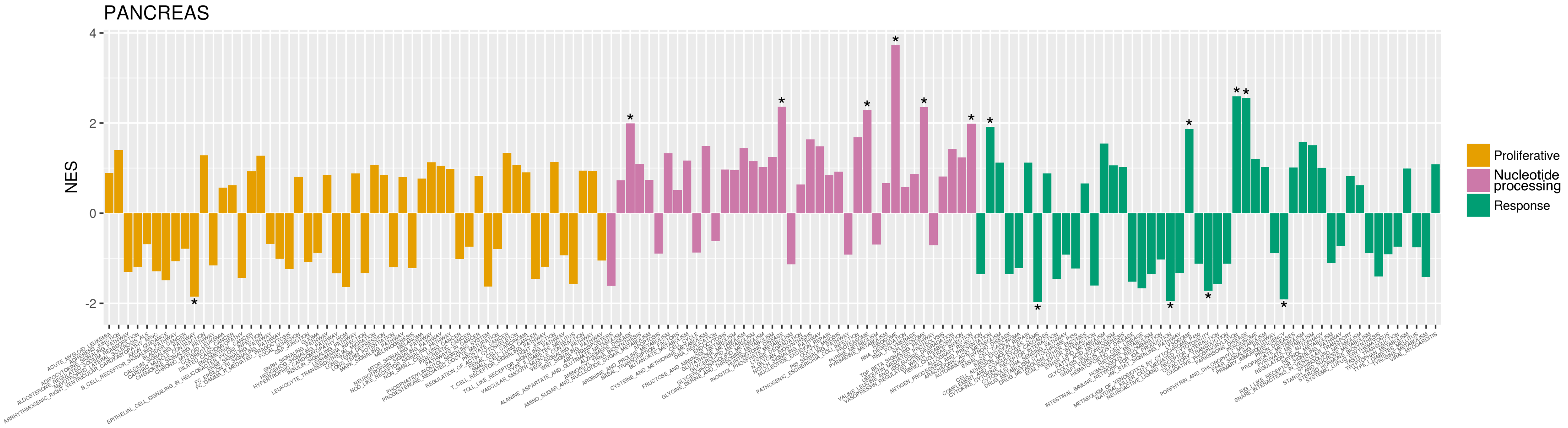
PANCREAS
NES
4
2
0
-2
Proliferative
Nucleotide processing
Response

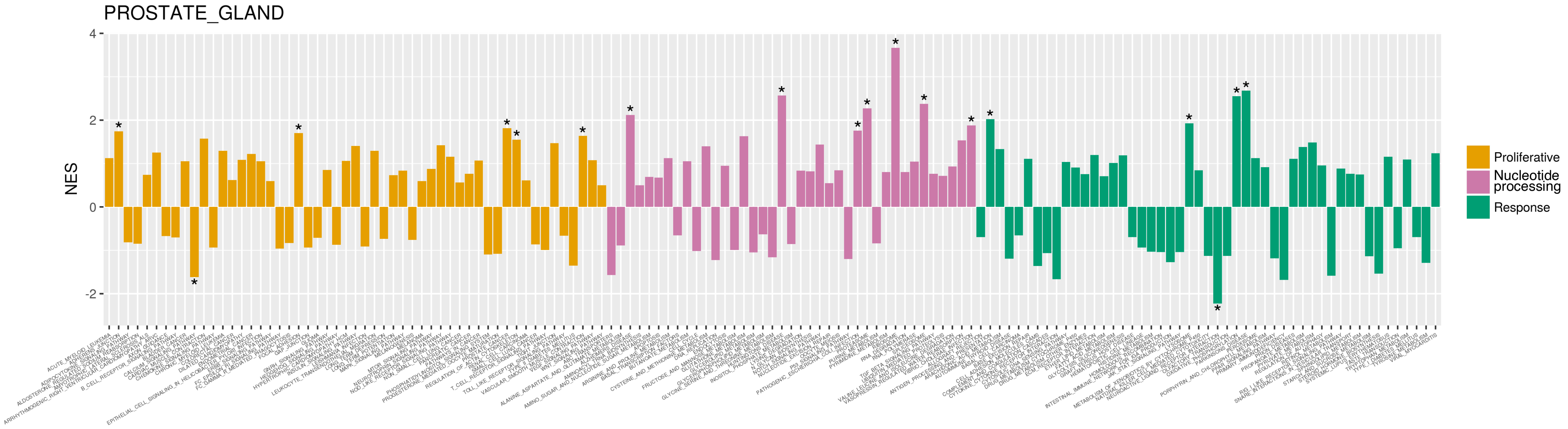
PROSTATE_GLAND
NES
4
2
0
-2
Proliferative
Nucleotide processing
Response

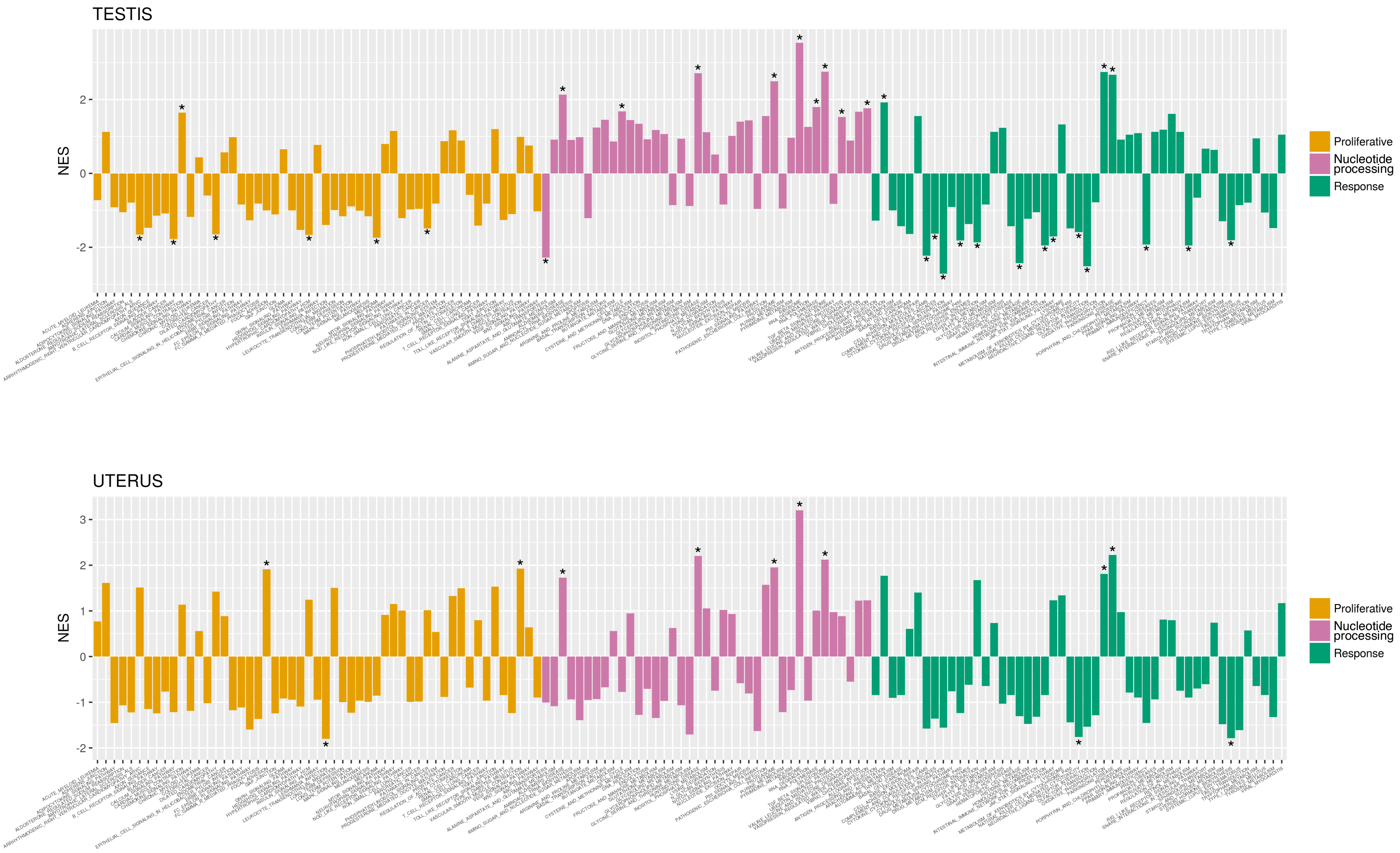
TESTIS
NES
Proliferative
Nucleotide processing
Response
UTERUS
NES
Proliferative
Nucleotide processing
Response
