# Supplementary material for: Pathogenic missense protein variants affect different functional pathways and proteomic features than healthy population variants
Source: PLoS Biol. 2021 Apr 28;19(4):e3001207. doi: 10.1371/journal.pbio.3001207 (PMC8110273; doi:10.1371/journal.pbio.3001207)
Supplement: S14 Fig — (PDF) [file pbio.3001207.s017.pdf]

S14 Fig

Functional enrichment of proteins in KEGG pathways according to protein half life

The functional enrichment of proteins in KEGG pathways according to protein half life. Pathways have been mapped to the 3 clusters defined in the main text. See S10 Data for the underlying data.

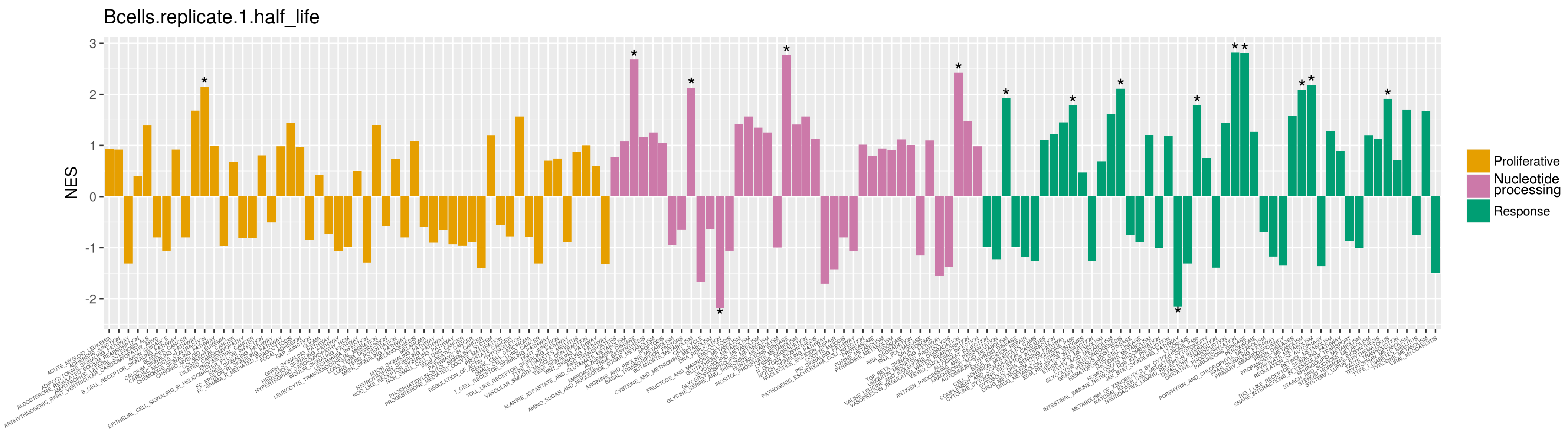
Bcells.replicate.1.half_life
NES
Proliferative
Nucleotide processing
Response

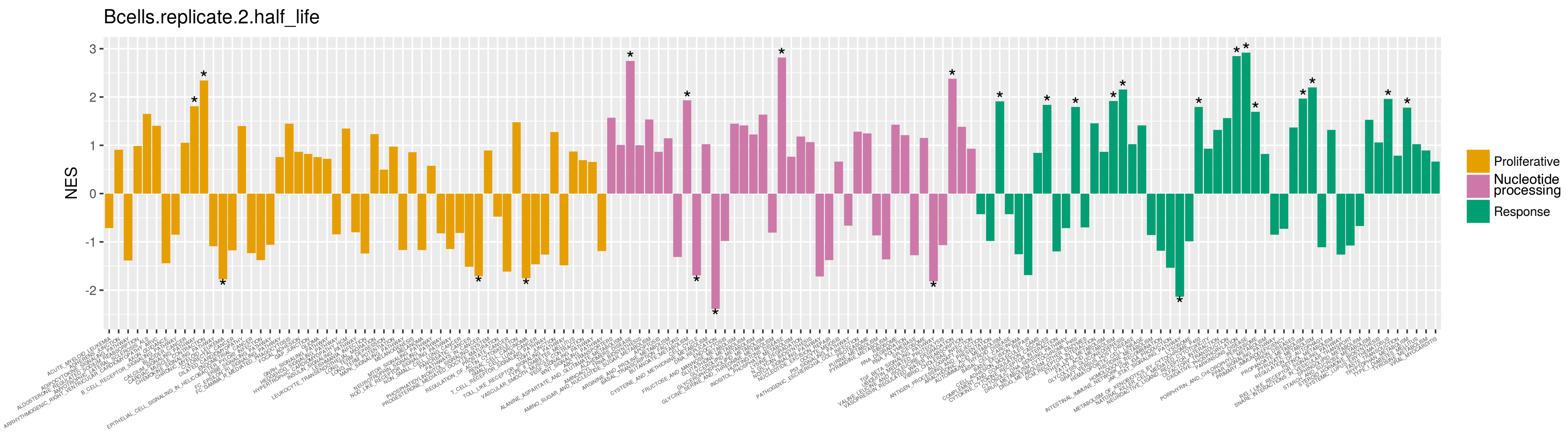
Bcells.replicate.2.half_life
NES
Proliferative
Nucleotide processing
Response

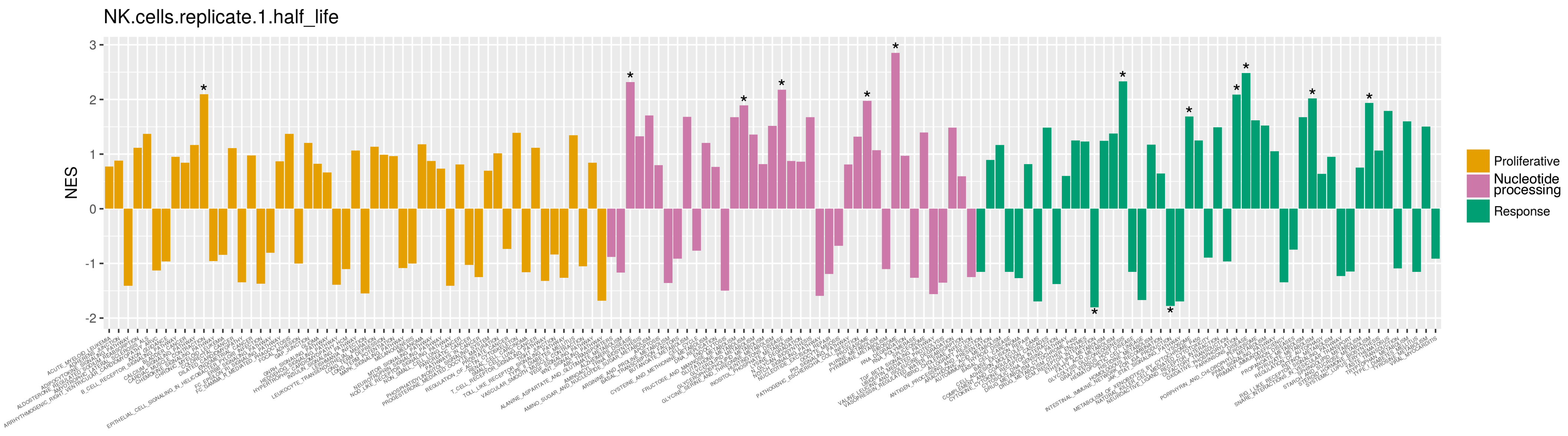
NK.cells.replicate.1.half_life
NES
3
2
1
0
-1
-2
Proliferative
Nucleotide processing
Response

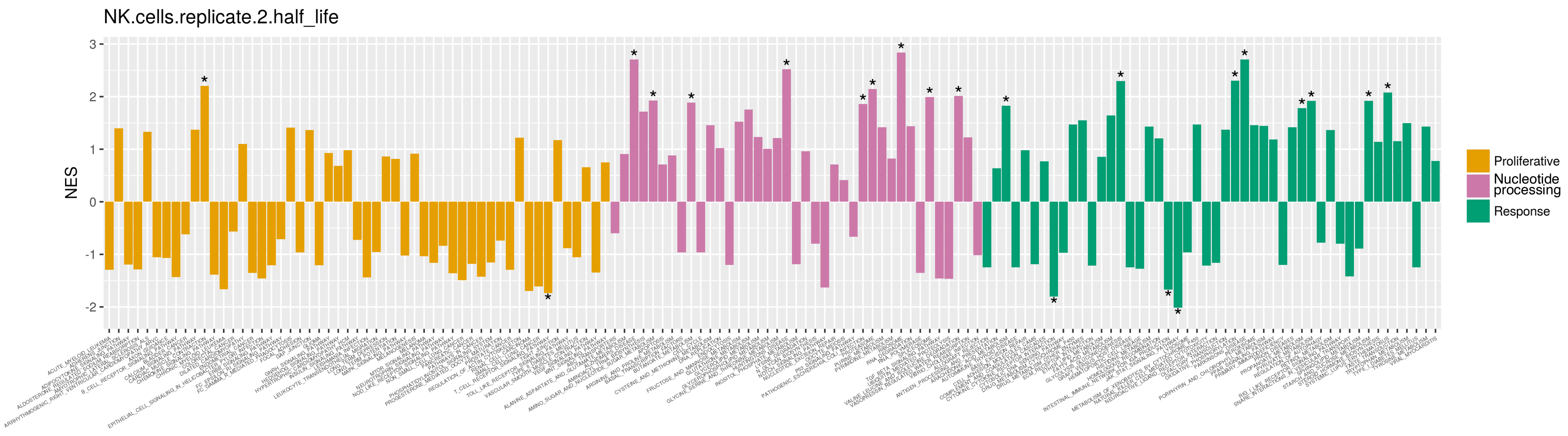
NK.cells.replicate.2.half_life
NES
3
2
1
0
-1
-2
Proliferative
Nucleotide processing
Response

Hepatocytes.replicate.1.half_life

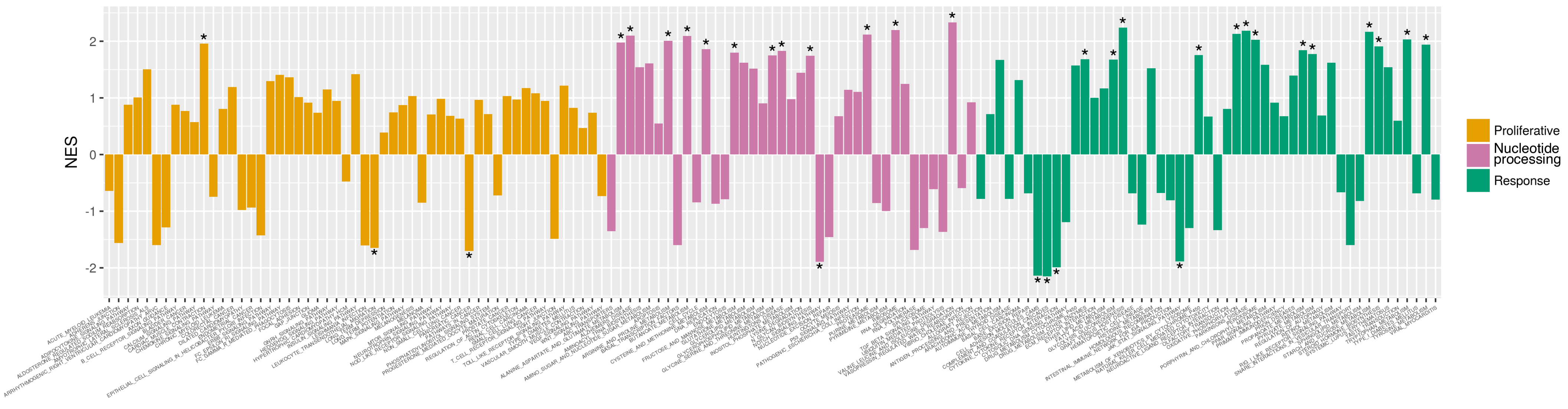

Hepatocytes.replicate.2.half_life

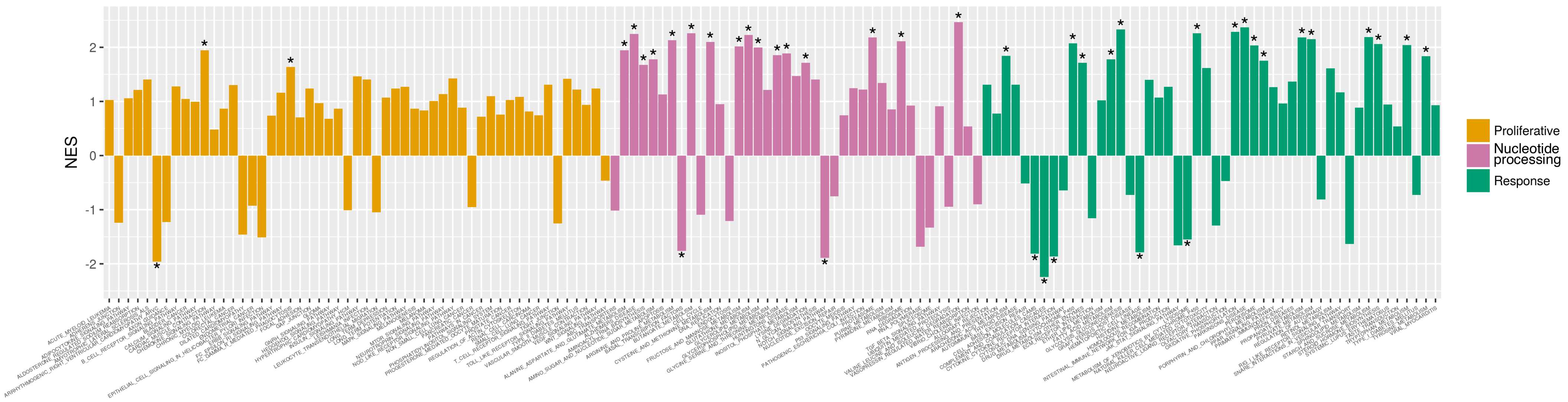

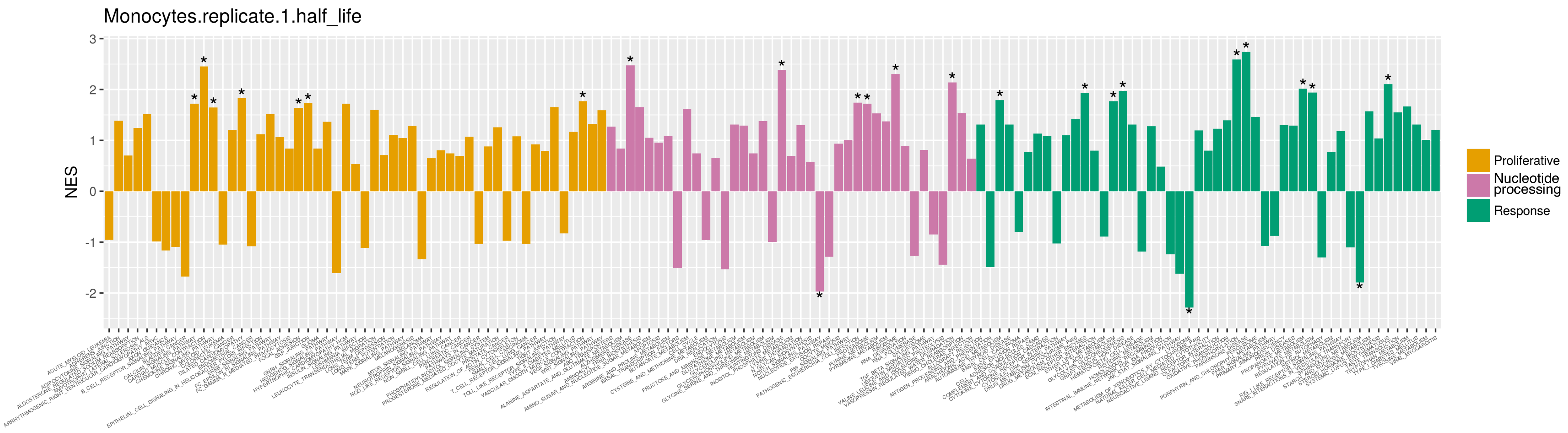
Monocytes.replicate.1.half_life
NES
3
2
1
0
-1
-2
Proliferative
Nucleotide processing
Response

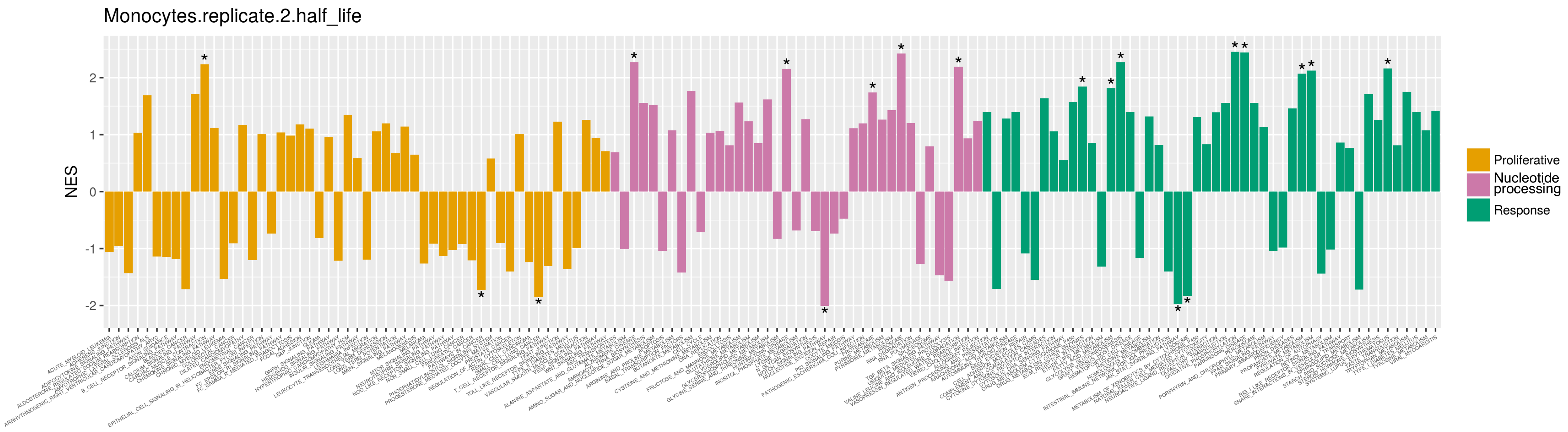
Monocytes.replicate.2.half_life
NES
2
1
0
-1
-2
Proliferative
Nucleotide processing
Response
