# Supplementary material for: Pathogenic missense protein variants affect different functional pathways and proteomic features than healthy population variants
Source: PLoS Biol. 2021 Apr 28;19(4):e3001207. doi: 10.1371/journal.pbio.3001207 (PMC8110273; doi:10.1371/journal.pbio.3001207)
Supplement: S15 Fig — (PDF) [file pbio.3001207.s018.pdf]

## S15 Fig

# The density of rare mutations from the gnomAD data in different protein regions

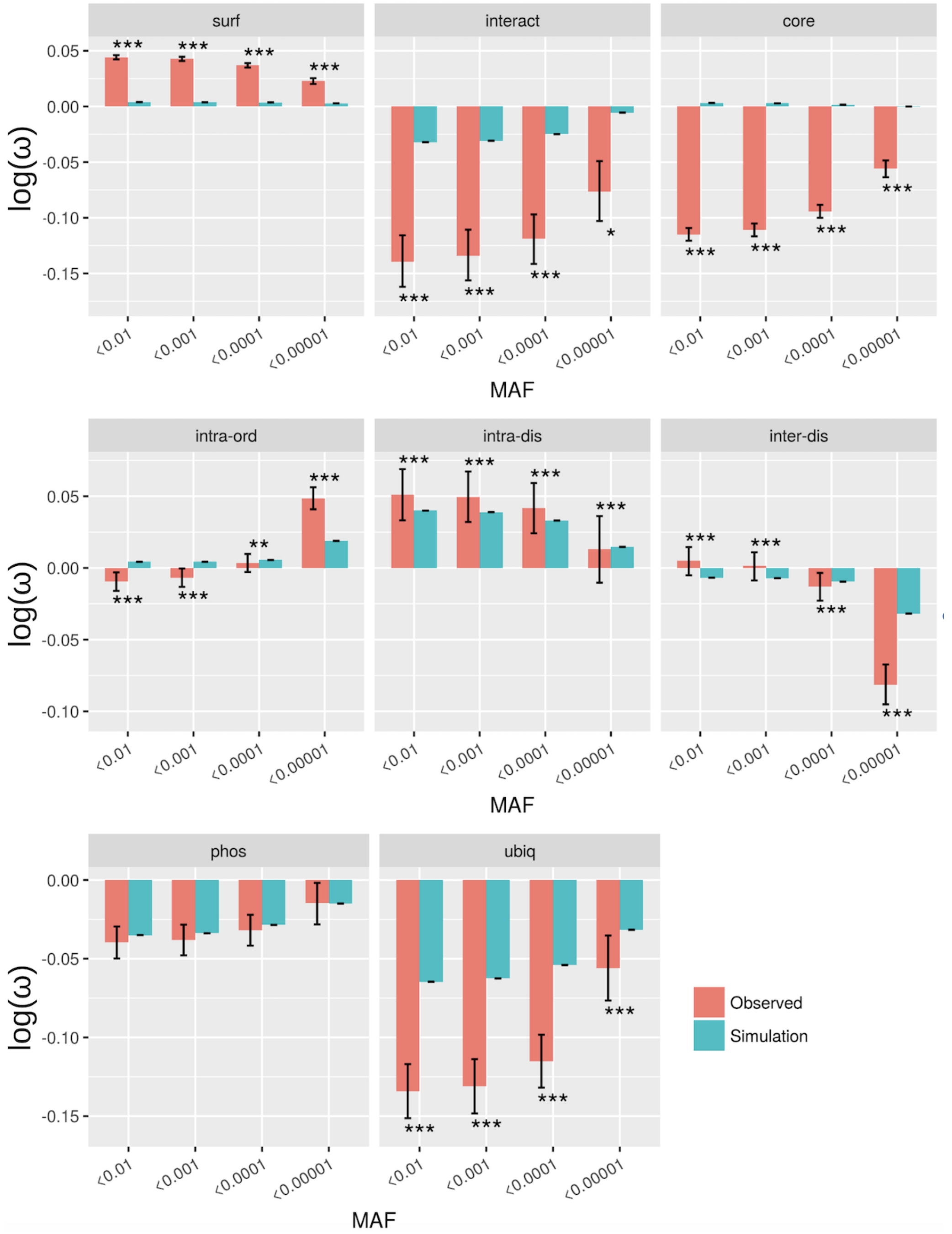


Rare variants have been defined using different MAF cutoffs as shown in the x axis. Both observed densities (pink), and densities derived from simulated null distributions (turquoise) are shown. Density ($\omega$) values were taken logarithm such that negative values indicate depletion while positive values indicate enrichment. Error bars depict 95% confidence intervals, for observed densities these were obtained by bootstrapping. Significance was calculated by comparison of observed values to simulated null missense variant distributions (significance level indicated by: * q-value $< 0.05$, ** q-value $< 0.001$, *** q-value $< 0.0001$). See S2 Data for the underlying data for the observed statistics, and S12 Data for statistics calculated in individual realisations of the simulated null distribution.
