# Supplementary material for: Pathogenic missense protein variants affect different functional pathways and proteomic features than healthy population variants
Source: PLoS Biol. 2021 Apr 28;19(4):e3001207. doi: 10.1371/journal.pbio.3001207 (PMC8110273; doi:10.1371/journal.pbio.3001207)
Supplement: S16 Fig — (PDF) [file pbio.3001207.s019.pdf]

S16 Fig

The distrbution and density in protein structural regions of variants classified by CADD

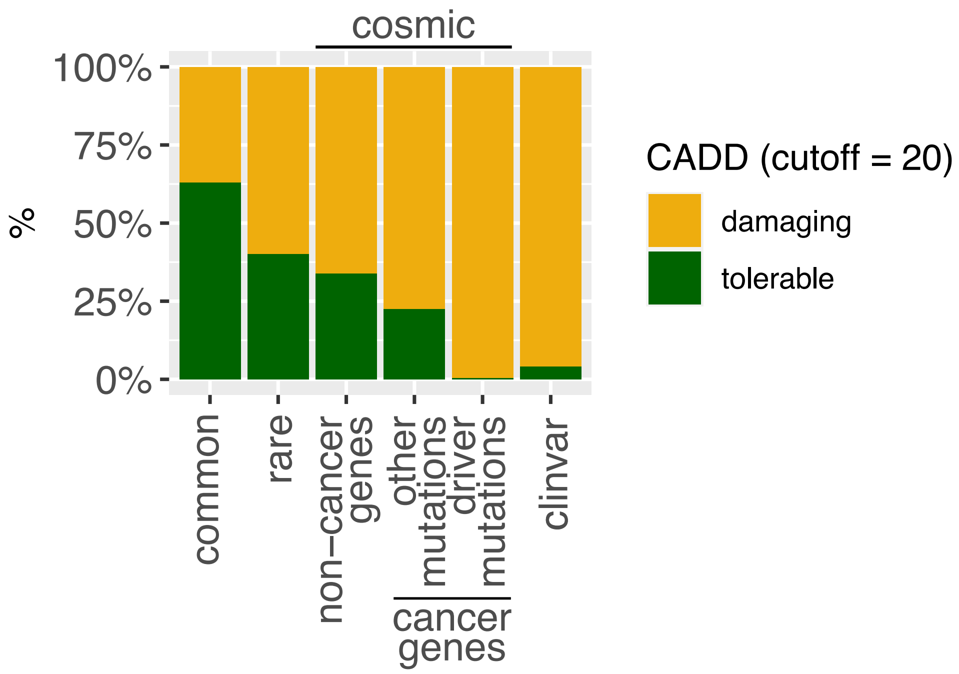

Panel A: Breakdown of variant impact classified by CADD for the four variant sets. See S13 Data for the underlying data.

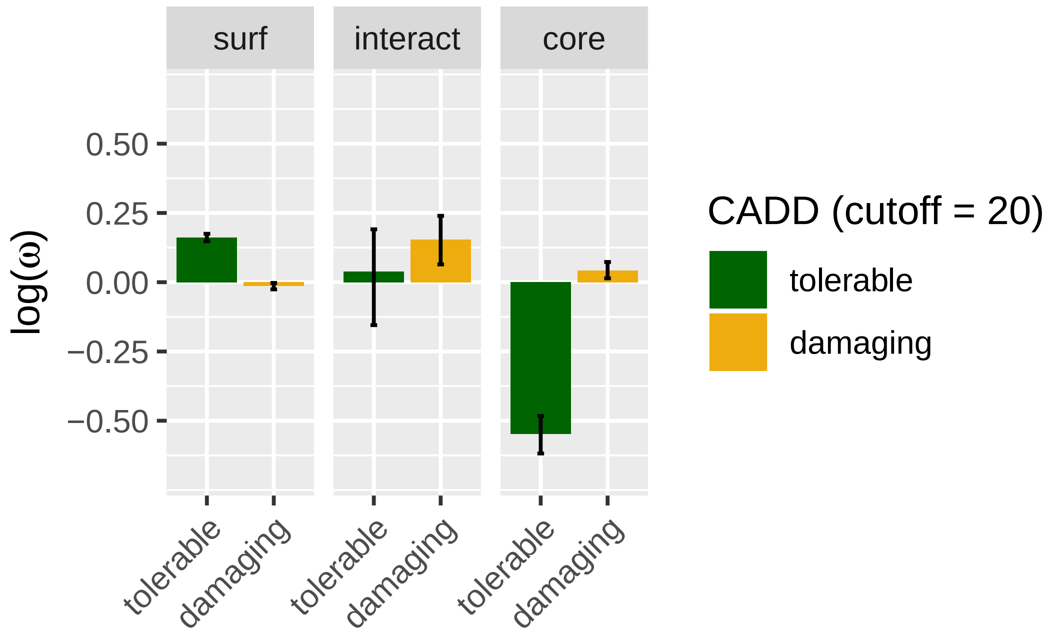

Panel B: The density in different protein regions for variants classified by CADD. Variants are classified into "tolerating" and "damaging" according to the CADD phred score using a cut-off of 20. Density (ω) values were taken logarithm such that negative values indicate depletion while positive values indicate enrichment. Error bars depict 95% bootstrapped confidence intervals. See S13 Data for the underlying data.
