# Supplementary material for: Pathogenic missense protein variants affect different functional pathways and proteomic features than healthy population variants
Source: PLoS Biol. 2021 Apr 28;19(4):e3001207. doi: 10.1371/journal.pbio.3001207 (PMC8110273; doi:10.1371/journal.pbio.3001207)
Supplement: S17 Fig — (PDF) [file pbio.3001207.s020.pdf]

S17 Fig

The association between VES and protein stability for variants scored by CADD and REVEL

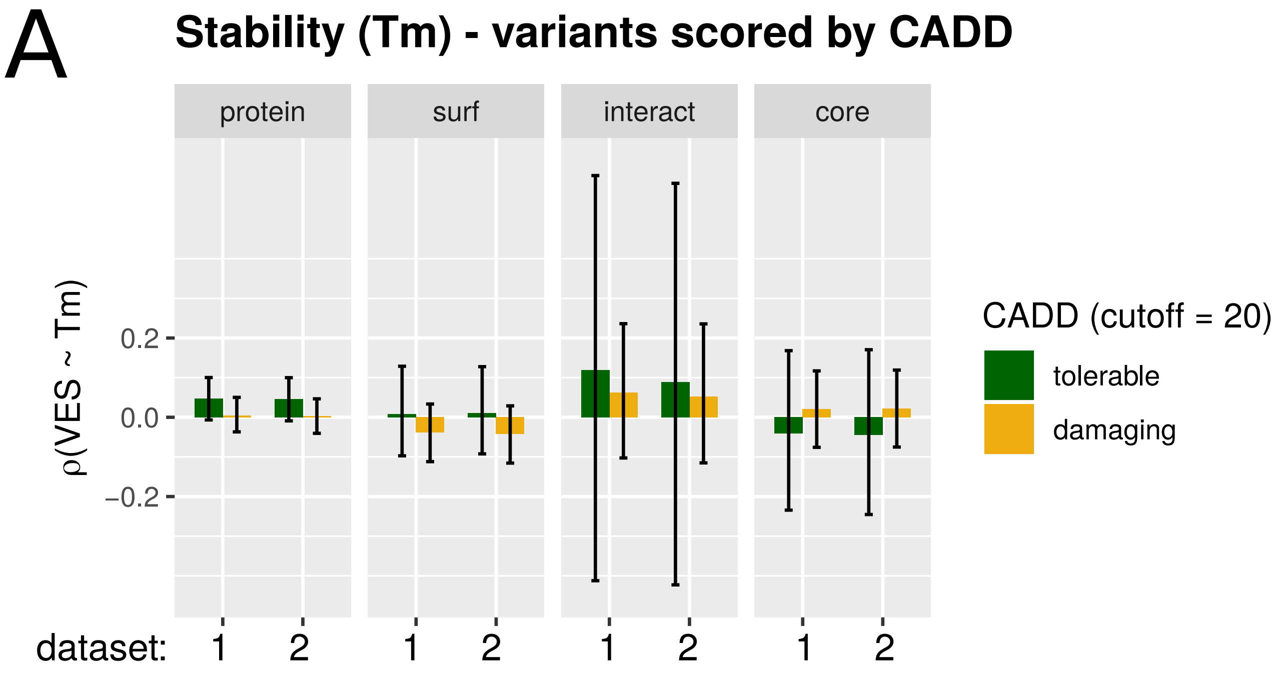

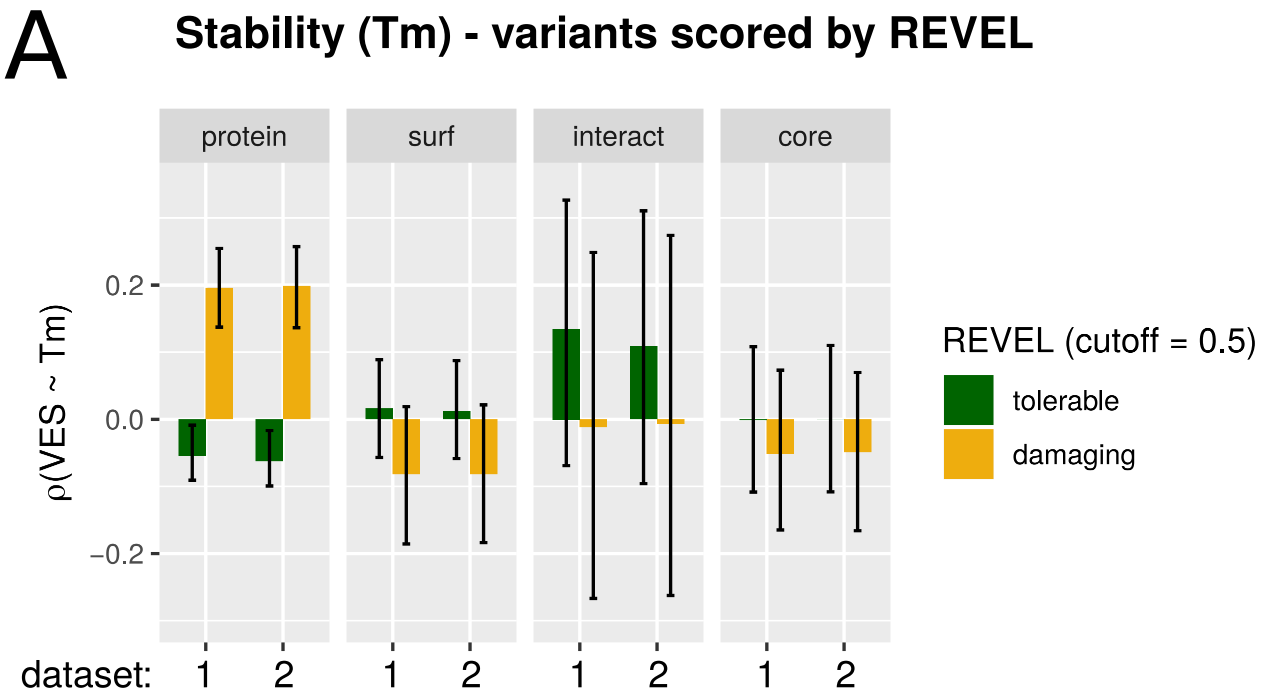

The association between VES and protein stability for variants scored by (A) CADD and (B) REVEL. Variants are classified into tolerating and damaging according to the cutoffs discussed in the publications of CADD and REVEL. The vertical axis depicts the Spearman correlation (ρ) between stability (melting temperature, Tm) and VES. Bars represent medians from 1,000 bootstrapped samples. Error bars depict 95% bootstrapped confidence intervals. See S13 Data for the underlying data.
