# Supplementary material for: Pathogenic missense protein variants affect different functional pathways and proteomic features than healthy population variants
Source: PLoS Biol. 2021 Apr 28;19(4):e3001207. doi: 10.1371/journal.pbio.3001207 (PMC8110273; doi:10.1371/journal.pbio.3001207)
Supplement: S18 Fig — (PDF) [file pbio.3001207.s021.pdf]

S18 Fig

The association between VES and protein abundance for variants scored by CADD and REVEL

The association between VES and protein abundance for variants scored by (A) CADD and (B) REVEL. Variants are classified into tolerating and damaging according to the cutoffs discussed in the publications of CADD and REVEL. The vertical axis depicts the Spearman correlation (ρ) between protein abundance and VES. Here the complete data are shown, in contrast to the main text where only selected organs/tissues are represented. Bars represent medians from 1,000 bootstrapped samples. Error bars depict 95% bootstrapped confidence intervals. See S13 Data for the underlying data.

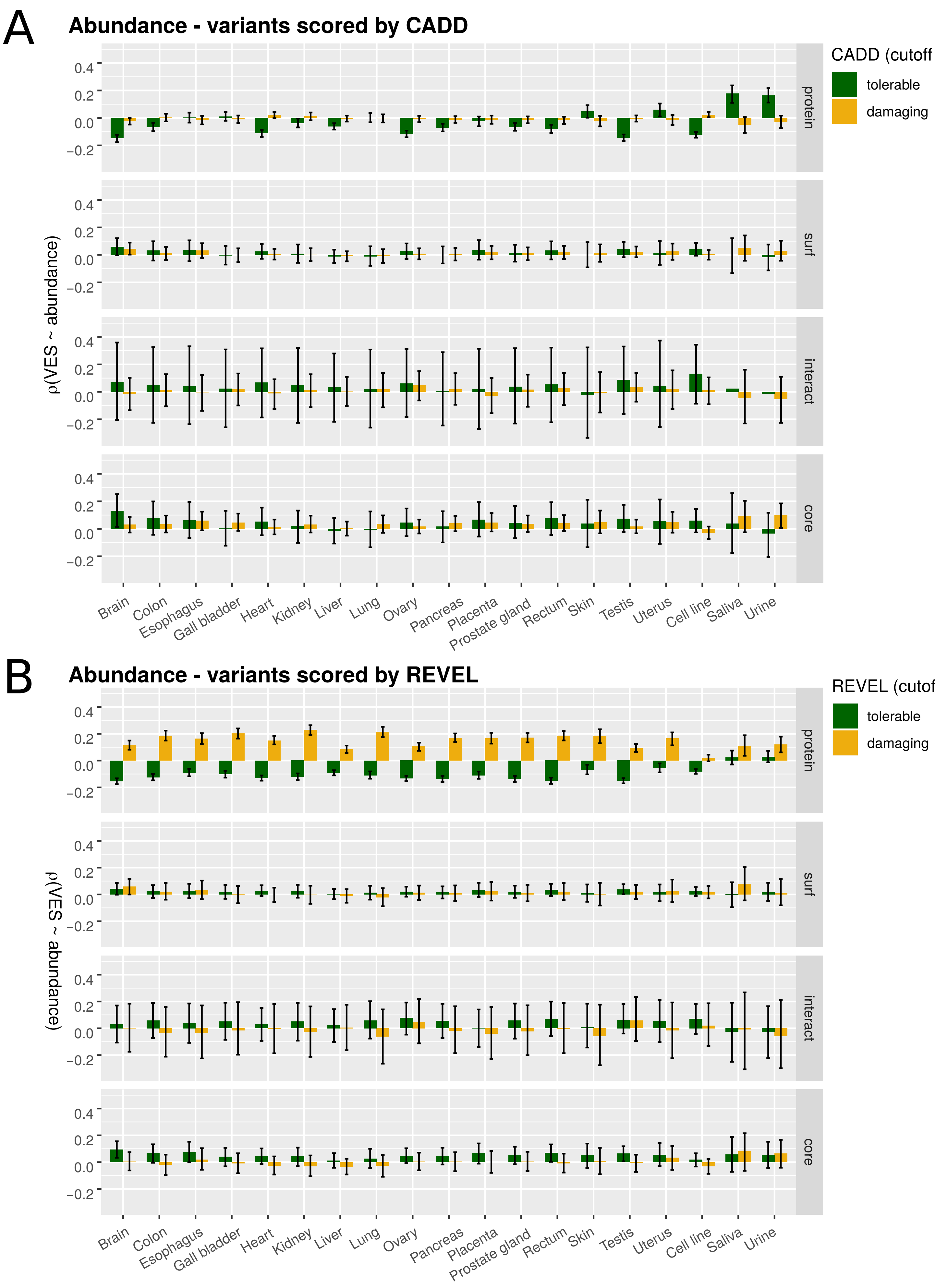
A
Abundance - variants scored by CADD
CADD (cutoff
tolerable
damaging
protein
surf
interact
core
ρ(VES ~ abundance)
0.4
0.2
0.0
−0.2
Brain
Colon
Esophagus
Gall bladder
Heart
Kidney
Liver
Lung
Ovary
Pancreas
Placenta
Prostate gland
Rectum
Skin
Testis
Uterus
Cell line
Saliva
Urine
B
Abundance - variants scored by REVEL
REVEL (cutof
tolerable
damaging
