# Supplementary material for: Pathogenic missense protein variants affect different functional pathways and proteomic features than healthy population variants
Source: PLoS Biol. 2021 Apr 28;19(4):e3001207. doi: 10.1371/journal.pbio.3001207 (PMC8110273; doi:10.1371/journal.pbio.3001207)
Supplement: S19 Fig — (PDF) [file pbio.3001207.s022.pdf]

S19 Fig

Pathway enrichment analysis for tolerable and damaging variants as defined by CADD

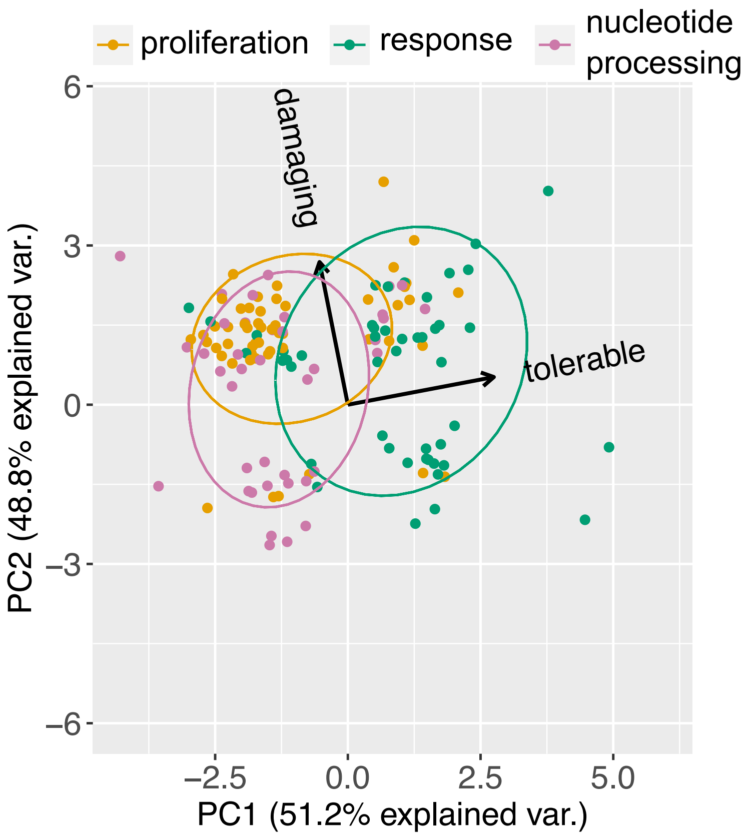

Pathway enrichment analysis for tolerable and damaging variants as defined by CADD. See S13 Data for the underlying data.
