# Supplementary material for: Pathogenic missense protein variants affect different functional pathways and proteomic features than healthy population variants
Source: PLoS Biol. 2021 Apr 28;19(4):e3001207. doi: 10.1371/journal.pbio.3001207 (PMC8110273; doi:10.1371/journal.pbio.3001207)
Supplement: S1 Appendix — (PDF) [file pbio.3001207.s036.pdf]

S1 Appendix

Detailed Description of Data Files

The following data files can be downloaded online on the article page online, or alternatively at fraternalilab.kcl.ac.uk/ZoomVar/downloads/.

Here, individual sheets within spreadsheet collections are listed with the corresponding figures/tables to which the data were presented wherever applicable.

S1 Data Details of the number of missense variants which localise to different protein regions in the gnomAD common and rare, COSMIC and ClinVar datasets.

S2 Data Variant density and VES of protein regions and domains. GSEA of structural/disorder regions VES.

- Density of ClinVar/common/rare/cancer driver/non-cancer driver variants in protein regions defined by structures and disorder. (*Fig 2, S3 Fig*)
- Density of gnomAD rare variants defined using different MAF cutoffs, in protein regions defined by structures and disorder. (*Fig 6E-G, S15 Fig*)
- Gene Set Enrichment Analysis (GSEA) of Variant Enrichment defined in protein and protein regions. (*Fig B,C in S3 Text*)
- Whole-protein VES and adjusted p-values. (*S1 Fig*)
- Surface VES and variant density. (*S2 Fig*)
- Correlation between protein core density metric (mean residue contacts) and Variant Enrichment Score for protein cores. (*S10 Fig*)
- Whole-domain and domain region VES and adjusted p-values. (*Fig 3A-B, Fig E in S3 Text, S4 Fig*)
- Number of drugs targeting each Pfam domain-type. (*Fig 3C*)
- Enrichment of CATH architecture in Pfam domain-types according to missense variant enrichment. (*Fig D in S3 Text*)

S3 Data Density of variants in protein regions in simulations of random variants. (*S3 Fig*)

S4 Data Proteins enriched in COSMIC non-cancer gene variants at protein-protein interaction sites.

S5 Data Statistics for comparisons of structural network features between datasets. (*Fig A in S3 Text*)

S6 Data Gene Set Enrichment Analysis (GSEA) of protein VES calculated on ClinVar/COSMIC/common/rare variant sets.

- GSEA on protein VES. Rare variants defined as gnomAD variants with MAF < 0.01. (*Fig 4B-E, Fig 6A, S5 Fig*)
- GSEA on protein VES. Rare variants defined as gnomAD variants with MAF < 0.001. (*Fig 6B*)
- GSEA on protein VES. Rare variants defined as gnomAD variants with MAF < 0.0001. (*Fig 6C*)
- GSEA on protein VES. Rare variants defined as gnomAD variants with MAF < 0.00001. (*Fig 6D*)

S7 Data A list of pathways annotated by functional cluster (”proliferation”, ”nucleotide processing” and ”response”). (*Fig 5B-E*)

S8 Data Numbers of proteins and missense variants which underlie correlations between proteomic/transcriptomic features and variant enrichment.

S9 Data Proteomics features and correlation with variant enrichment scores.

- Stability values of proteins enriched in each variant type. (*S6 Fig*)
- Abundance values of proteins enriched in each variant type. (*S6 Fig*)
- Correlation between protein stability and variant enrichment score (VES). (*Fig 5A, S7 Fig panel A*)
- Correlation between protein abundance and variant enrichment score (VES). (*Fig 5B, S7 Fig panel B*)
- Correlation between protein & transcript abundance and variant enrichment score (VES). (*S8 Fig*)
- Correlation between protein half-life and variant enrichment score (VES). (*S9 Fig*)

S10 Data GSEA of proteomics and transcriptomic measures.

- GSEA of protein stability data. (*Fig 5C, S11 Fig*)
- GSEA of protein abundance data. (*S12 Fig*)
- GSEA of transcript expression data. (*S13 Fig*)
- GSEA of protein half-life data. (*S14 Fig*)

S11 Data Pairwise Spearman correlations between all studied proteomics and transcriptomics features.

S12 Data Density of rare variants under different MAF cutoffs, in simulations of random variants. (*Fig 6E-G, S15 Fig*)

S13 Data Comparison of variant enrichment and associations of damaging and tolerable variants as annotated by orthogonal impact predictors.

- Breakdown of variants by source variant sets in tolerable and damaging variants as labelled by REVEL and CADD. (*Fig 7B, S16 Fig*)
- Variant density of tolerable and damaging variants as labelled by REVEL and CADD. (*Fig 7C, S16 Fig*)
- Correlation of Protein stability and VES of tolerable and damaging variants as labelled by REVEL and CADD. (*Fig 7D, S17 Fig*)
- Correlation of Protein abundance and VES of tolerable and damaging variants as labelled by REVEL and CADD. (*Fig 7E, S18 Fig*)
- Enrichment of variants in surface/core/interacting interface of a representative bootstrapped sample. (*Fig 7F*)
- GSEA analysis of tolerable & damaging variants as labelled by REVEL. (*Fig 7G*)
- GSEA analysis of tolerable & damaging variants as labelled by CADD. (*S19 Fig*)
