# Supplementary material for: Pathogenic missense protein variants affect different functional pathways and proteomic features than healthy population variants
Source: PLoS Biol. 2021 Apr 28;19(4):e3001207. doi: 10.1371/journal.pbio.3001207 (PMC8110273; doi:10.1371/journal.pbio.3001207)
Supplement: Attachment — Submitted filename: reviewer_response.pdf [file pbio.3001207.s037.pdf]

# Response to reviewers

November 27, 2020

**Missense variants in health and disease affect distinct functional pathways and proteomics features**
Anna Laddach, Joseph Chi-Fung Ng & Franca Fraternali

We would like to thank for the constructive comments from the three reviewers. Here we include a point-by-point response to these comments (reproduced here, highlighted in red). Changes in the manuscript are highlighted in blue.

Wherever possible we have referenced the relevant line numbers where changes have been made to the main manuscript; these were taken from the revised version with track changes.

## Reviewer 1

Laddach, Ng et al. attempt to quantify variant enrichment in human proteins to decipher basic principles underlying the differences between pathogenic and benign variants. This is a very important topic of study and its impact to disease researchers is high. The author's develop a measure, VES, of determining enrichment of variants across several levels of protein hierarchy (i.e. entire protein, domains and domain-type). The author's also incorporate proteomic features such as a protein half-life into their analysis providing a unique evaluation that is much needed in the field. Unfortunately, I have deep concerns about the underlying statistics and ultimately the interpretability of the VES measure which impact much of manuscript. Due to these concerns, the conclusions of the paper are all in question.

We thank Reviewer 1 for his/her comments. We acknowledge that the original version of the manuscript reads very dense and discussions on the derivation and interpretation of the Variant Enrichment Score (VES) may be difficult to grasp. We have explained in detail below these concepts; we believe these discussion is useful to readers and it has been included as a Supplementary Note to explain the motivation of our formulation of VES. We have also made a number of important and relevant changes in the revised version to help readers interpret VES statistics; hopefully these explanations and changes will clear the Reviewer's doubt.

**Major**:

The authors develop a Variant Enrichment Score (VES) to "... assess the missense variant enrichment of a given entity...". Their formulation of the binomial test in computing the VES is incorrect. The binomial test requires definitions of $k$, $n$ and $p$. To test enrichment in a given entity, $k$ should equal the number of missense variants in the entity (as the authors properly define), $n$ should be the size of the entity and $p$ should be the expected probability of missense

variants in the entity. The author's define $n$ as "the total number of missense variants which localise to all relevant entities" and $p$ as "the ratio of the size of the entity of interest (in terms of number of residues) to the total size of all relevant entities". It is unclear how these definitions of $n$ and $p$ substitute for the expected definitions and likely result in uninterpretable results.

We agree that Reviewer 1 has put forward a classical formulation of the binomial distribution in modelling the enrichment of variants. The formulation we adopted was taken from Porta-Pardo and colleagues [1], who applied this to identify protein interfaces enriched in cancer variants. We generalised this to look not only at interfaces, but also other regions of proteins.

We believe our formulation is an equally valid approach to model variant enrichment. Below we include a detailed statement to illustrate Reviewer 1's formulation and our formulation and compare their similarities and differences. We show below that these two formulations generate the same numerical behaviour in calculating the variant enrichment statistics. However, they would give different results in cases where variants are unevenly distributed across the protein. For the sake of simplicity we discuss the case of variant enrichment within a region (surface/core/interface) of a given protein; these can be generalised to other forms of "entity" (domain, domain-type etc.) that we have covered in the text. In this explanation we consider a protein with complete structural coverage, i.e. every position could be assigned to any one of surface, core or interface.

## Approach 1: Generating mutations

This is the approach that Reviewer 1 named. To model variant enrichment, here we start from a given region of a protein (let the size of this region be $n$), and view the *generation* of variants at different positions to be independent from one another. Assuming there is an equal probability to generate a variant at every position, we walk through each position in the region, and perform one "trial" to generate a mutation with probability $p$. Repeating this for all positions within the region, we finally ask what is the probability that $k$ of those positions are mutated. (Figure 1A)

We can model $X$, the number of variants in the region, as a binomial random variable, with the probability density function:

$$P(X = k) = \binom{n}{k} p^k (1 - p)^{n-k}$$

$P(X = k)$ is the probability that $k$ variants have been found in this region. $p$ is the expected chance of a given position to harbour a variant, which, under a frequentist approach, could be formulated as

$$p = \frac{\text{number of mutated residues}}{\text{total number of residues}}$$

In this formulation each "trial" in a binomial process refers to generating a mutation at one given position in the region, and we ask whether this position harbours a variant. This formulation assumes an equal chance for every position to be mutated.

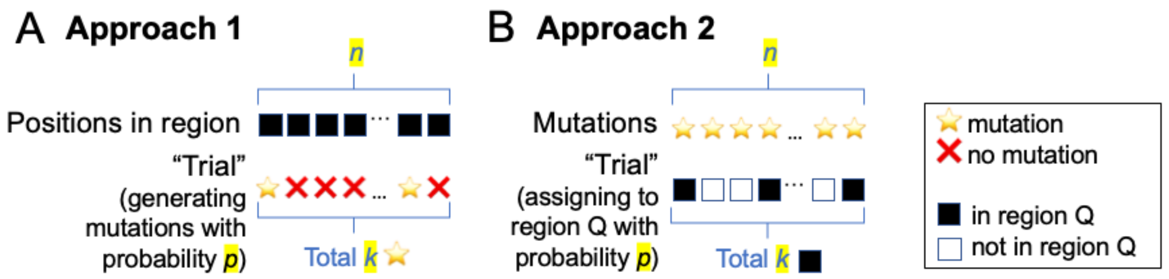


Figure 1: Comparison of two binomial formulations in modelling variant distribution. Illustration of the two approaches for this hypothetical scenario of modelling variant enrichment of this protein in a defined region Q.

## Approach 2: Distributing mutations

We took another way to approach this problem, starting with a given number ($n$) of mutations, and computing the probability that a subset (totalling $k$ mutations) of them localise to a given region: each "trial" here refers to assigning a mutation to a region. The probability of assigning the mutation to our region of interest is $p$ (Figure 1B).

Under a "null" scenario there is no bias towards localising to any region, therefore the expected probability $p$ would simply be the ratio of the size of the region in question to the total size of the protein:

$$p = \frac{\text{number of residues in the region}}{\text{total number of residues}}$$

With these alternative definitions of $n$, $k$ and $p$, this formulation explicitly models the distribution of mutations: we are constructing a probabilistic model to compare the observed *distribution* of variants across different regions to an expectation based only on the sizes of these regions. This "null" expectation is again a binomial process (therefore has the identical form as the $P(X = k)$ expression above): each "trial" refers to *assigning* a variant to any region of the protein.

## Comparing these formulations of quantifying variant enrichment

The following table summarise the specification of the parameters of a binomial model (i.e. $n$, $k$ and $p$) in both Approach 1 and Approach 2.

| | **Approach 1** | **Approach 2** |
|---|---|---|
| $n$ | Number of residues in the region | Number of mutated residues |
| $k$ | Number of mutated residues in the region | Number of mutated residues in the region |
| $p$ | $\frac{\text{number of mutated residues}}{\text{total number of residues}}$ | $\frac{\text{number of residues in the region}}{\text{total number of residues}}$ |

Table 1: Summary of the parameters ($n$, $k$ and $p$) of the binomial model in the two approaches explained above.

In the following, we demonstrate that these two formulations model the same problem, and exhibit the same numeric behaviour.

### Cumulative distribution function (CDF)

The distribution of $X$ is a discrete probability distribution modelled on a binomial process as detailed above, under either formulation of the problem. Using this one can quantify the *extent* to which variants are localised to a given region. $P(X = k)$ is a discrete probability of observing $k$ variants. The extent of accumulating $k$ variants would be the cumulative likelihood doing so, i.e. the sum of likelihoods to observe 0, 1, ... $k-1$, $k$ variants in this region:

$$P(X \leq k) = \sum_{i=1}^{k} \binom{n}{i} p^i (1-p)^{n-i}$$

This expression, $P(X \leq k)$, is equivalent to the *cumulative distribution function* (CDF) of the distribution of X. This CDF is taken in the manuscript as the Variant Enrichment Score (VES).

Notably, by definition, each $P(X = i)$ (for $i = 1, 2, ...k$) is bounded by [0, 1] and $\sum_{\forall i} P(X = i) = 1$, therefore the CDF monotonically increases as $k$ increases and is bounded by [0, 1].

Figure 2 below shows a numerical and hypothetical example which demonstrates this point, with the VES (i.e. CDF) increases as the number of variants in the region increases:

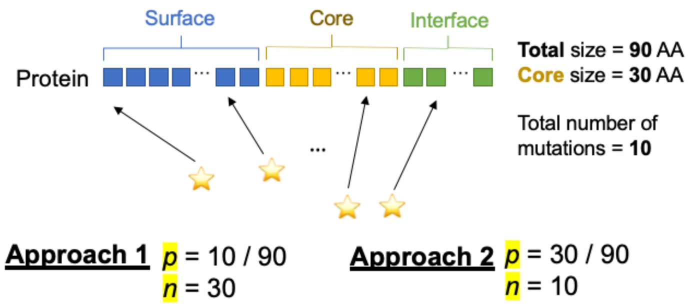


| number of core mutations (k) | Approach 1 | Approach 2 |
|---|---|---|
| 1 | CDF = 0.1387 | CDF = 0.1040 |
| 4 | CDF = 0.7642 | CDF = 0.7868 |
| 8 | CDF = 0.9958 | CDF = 0.9996 |

Figure 2: Hypothetical example of different binomial formulations in calculating variant enrichment. (*left*) Set-up of this hypothetical scenario; we are interested in the variant enrichment of the core (size = 30 amino acids [AA]) of this 90-AA protein. 10 mutations have been mapped to this protein. The definitions of $n$ and $p$ of the two binomial formulations are listed. (*right*) Table showing the cumulative distributive function (CDF) calculated under the two binomial formulations for specific number of variants ($k$) observed in the core of this hypothetical protein.

### Expected value

With the binomial process, one can write an expression for the expected value of the experiment:

$$E(X) = np$$

In our problem, this corresponds to the expected number of mutated residues in the given region. Note that in either formulation, this expectation is identical; referring to Table 1, for Approach 1,

$$E(X) = \text{Number of residues in the region} \times \frac{\text{number of mutated residues}}{\text{total number of residues}}$$

For Approach 2 (i.e. our approach),

$$E(X) = \text{Number of mutated residues} \times \frac{\text{number of residues in the region}}{\text{total number of residues}}$$

The two expressions are identical. Together with the behaviour of the CDF, this demonstrates the two formulations have identical numerical behaviours, and essentially models the same problem.

### Differences

The way differences in protein (and therefore region) sizes are handled are slightly different, although as shown above the two approaches model variant enrichment similarly. For Approach 1, clearly $n$ changes with protein size, as does the denominator (i.e. "total number of residues") of $p$ (Table 1). For example, for a larger protein with a larger region-of-interest, $n$ increases and $p$ decreases; this compensatory changes between $n$ and $p$ allow for consideration of variable protein/region sizes across different proteins. Approach 2 (the formulation we adopted), on the other hand, only considers the *assignment* of mutations to regions (Figure 1B). The changes in protein and region size are internalised within $p$ (Table 1). However, as shown above, this difference between the two formulations does not imply different numerical behaviours.

There are possible cases which result in different behaviours of variant enrichment statistics generated by the two formulations. If there are multiple different variants localised to the same position, the two formulations will treat such cases differently. In Approach 1, since each binomial "trial" is the generation of mutation, multiple variants would only be counted once. This could potentially underestimate variant enrichment. This problem does not exist in Approach 2 (i.e. our formulation), as in that case the binomial experiment amounts to assigning mutations to regions. Multiple variants on the same position would be distributed to the same region. The following (Table 2) illustrates the difference in CDF calculations in the two models, both for a real-life example (variant enrichment in the VHL protein in the COSMIC dataset) and a hypothetical, synthetic example based on the same protein.

As shown in Table 2, Approach 2 is robust to the possibility of having multiple variants mapped on each position whereas Approach 1 underestimates the enrichment if variants are highly concentrated within a small number of positions. In this work we therefore chose Approach 2 (i.e. distributing/assigning variants as the binomial "trial"). We think that Figure 1 in the main text and the methods section currently do illustrate the formulation and interpretation of VES, so we are keeping these materials. However, we think it would be useful to include the discussion above in the Supplementary Materials as a note of the motivation of our method (new section "Note on probabilistic methods to model variant enrichment"). We also think the other changes in relation to the following comments from Reviewer 1 would aid readers to understand the VES formulation (see below).

| | **VHL protein in COSMIC dataset** | **A hypothetical example** |
|---|---|---|
| Size (number of residues) | 115 (Surface) & 36 (Core) | 115 (Surface) & 36 (Core) |
| Number of variants | **Surface**: 117 on 60 positions<br>**Core**: 81 on 23 positions | **Surface**: 117 on 114 positions<br>**Core**: 81 on 5 positions |
| CDF | Approach 1<br>**Surface**: 0.305<br>**Core**: 0.894<br><br>Approach 2<br>**Surface**: $8.15 \times 10^{-8}$<br>**Core**: 1 | Approach 1<br>**Surface**: 1<br>**Core**: $1.55 \times 10^{-16}$<br><br>Approach 2<br>**Surface**: $8.15 \times 10^{-8}$<br>**Core**: 1 |

Table 2: Illustration of Approach 1 and Approach 2 applied on a real-life case of calculating variant enrichment (VHL protein in the COSMIC dataset) and a hypothetical, synthetic example designed based on the VHL protein. As shown in the table the protein in question is identical to one another (same sizes of core and surface regions), but in the hypothetical example variants in the core are concentrated in a small number of positions.

The manuscript is lacking validation of the Variant Enrichment Score. A global analysis of how the VES behaves on known enriched examples (positive controls) as well as known depleted examples (negative controls) would be beneficial for evaluation. Individual positive control examples for each entity type (i.e. protein, domain and domain type) would also aid the reader in how to interpret the VES.

We do acknowledge that presentation of certain control cases would help in understanding the interpretation of VES. We consider the analyses on oncogenes and tumour suppressor genes (TSGs) (originally main text Figure 5D) as an example of "known enriched/depleted" cases, which could be presented as controls. The observations that oncogenes are enriched in mutations on surfaces and interfaces, and TSGs enriched in core mutations, have been made in numerous occasions using different methods [2, 3, 4]. Our VES formulation recapitulates these results, and provides a straightforward case study to understand how VES should be interpreted.

In the revised version we move forward this figure panel and the associated descriptions (new Figure 1E) alongside our introduction of the concept of VES. We believe these shall aid the readers to understand the interpretation of VES.

Figure 6A shows P53 full length domain-type with a VES score of being depleted in gnomad_common. My expectation would be that at least one of the three individual "domain-type structural regions" (i.e. surf, interact or core) in figure 6B would show depletion for gnomad_common and none would show enrichment. Figure 6B shows all three domain-types as enriched. Can the author's explain the inconsistency?

Figure 6A shows that P53 domains are depleted in gnomAD_common. This calculation, continuing from our discussion above on VES, is considered relative to all other domains ($n$ is the total number of variants mapping to any domain, $k$ is the variants mapped to P53 domains). In

other words, it implies that out of all variants in gnomAD_common, we have less-than-expected variants mapped to P53 domains. VES values in Figure 6B refer to a different comparison: *given* the total number of gnomAD_common variants in the P53 domain, how enriched are these in surface/core/interface? We are essentially asking, within the already-variant-depleted P53 domains, how are variants distributed. Therefore 6A and 6B address two separated questions and the patterns observed in the regions (6B) are relative only to the other regions (since in our VES calculation we are quantifying probabilities of assigning variants to different regions, therefore depletion in one region would essentially imply enrichment of another), *not* to the enrichment/depletion in the entire domain-type. We wonder whether a source of confusion here stems from the fact that we only provide one heat scale (referencing "VES") on Figure 6, which might mislead readers to think the VES in 6A and 6B was calculated with the same "reference point". In the revised version we included separate legends for panels A and B and specified their difference.

On the observation that VES for all three structural regions appear enriched: this is actually a result of the low number of gnomAD_common variants which localise to this domain. Only 1 gnomAD_common variant localises to the P53 domains, and it maps to the surface. As a result the binomial cumulative distribution function (CDF) - i.e. VES - for surface is 1. For the interface and core the CDF is also high as the probability of observing 0 variants in these regions, given that only 1 variant localises to the P53 domain, is high. In fact the $p$-value for the two-tailed binomial test is 1.

While readers can always refer to the database for these p-value statistics, we do acknowledge and apologise for the confusion caused by this point, as statistical significance (or insignificance) should be made clear on the figures to avoid misleading the readers. In the revised version, instead of these heatmaps, we generated heat-coloured bubble plots where the size of bubbles scale with the level of statistical significance, such that VES with weaker statistical support are visualised "smaller" than those with better support. We think this is a fairer way to present these data, and will help the readers interpret variant enrichment.

**Minor**:

Figure 3 legend refers to "red arrows" that are absent from figure.

We apologise for this error - we mean the black arrows on the PC plot. This is now corrected.

## Reviewer 2

In this interesting work by Laddach, Ng, and Fraternali, the authors enumerate a series of protein-centric features and combine these with available genetic variation data from COSMIC, ClinVar and gNOMAD resources to create a data resource (ZoomVar), and to compute variant enrichments relative to these features. Enrichments are calculated using a binomial test and a density-based metric. The authors report some findings consistent with previously reported mechanisms for rare disorders and oncogenesis. While many aspects of the presented research are appealing (the examination of protein interfaces, disordered regions, etc.) the overall presentation and message of the manuscript is very difficult to follow as it is currently structured. For example, the authors state in their conclusion that their work highlights the complex interplay between different factors that influence pathogenicity - this was not in doubt given the prior literature. They then state that "these insights" will be useful for variant prioritization, but it is unclear what specific insights they are referring to. I am entirely sympathetic to the tremendous effort in compiling these data, but I would suggest refocusing the manuscript to emphasize fewer, more concise points you have identified from your analyses.

We thank Reviewer 2 for his/her constructive comments. We do agree it could be challenging to read and follow through all aspects of our analyses; in the revised version we have prioritised some of the analyses and relegated the rest towards Supplementary Materials. We apologise for the unclear reference of "insights" in the Discussion - we have rewritten the relevant section (lines 713-719).

Specific comments and points of confusion are the following:

1. The abstract and much of the introduction prepares the reader for evaluations of variant pathogenicity, yet the authors do not propose any specific method for ranking variant pathogenicity. I think this is OK, but I would refocus the manuscript away from predicting variant pathogenicity and more toward understanding the critical features of proteins that induce phenotypic changes rather than focusing on the variants themselves.

2. The introduction is a bit broad in scope and doesn't provide an understanding of what this paper adds to the scientific literature, or at best does so in vague terms. Is there a specific finding from this paper that the authors wish to highlight? The importance of ordered versus disordered regions, etc?

   We agree with these two comments; the original introduction did read broad and general. We always have pathogenicity prediction in mind as we see this as the eventual destination of this line of research but we agree for this current manuscript it would be better to refocus the abstract, introduction and discussion as Reviewer 2 suggested. To us the most important aspects of this work are the followings:

   - The unbiased comparison of structural and pathway features targeted by missense variants of different natures. These have been attempted in the past (see related comment - point 3 below) but not in a uniform manner with an unbiased method motivated as ours.
   - An examination of the use of proteomics features in distinguishing missense variants.

   In the revised version we have rewrote the abstract and introduction to address comment 1 and select materials to highlight the relevance of important aspects of this work.

3. The authors state that 3D structure-based evaluation has not been performed on population or Mendelian disease variants, but several other studies the authors cite seem to have done this - 15, 16, 56.

   Apologies, we should have stated that an unbiased comparison on 3D structural impact has not been performed on the four variant subsets we considered. Specifically:

   - Ref. 15 [5] only compares disease variants with neutral identified in the 1000 Genomes project. This is much smaller in scale than the gnomAD database and there is no comparison of rare and common variant subsets.
   - Ref. 16 [6] only focusses on interaction sites.
   - Ref. 56 [7] looks at the spatial distribution of variants (i.e. variant "hotspots" in a more confined 3D space) rather than enrichment of more broadly-defined regions.

   We have removed this statement from the introduction, and clarified this point regarding comparison with prior literature in the newly added passages in the introduction.

4. The methods section could be expanded to include any filtering or normalization steps used on the variant sets, GTEx transcripts and PaxDB data. If no filtering was performing, it would be useful to explicitly state this.

Normalised data provided by GTEx and PaxDB were used directly without any filtering steps. This is now explicitly stated in the Methods (lines 117-118).

5. In general, the rationale and interpretation of the VES is not clear, and some elaboration on the interpretation, particularly with respect to multimers and the potential violations of assumptions surrounding protein size should be discussed. A distribution of the VES scores for each dataset would be useful to present, and it could be useful to generate a composite distribution of VES across all three datasets as a null distribution.

We hope our detailed description above in our response to Reviewer 1 would clear off this doubt on the rationale of VES. On the specific points raised here:

- **Multimers**: Homo-oligomers were *not* considered. This has now been explicitly stated (line 131).
- **Potential issues with protein size**: This relates to the quantity $p$ in our binomial formula. Since $p$ is a relative quantity comparing the size of the entity to the total size, this is a valid question. In the case of analysing structural regions, i.e. surface/core/interface are the "entities", and the size of the protein in question as the denominator in $p$. Due to the relative nature of this formulation, what matters is the fold of the protein - i.e. is the size of a given region (say the protein core) typical of a protein of a given size? This would imply differences in interpreting these statistics for, e.g., globular vs non-globular proteins.

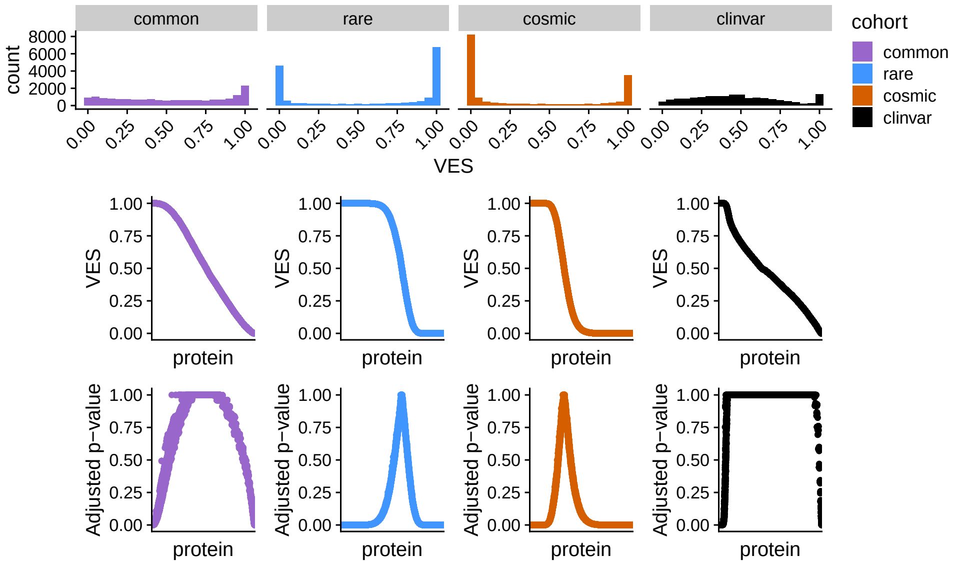


Figure 3: Distributions of VES and associated p-values calculated on individual proteins for gnomAD common, rare, COSMIC and ClinVar. (*top*) Distribution of whole-protein VES in the four datasets. (*bottom*) VES and associated p-values for each protein the four datasets. For each dataset, proteins are ranked by their VES, such that the horizontal axes for the VES and p-value scatterplots are identical to one another.

We have also generated distributions and overviews of the VES calculated on the ClinVar, COSMIC, gnomAD common and rare variant sets (Figure 3 above). Histograms of VES typically has peaks around VES = 0 and 1, indicating subsets of entities with pervasive enrichment and depletion of variants. The shapes of these histograms are different for the ClinVar and gnomAD common sets, probably related to the sparsity of variants recorded for both datasets (see main text Table 1). We think considering the VES coupled with their associated p-values would help in interpreting variant enrichment/depletion, in that

extreme VES (i.e. close to 0 or 1) are always coupled with low adjusted p-values (Figure 3); these two metrics could help in identifying significantly enriched/depleted entities regardless of the shape of the VES distributions in general.

This figure is now included as Supplementary Figure S8 and referenced in the main text (lines 214-215).

6. The PC plots are somewhat difficult to follow. In figure 3A, I presume the authors ran the VES across all proteins for each of the four variant sets, then ran PCA on the GSEA scores for each of those sets? Each data point represents a pathway within the enrichment space? A detailed caption enumerating the steps toward this figure would be helpful.

   This interpretation is correct. We improved Figure 3 by adding an illustrative panel (new Figure 3A) which details the steps taken to perform GSEA.

7. Figures 4 and 5 seem to contradict one another. Looking at figure 4 there appears to be no enrichment or depletion in the surface or core features when you look at the pathway level (presumably scored by VES?), but in figure 5 when you use a different density metric at the protein level, the authors claim there is evidence of this - how do you describe this discrepancy? It is also unclear why the authors are using both metrics (VES and density) instead of focusing on one, or integrating the two more fully. It would also be helpful to know the correlation between the VES and density-based metric.

   (We would first like to point out we have moved Figure 4 to Supplementary Figures (see below - response to Reviewer 2 Point 12), so "Figure 4" below refers now to Supplementary Figure S4, while "Figure 5" below refers to the new Figure 4.)

   Figure 4 is looking at the enrichment of specific pathways - i.e. we are asking this question: in what pathways are proteins enriched in variants in the core (or surface/interface) involved? The metric here is a conventional GSEA score. There we showed that population variants tend to map to surfaces of proliferation-related proteins. It is not quantifying whether the surface/core *in general* are depleted or enriched in different variants. This is addressed in Figure 5, where we look at **all** proteins with structural coverage and measure the tendency for variants localise to each region. Figures 5A and B clearly demonstrate different tendencies for population and pathogenic variants to localise to protein surfaces and cores. This is *not* the VES; we are plotting a density metric (Equation 2 in the Methods), which is labelled $\omega$. Depending on the "scope" of the question (enrichment in a single protein vs multiple proteins), we chose the appropriate metric to display the data. In theory, the formulation of the binomial model (see above) could be extended to describe variant distribution across multiple proteins (e.g. enrichment in surface regions of *all* proteins in the proteome *together*). We have not explicitly verified this in this work and therefore the density was plotted in these cases. We are consistent in always using the VES metric when we examine a single protein/domain/domain-type (see e.g. main text Figure 5D, 6, 7), while showing this density metric when we are considering multiple proteins together.

   Nevertheless one can always calculate the variant density metric in one region of a single protein, and compare with the VES. These calculations were made available in the Supplementary Tables provided; below in Figure 4 we show the correlation between these two metrics for surface regions of each protein - each data point corresponds to the surfaces of one protein. As expected the two metrics show excellent positive correlation with one another. The strength of using the binomial distribution is that it provides a probabilistic framework for assessing enrichment/depletion. The binomial formulation explicitly takes into account the sizes of the regions and the absolute numbers of variants which localise to a given protein/entity.

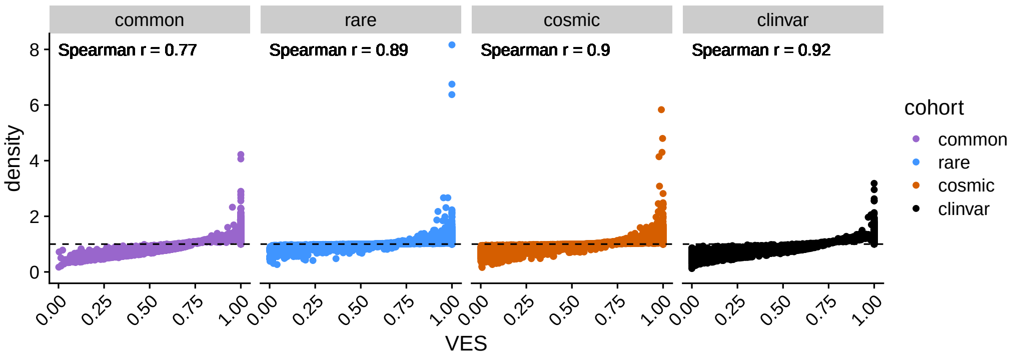


Figure 4: Comparison of VES and density metrics. Here we show, as an example, the variant enrichment statistics for surface regions of individual proteins in the four variant sets, where both VES and density metrics were calculated on the surface region of each protein. The Spearman correlation of the two statistics in each case is displayed. The dashed horizontal lines indicate variant density = 1 (i.e. the null case, with as much observed variants as expected).

Readers can easily inspect these for other regions by downloading the Supplementary Tables. This figure above (Figure 4) has been included as Supplementary Figure S9.

8. Figure captions should be improved to match the figures - several colours and labels are mis-specified, and some inline references to figures point to the wrong ones (line 587). Apologies - these have now been corrected.

9. For the population-level variation, the interpretation of enrichments is difficult to follow - these are proteins that are susceptible to natural variation? Would the opposite enrichment not be more interesting - protein regions that are devoid of variation?

   These could be proteins which are robust to variation (structurally and functionally), are redundant in function, or for which variation is desirable in order to respond to different environments. Our quantification still follow the concept of enrichment - in the sense that we have more-than-expected population variants in such proteins. Proteins (or regions) devoid in population variation is of course interesting especially in the case of discovering pathogenic variants. However, this is not to say that the proteins susceptible to natural variations are not "interesting" - they could be interesting to, e.g. drug discovery pipelines, as these commonly-observed variants could modulate drug response for a large proportion of the population, a point which we have made in the Discussion section. As a general resource we aim at serving users with different needs; we believe our formulation of variant "enrichment" is general enough for this purpose.

10. What is the sensitivity of the enrichment test for extremely low counts? It seems likely that some proteins may harbor only one or two variants from some of these sets - especially for sub-regions of the protein.

    For a low number of variants, the sensitivity of VES will indeed decrease, since given a small $n$, the binomial cumulative distribution function (CDF) - i.e. VES - will be subjected to large variations as $k$ changes. Associated $p$-values (which we showed above in Figure 3 in this document) as well as the actual number of variants to each protein and region help users in interpreting VES; users can easily obtain these statistics by querying the database online. We also offer such calculation on-the-fly for user-submitted variants.

11. While the comparisons to CADD and REVEL are encouraging as a positive control, doesn't this result also imply that these scores are classifying pathogenicity well without using protein-structure information? Furthermore, it seems likely that these scores were

trained in part from clinvar variants - what does this analysis provide that comparisons to Clinvar do not?

Firstly, both CADD and REVEL are ensemble methods integrating individual variant impact prediction tools. Based on these results models were trained (CADD: Support Vector Machine [SVM]; REVEL: Random Forest [RF]) on defined variant subsets to yield predicted scores for variant impact. It is not entirely correct to say that these two methods do not use protein structure information: some of the tools included in CADD and REVEL have incorporated different protein-level features in impact prediction, for examples:

- PolyPhen: Used in REVEL and CADD - secondary structure (from DSSP), $\phi - \psi$ dihedral angles, residue B-factor etc., for proteins which could be mapped to PDB structures using BLAST [8].
- "Grantham": A formula included in CADD which describes the change in chemical character upon an amino acid change [9]. Shown to correlate best with the substitution frequencies observed in evolution.

On the point related to ClinVar, REVEL was trained on HGMD variants; over half of ClinVar variants could be found in HGMD [10]. CADD was *not* trained on ClinVar - in fact it was not designed to distinguish between pathogenic and non-pathogenic variants. Instead model training utilised a dataset of human-chimpanzee variants and a similarly-sized set of simulated variants [11]. The purpose of including comparisons with CADD and REVEL is to act as a validation step to illustrate that the protein-level features we analysed do hold explanatory power to identify impactful variants. This is also to address a possible criticism that our observed differences could be dependent on our classification of variant sets (i.e. ClinVar, COSMIC, gnomAD), simply binning variants based on the occasion that they were observed. By using an external scoring method which was not *explicitly* trained on the features we analysed, we consider that a validation to our approach.

12. The emphasis on pathway-related figures and content seems to distract from the more biochemical points of the manuscript. The effect of a variant on the biochemical properties of a protein is already complex and difficult to explain - this is compounded by the pathway-level analyses, and other more interesting associations (like the PTM and VES) are more interesting from a protein perspective, but this is relegated to supplementary figures.

While we agree the manuscript might be too dense and packed, we consider pathway-related analyses to be an important aspect of this work, and would argue for these to be kept in the main text. Some of the observations there are perhaps not surprising: for example, olfactory receptors have been considered as negative controls to benchmark variant impact annotation tools (e.g. REVEL and CADD [10, 11]). Moreover, in terms of somatic variants, those enhancing proliferation and cell survival are classifically considered to be important cancer drivers [12]. These associations at individual pathway/function level are not novel, the value of our work is in considering ClinVar, COSMIC and gnomAD variants in a *uniform* and *unbiased* manner, and in demonstrating that using solely enrichment for our examined variant sets we can clearly segregate KEGG pathways. This is an important - and, to our knowledge - novel finding which could only be possible under the unbiased approach to quantify variant enrichment that we took, and therefore deserves to be highlighted in this manuscript.

The inclusion of pathway-analyses also serve to illustrate the point that more "macroscopic" features at the level of pathways and functions, as opposed to features closer to

the atomic level (such as structural features), could also contribute to distinguishing variant impact. This has been not explored in other studies. We have shown here that these features do hold discriminatory power to distinguish pathogenic from non-pathogenic variants. These could be useful in guiding readers to explore the underlying mechanisms in which a variant poses impact.

For the above two reasons we think our pathway-related results are important, and not less "interesting" than the other results Reviewer 2 pointed out. While the data on PTMs are interesting, due to the sparsity of PTM annotations they are often associated with large confidence intervals. We opted to focus on other features with more prominent differences between datasets.

However, we do acknowledge that in the previous edition the pathway analyses might be difficult to follow, and lead to some confusion from reviewers; we think the previous Figure 4 and the related discussion could be relegated to Supplementary Materials (now section "Functional distinction of variant enrichment specific to protein structural regions", Supplementary Results).

I would like to re-emphasize that the work presented is important and interesting and does make several points worth contributing to the literature as a resource - but the current presentation of the results doesn't quite do it justice, and leaves the reader unclear how to use such a resource in future work.

We thank the reviewer for recognising the significance of this work. As detailed above we have implemented several changes to the organisation of the manuscript, emphasised novel findings which are not present in other analyses/databases, and described the significance of several aspects of our analysis which the reviewer has posed doubts. Together with a clarified presentation on introducing the VES statistic and revised versions of the introduction and discussion, we hope readers will find it easier to follow and use ZoomVar as a resource.

## Reviewer 3

Rob Ewing – note that this reviewer has waived anonymity

### Overview

Laddach et al have performed a comprehensive analysis of missense variants. This analysis focuses on comparison of missense variants in proteins associated with disease (cancer), germline disease and variants present in the wider population. An important motivation of the study is to understand missense variants and the effects they have so as to better be able to distinguish variants that are associated with disease. The paper consists of detailed analyses of protein missense variants across the 3 contexts and release of a database resource.

The study identifies some interesting correlations and findings which will be of general interest. In addition, this paper is anice example of how integration of different data can be used to ask important and wide ranging biological questions. The authors indicate that this is the first example of using proteomics data as measurements of stability and abundance to explore how missense variants are associated with these properties. As a PLoS Biology resource, the paper, associated data and the database will be of immediate use to others. The work is also very well explained. In particular the authors have made good efforts to explain the computational techniques used in a clear graphic form. This will aid understanding and accessibility in particular for non-computational biologists.

We thank Dr. Ewing for his constructive and favourable comments.

## Specific Comments

1. Clarifying the variant enrichment calculation. Please indicate whether the method differs from previously published. Also clarify or explain - the model assumes that each amino-acid in each protein has an equal probability of accumulating a missense variant? Also are the entities in Figure 1 only proteins or also protein domains? If so - is there normalization for the length of the domains as there is for the length in AA of each protein?

   First we would like to point to reviewer to our response to Reviewer 1 above which contains a detailed explanation to motivate our method of variant enrichment calculation. As we mentioned above, the method is taken from [1] - identical but extended to look not just at interfaces, but in different "levels" and "regions" of the protein anatomy (as we defined in the main text).

   Reviewer 3's assumption is correct; to add to this, it is also assumed that the acquisition of each variant is independent from one another; this is implicit in the binomial setting. In Figure 1, the method applies to both protein and protein domains. In either case, normalisation for lengths ("sizes" in our terminology referring to the discussion above in our answers to Reviewers 1 and 2) were achieved in formulating the $p$ in the binomial formula. Table 3 below shows the meaning of "entity" in different scenarios.

| Problem | "Entity" | Total | $p$ in VES formula |
|---|---|---|---|
| Variant enrichment in Protein X | Protein X | All proteins | $\frac{\text{Size of protein X}}{\text{Size of all proteins}}$ |
| Variant enrichment in the core of Protein X | Core of Protein X | Protein X | $\frac{\text{Size of Core in X}}{\text{Size of protein X}}$ |
| Variant enrichment in Domain Y | Domain Y | All domains | $\frac{\text{Size of domain Y}}{\text{Size of all domains}}$ |
| Variant enrichment in core of Domain Y | Core of Domain Y | Domain Y | $\frac{\text{Size of core in Y}}{\text{Size of domain Y}}$ |

Table 3: Definition of "entity" in different scenarios, along with formulation of $p$ in the binomial VES formula.

   As you can see in the table, in each case normalisation is performed for the length in AA. We think this Table 3 above is useful to grasp the definition of the VES calculation in different scenarios; it is now included as a panel in main text Figure 1 (new Figure 1C).

2. It was not clear exactly how GSEA was used in the context of variant enrichment. Were proteins ranked according to VES? Should 'substrating' read 'subtracting'? (line 184)

   This was addressed above in response to Reviewer 2's comments (#6). Apologies for the typo on "subtracting" - this has now been corrected.

3. Figure 7B is too small - in particular because there are several significant results shown it should be larger.

   Panels in Figure 7 have now been resized.

4. Is there a previous rationale for classifying regions close to PTMs if within 8 angstroms? (Line 113)

This has commonly been used for defining regions close to PTMs, in e.g. [4]. We have now explicitly stated this (line 146).

5. Add more explanation for Equation 2 .. what X is etc

   More detailed explanation for Equation 2 has now been added (lines 221-225).

## References


[1] Porta-Pardo E, Garcia-Alonso L, Hrabe T, Dopazo J, Godzik A. A Pan-Cancer Catalogue of Cancer Driver Protein Interaction Interfaces. PLoS computational biology. 2015;11(10):e1004518. doi:10.1371/journal.pcbi.1004518.

[2] Gress A, Ramensky V, Kalinina OV. Spatial distribution of disease-associated variants in three-dimensional structures of protein complexes. Oncogenesis. 2017;6(9):e380. doi:10.1038/oncsis.2017.79.

[3] Engin HB, Kreisberg JF, Carter H. Structure-Based Analysis Reveals Cancer Missense Mutations Target Protein Interaction Interfaces. PloS one. 2016;11(4):e0152929. doi:10.1371/journal.pone.0152929.

[4] Stehr H, Jang SHJ, Duarte JM, Wierling C, Lehrach H, Lappe M, et al. The structural impact of cancer-associated missense mutations in oncogenes and tumor suppressors. Molecular cancer. 2011;10:54. doi:10.1186/1476-4598-10-54.

[5] Gao M, Zhou H, Skolnick J. Insights into Disease-Associated Mutations in the Human Proteome through Protein Structural Analysis. Structure. 2015;23(7):1362–9. doi:10.1016/j.str.2015.03.028.

[6] David A, Razali R, Wass MN, Sternberg MJE. Protein-protein interaction sites are hot spots for disease-associated nonsynonymous SNPs. Human mutation. 2012;33(2):359–63. doi:10.1002/humu.21656.

[7] Sivley RM, Dou X, Meiler J, Bush WS, Capra JA. Comprehensive Analysis of Constraint on the Spatial Distribution of Missense Variants in Human Protein Structures. American journal of human genetics. 2018;102(3):415–426. doi:10.1016/j.ajhg.2018.01.017.

[8] Adzhubei IA, Schmidt S, Peshkin L, Ramensky VE, Gerasimova A, Bork P, et al. A method and server for predicting damaging missense mutations. Nat Methods. 2010;7(4):248–249.

[9] Grantham R. Amino acid difference formula to help explain protein evolution. Science. 1974;185(4154):862–864.

[10] Stenson PD, Mort M, Ball EV, Chapman M, Evans K, Azevedo L, et al. The Human Gene Mutation Database (HGMD): optimizing its use in a clinical diagnostic or research setting. Hum Genet. 2020;139(10):1197–1207.

[11] Kircher M, Witten DM, Jain P, O'Roak BJ, Cooper GM, Shendure J. A general framework for estimating the relative pathogenicity of human genetic variants. Nature Genetics. 2014;46(3):310–315.

[12] Stratton MR, Campbell PJ, Futreal PA. The cancer genome. Nature. 2009;458(7239):719–724.
