# Supplementary material for: Pathogenic missense protein variants affect different functional pathways and proteomic features than healthy population variants
Source: PLoS Biol. 2021 Apr 28;19(4):e3001207. doi: 10.1371/journal.pbio.3001207 (PMC8110273; doi:10.1371/journal.pbio.3001207)
Supplement: Attachment — Submitted filename: reviewer_response.pdf [file pbio.3001207.s038.pdf]

Response to reviewers (second round of revision)

March 3, 2021

Pathogenic missense protein variants affect different functional pathways and proteomic features than healthy population variants
Anna Laddach, Joseph Chi-Fung Ng & Franca Fraternali

We would like to thank for the constructive comments from the reviewers. Here we include a point-by-point response to these comments (reproduced here, highlighted in red). Changes in the manuscript are highlighted in blue.

Wherever possible we have referenced the relevant line numbers where changes have been made to the main manuscript; these were taken from the revised version with track changes.

We would first like to point out that following the editor's suggestions the title and abstract have been modified slightly. In response to the editor's reminders on PLoS Data Policy, data files underlying all figures are included as Supplementary Data. Protein- and domain-level VES statistics are also readily available by querying the ZoomVar database online.

Reviewer 1

The authors of Laddach, Ng et al. have addressed most of my major concerns. In particular, their supplemental note explaining the formulation of their Variant Enrichment Score (VES) greatly helps in the understanding and ultimately the interpretation of the score. I do still believe the main text describing the VES calculation is substantially dense that the supplemental note is required rather than the main text standing alone.

We are glad to learn that Reviewer 1 is satisfied with our effort to explain the formulation of VES. We have further simplified the explanation of VES in the main text (lines 168-184 in the Materials and Methods section; lines 281-286 in the Results section) and referenced the supplemental note.

Minor comments:

1. In the supplemental note, Table 1 defines the parameters used in the two approaches. Approach 2 defines n, k and p in terms of mutated residues rather than variants. Since the VES calculation ultimately uses variants, it would be worth to include another column of parameter definitions with n, k, and p in terms of variants.

We understand the rationale of this suggestion and agrees that defining these parameters in terms of variants is more straightforward to understand; it is also more intuitive considering that the text explains the two approaches in terms of variants. We have reworded the definitions

of n, k and p in terms of variants in Table S1. They were not included as an additional columns but rather to replace the existing wordings as we aim to keep a simple explanation of parameter definitions.

This table (Table S1 in Supplementary Materials) is reproduced here for your convenience:

	**Approach 1**	**Approach 2**
n	Number of residues in the region	Total number of variants
k	Number of variants in the region	Number of variants in the region
p	$\frac{\text{Total number of variants}}{\text{total number of residues}}$	$\frac{\text{number of residues in the region}}{\text{total number of residues}}$

Table 1: Summary of the parameters (n, k and p) of the binomial model in the two approaches explained above.

2. The bubble sizes in Figure 5 corresponding to adjusted p-values are difficult to distinguish. In particular, the difference of sizes between 0.05 and 0.25 bubbles is negligible while it is a large difference in terms of significance.

We apologise for the difficulty in interpreting Figure 5. We have now re-generated this figure (now numbered as Figure 4 after reorganising the flow of the paper, in response to a suggestion from Reviewer 2; see below) adding an additional bound (adjusted p-value = 0.1) in between 0.05 and 0.25 to help distinguishing between data points of different levels of significance. Following the editor's reminders on PLoS Data Policy, we have also included the data tables containing VES and p-values so that readers could refer to for exact values.

Reviewer 2

The authors have responded in detail to prior questions and critiques, and have improved several aspects of the manuscript. I would still strongly argue for clarifying the manuscript to highlight the primary points in the results section as it is still difficult to follow the logic of the manuscript and understand the most important aspects of the work. In the response, the authors argue that the two main points are "the unbiased comparison of structure and pathway features targeted by missense variants, and the examination of proteomics features in distinguishing missense variants", but I don't feel like either of these primary points are addressed in the six sections of the results. As it reads currently, the authors present and validate their approach with oncogene and tumor suppressor gene analyses in 4.1, then jump to pathway-level analyses in 4.2, then back to protein-feature analyses in 4.3... if the authors wish to highlight the methodological strengths of their approach, tighten the results to specifically highlight all the ways in which the VES score is finding patterns that are supported by prior literature, either with specific protein examples, or with general trends in domains. This could be followed by sections that enumerate unexpected findings that are novel discoveries of the approach, followed with pathway-level enrichments. I only suggest this structure because it would clarify what the authors are presenting as NEW discoveries versus the findings that are largely congruent with what would be expected based on biochemistry/biology. I would also suggest renaming results section headers to more specifically highlight the new knowledge the authors are adding to the literature with their analyses. As it is currently presented, the authors seem to enumerate various different enrichments with little context on if these were expected

or novel.

We thank Reviewer 2 for this helpful suggestion on restructuring the paper. We agree that this could highlight better the strengths of our method, as well as new findings enabled by the method. In the new version we have reorganised the paper in the following manner (the relevant section numberings in the submitted, revised version are referenced here):

- First, a presentation of our framework and validation with oncogene and tumour suppressor genes as a known, previously explored example. (section 4.1)
- Second, discussion of variant enrichment trends across protein structural regions (surface, core, interface) and domains. This illustrates the point that our method extends patterns of variant enrichment (which were previously explored [i] in smaller datasets and/or [ii] without a robust probabilistic quantification) to large, up-to-date variant datasets, and enables a systematic and statistical evaluation of these tendencies of variant enrichment in proteins and domains. (New section 4.2, "Robust statistical quantification confirms and extends variant enrichment patterns in large variant datasets", under which materials on protein regions and domains are grouped into two sub-sections.)
- Third, illustration of our method to study previously underexplored features that distinguish pathogenic and population variants. This includes our analyses of functional pathways, and the association of variant enrichment with proteomics features. (New section 4.3, "Functional and proteomics features distinguish variants in health and disease", under which materials on functional pathways and proteomics features are organised into two sub-sections.)
- Finally, application of our method to explore the continuous spectrum of pathogenicity, both in terms of the "rarity" of population variants, and in validating our features using orthogonal variant impact annotation from REVEL and CADD. (section 4.4)

This is accompanied with changes in section headings which should better highlight the gist of our findings. We hope this restructuring enhance the discussion on both the strength of our method, and the novelty of the analyses.

Reviewer 3

No further comments.

We thank Reviewer 3 for his previous contributions.
