# Supplementary material for: Pathogenic missense protein variants affect different functional pathways and proteomic features than healthy population variants
Source: PLoS Biol. 2021 Apr 28;19(4):e3001207. doi: 10.1371/journal.pbio.3001207 (PMC8110273; doi:10.1371/journal.pbio.3001207)
Supplement: Attachment — Submitted filename: response.docx [file pbio.3001207.s039.docx]

**Point-by-point response**

*Pathogenic missense protein variants affect different functional pathways and proteomic features than variants in the healthy population*

Anna Laddach, Joseph Chi-Fung Ng & Franca Fraternali

In the latest correspondence we do not have fresh comments and suggestions from reviewers to address. Here we detailed editorial requests and respond to these issues (the editorial requests are reproduced here in red). Wherever possible we have indicated relevant changes made in the manuscript.

Thank you for submitting your revised Methods and Resources entitled "Pathogenic missense protein variants affect different functional pathways and proteomic features than healthy population variants" for publication in PLOS Biology. I have now discussed it with the editorial team.

Based on the reviews, we are now satisfied with your responses to the remaining issues raised by the reviewers and will probably accept this manuscript for publication. However, we would like you to clarify some points on the data that are confusing. While all the data underlying the graphs shown in the figures seem to be included, some of the notes made in the figure legends don't match the data shown in the files. Here are a couple of examples:

- In Fig. 3 legend, you mention that the data can be found in Data S2, but in that file you only specify data for Fig. 4, Fig. 5, Fig. 7, Fig. S4, Fig. S5, Fig. S6, Fig. S8, Fig. S9, Fig. S11, Fig. S17 and Fig. S12

- In Fig. 5 legend, you mention the data can be found in Data S6, but in that file, it’s only specified data for Fig. 3, Fig. 7 and Fig. S10.

We apologise for discrepancies with regards to references to Supporting Data files. This has arisen from rearrangements of sections as requested by Reviewer 2 in the previous review from which these references to Supporting Data files had not been amended accordingly. In both cases we have identified that the references within those data files were wrong (Data S2 should specify data for Fig. 3 instead of Fig. 4; Data S6 should specify data for Fig. 5 instead of Fig. 3). In both cases the references within these data files have been amended. See below for more detailed description of checks made to other references to Supporting Data files.

We would like you to check all the figure legends and data files carefully and do the following:

- Please include in the manuscript a list of all you data files and specify clearly what figures show the data contained in each of them.

- Please make sure that the correct data file is indicated in the corresponding figure legends.

We have now double-checked all supporting data files and their in-text references to make sure figure legends, in-text references and indications in the data files agree with one another. All numberings now conform to their corresponding data files. As requested we have also provided a list of data files towards the end of the revised manuscript (Section 7, main manuscript).

As you address these items, please take this last chance to review your reference list to ensure that it is complete and correct. If you have cited papers that have been retracted, please include the rationale for doing so in the manuscript text, or remove these references and replace them with relevant current references. Any changes to the reference list should be mentioned in the cover letter that accompanies your revised manuscript.

We have checked through our bibliography and have made these minor changes:

- *Formatting:* Reference 65 has been updated to show author names properly.
- *Removal:* Original references 75,76 are entries in the Pfam database. We have now removed these references and instead referred to the relevant accession identifiers in-text (line 365, clean version of revised manuscript).

*Other edits*

We also took this opportunity to make the following minor stylistic and grammatical edits:

1. *Formatting:* We have made changes to conform to the PLoS style in labelling figures (‘Figure 1’ 🡪 ‘Fig 1’; ‘Supplementary Table S1’ 🡪 ‘S1 Table’, etc.), and renamed the Supplementary Materials to ‘Supporting Information’, in line with the PLoS Authors Guide.
2. *Spell-check and grammar-check:* Both main text and supporting information has been spell-checked and grammar-checked in this re-submission.

We have included both the clean version of the manuscript (PDF and LaTeX Source files) and a version with track-changes to reflect these modifications.

We hope these changes address your requests and you will find this revised edition ready to be accepted for publication.
